# A pan-cancer analysis of synonymous mutations

**DOI:** 10.1038/s41467-019-10489-2

**Published:** 2019-06-12

**Authors:** Yogita Sharma, Milad Miladi, Sandeep Dukare, Karine Boulay, Maiwen Caudron-Herger, Matthias Groß, Rolf Backofen, Sven Diederichs

**Affiliations:** 1 Division of Cancer Research, Department of Thoracic Surgery, Medical Center, University of Freiburg, 79085 Freiburg, Germany; 2 Faculty of Medicine, University of Freiburg, 79085 Freiburg, Germany; 3 German Cancer Consortium (DKTK), 79106 Freiburg, Germany; 4 Bioinformatics Group, Department of Computer Science, University of Freiburg, 79110 Freiburg, Germany; 5 Division of RNA Biology and Cancer, German Cancer Research Center (DKFZ), 69120 Heidelberg, Germany; 6 National Center for Tumor Diseases (NCT), 69120 Heidelberg, Germany; 7 Signalling Research Centres BIOSS and CIBSS, University of Freiburg, 79104 Freiburg, Germany

**Keywords:** Cancer genetics, Molecular biology

## Abstract

Synonymous mutations have been viewed as silent mutations, since they only affect the DNA and mRNA, but not the amino acid sequence of the resulting protein. Nonetheless, recent studies suggest their significant impact on splicing, RNA stability, RNA folding, translation or co-translational protein folding. Hence, we compile 659194 synonymous mutations found in human cancer and characterize their properties. We provide the user-friendly, comprehensive resource for synonymous mutations in cancer, SynMICdb (http://SynMICdb.dkfz.de), which also contains orthogonal information about gene annotation, recurrence, mutation loads, cancer association, conservation, alternative events, impact on mRNA structure and a SynMICdb score. Notably, synonymous and missense mutations are depleted at the 5'-end of the coding sequence as well as at the ends of internal exons independent of mutational signatures. For patient-derived synonymous mutations in the oncogene *KRAS*, we indicate that single point mutations can have a relevant impact on expression as well as on mRNA secondary structure.

## Abstract (web summary)

Synonymous mutations do not alter amino acid sequence but may exert oncogenic effects in other ways. Here, the authors present a catalogue of synonymous mutations in cancer and characterise their properties.

## Introduction

According to GLOBOCAN, cancer is a main cause of morbidity and mortality worldwide with 14.1 million new cases and 8.2 million deaths in 2012^1^. Cancer genomics studies with vast collaborative efforts by The Cancer Genome Atlas (TCGA)^2^ and the International Cancer Genome Consortium (ICGC)^3,4^ have identified numerous mutations in cancer, but information on their pathological relevance is often lacking. As an important resource for cancer genome information, the Catalogue of Somatic Mutations in Cancer (COSMIC) aims at annotating all somatic mutations in human cancer^5,6^. COSMIC contains mutations that are manually annotated for individual genes from publications, as well as datasets derived from large-scale whole genome sequencing.

Chromosomal losses and gains or missense and nonsense point mutations altering tumor suppressor genes and oncogenes are widely studied^7^. However, also genetic changes leaving the protein sequence intact can significantly impact cancer genes^8^, e.g., by affecting the translation, RNA structure or stability of the mutated transcript.

Synonymous mutations do not alter the amino acid encoded by the affected codon due to the degeneracy of the genetic code, but change the DNA and RNA sequence. They were viewed as silent mutations and were hence mostly overlooked in cancer genetics. Early studies assumed that these were not under selective pressure^9^, but later studies found synonymous mutations to be subject to natural selection in different species^10–12^.

Synonymous mutations play a role in many human diseases^13^ and can correlate with the clinical outcome or therapy response^14–16^. In cancer, synonymous mutations are estimated to represent 6–8% of all driver mutations occurring due to single nucleotide substitutions^17^. Synonymous substitutions are enriched in oncogenes but no evidence for selection is found in tumor suppressor genes (except *TP53*)^17^.

In contrast to their perception as silent mutations, synonymous mutations can change protein levels or protein conformation by altering splicing regulatory sites, mRNA stability, miRNA binding sites or translation efficiency^13^. Synonymous mutations in *TP53, BRCA1, BRCA2* and *APC* lead to exon skipping and change in protein structure by creation or inactivation of a splice site^18–21^. A synonymous substitution increases the mRNA stability of *BCL2L12* due to the loss of a miRNA target site^22^. Synonymous mutations can alter the secondary structure of an mRNA affecting its stability or translation^23,24^. However, no changes in RNA secondary structures of cancer genes have been proven so far. Synonymous mutations can change the translational speed by creating ribosomal pause sites affecting the cotranslational protein folding^25^. A synonymous mutation in *MDR1* introduces a rare codon slowing down translation and allowing cotranslational folding altering its substrate specificity^26^. Synonymous codons in gamma-B-crystallin modulate translation and cotranslational folding^27^. Lastly, a synonymous mutation in p53 prevents the phosphorylation of its nascent peptide chain^28^.

Here, we provide and analyze a comprehensive resource of 659,194 synonymous mutations in human cancer, SynMICdb, which contains information on and allows specific searches for their frequency, tumor distribution, evolutionary conservation, position in the coding region, association with alternative events, as well as their impact on the mRNA secondary structure. It enables researchers to comprehensively study synonymous mutations in their gene or tumor entity of interest. We additionally provide experimental evidence for the impact of synonymous mutations on the expression and the secondary structure of the oncogene *KRAS*.

## Results

### Properties of synonymous mutations in cancer

**Fig. 1:**
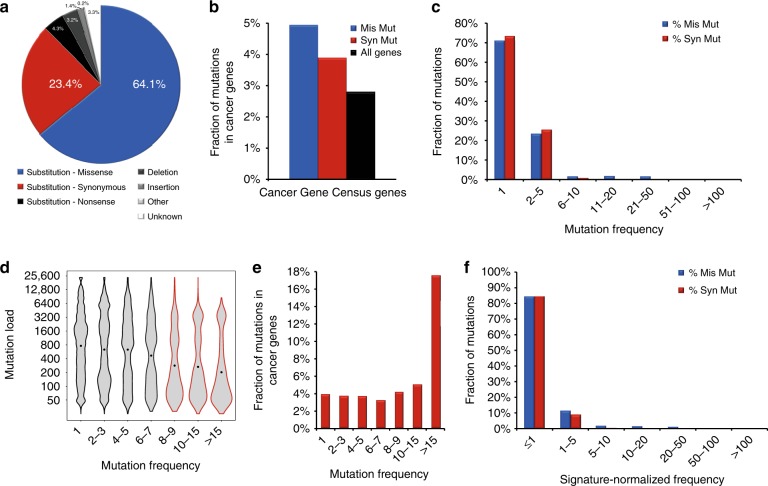
Properties of synonymous mutations in cancer. **a** Synonymous mutations are the second most frequent class of point mutations in cancer. **b** Synonymous mutations (Syn Mut) and missense mutations (Mis Mut) are enriched in cancer-associated genes compared with the proportion of annotated cancer genes among all human coding genes (All Genes). **c** Synonymous mutations (Syn Mut) display a similar recurrence pattern as missense mutations (Mis Mut) with more than 25% of mutations found recurrently in more than one sample. **d** The violin plot depicts the distribution of the mutation loads of the samples associated with different frequencies of the synonymous mutations with the median indicated by a dot. **e** The fraction of synonymous mutations in known cancer-associated genes increases for highly recurrent synonymous mutations. **f** The frequency of synonymous mutations (Syn Mut) and missense mutations (Mis Mut) are normalized to a mutation signature to account for the mutation bias in cancer cells

To gain a comprehensive dataset of synonymous mutations, we examined 3.88 million mutations identified in whole genome sequencing studies of tumor tissues and cell lines in a pan-cancer analysis including but not limited to TCGA and ICGC data as deposited in COSMIC. After curation of the dataset for duplicates and annotation errors^29^, 2.81 million mutations remained in 20,414 human genes of 18,028 samples from 88 different tumor entities. In this dataset, we found 659,194 synonymous mutations . Hence, synonymous mutations were the second most frequent type of point mutation (23.4%) after missense mutations (64.1%), but more frequently listed than nonsense mutations, deletions or insertions (4.3%, 3.2%, 1.4%) (Fig. 1a, Supplementary Data 1). While the latter were widely characterized as tumor-causing, synonymous mutations have hardly been studied.

The 659,194 synonymous mutations mapped to 19,916 genes derived from 13,935 tumor samples after data curation (Supplementary Data 2). To integrate orthogonal datasets, we added numerous characteristics to enable easy and focused searches. Based on this platform, we compared synonymous (syn) and missense (mis) mutations and found striking parallels but also differences.

Our data collection revealed that known cancer genes from the Cancer Gene Census (2.8% of all genes)^30^ were enriched in synonymous, as well as in missense mutations (3.9% vs. 4.9%) (Fig. 1b). In turn, more than 95% of both types of mutations were found in genes not yet associated with cancer leaving room for discoveries. Somatic synonymous and missense mutation catalogs contained a similar fraction of known Single Nucleotide Polymorphisms (SNPs, 8.1% vs. 8.3%).

176594 synonymous mutations were found recurrently across all tumor entities—similar to the recurrence fraction of missense mutations (26.8% vs. 29.1%, Fig. 1c). The most frequent synonymous mutation was found 63 times (*NCOA6* c.807 G > A), while the most frequent synonymous mutation never listed as SNP was found 45 times in the tumor suppressor *CHEK2* c.1176 G > T. At the gene level, the large gene *TTN* was found most often with 2253 cancer samples, while normalized for gene length, *KCNJ12* was found most often with 278 occurrences. Importantly, the frequency of a mutation negatively correlated with the mutation load, i.e., the total number of mutations found in a tumor. Thus, highly recurrent synonymous mutations were more likely found in tumors with overall lower mutation rates potentially indicating a higher specificity (Fig. 1d). Similarly, highly recurrent synonymous mutations were enriched in known cancer genes (Fig. 1e).

Next, we added a conservation score as it may reflect functional relevance or localization in a regulatory motif. We found more than 40% of the synonymous mutations affecting highly conserved nucleotides (PhastCons score > 0.9), while missense mutations were even more frequently affecting highly conserved residues (67%) (Supplementary Fig. 1a).

The nucleotide changes leading to synonymous mutations were highly similar to missense mutations with C > T/G > A changes accounting for 67% (Supplementary Fig. 1b). This mirrors the known mutation bias of CGC Mutation Signature 1, which is prevalently found across all tumor entities^31^. Based on this mutation bias, we normalized the frequency of each mutation to its signature-based probability, i.e., we multiplied the frequency with (1-probability from signature) to generate the signature-normalized frequency (Fig. 1f).

In contrast to their similarity regarding nucleotide changes, the distribution of synonymous and missense mutations differed on the amino acid level. When correcting for the number of codons for each amino acid and the total number of mutations, we found that missense mutations were enriched in codons for charged amino acids like glutamic acid (E), whereas synonymous mutations were enriched for hydrophobic amino acids like phenylalanine (F) (Supplementary Fig. 1c). An independent approach excluded that these differences were due to mutation bias: for each codon, we combined its total number in the human transcriptome with the mutation signature resulting in a number of expected synonymous mutations aggregated to the amino acid level (Supplementary Fig. 1d). The comparison of the expected number of synonymous mutations to the actual number found in cancer revealed striking differences (Supplementary Fig. 1e) and independently validated the enrichment of synonymous mutations in phenylalanine codons and their depletion in glutamic acid codons. Thus, synonymous mutations were not randomly distributed across the encoded amino acids.

Furthermore, 5616 synonymous mutations mapped to clinically relevant variants from ClinVar^32^. ClinVar mostly comprises hereditary diseases and only few associations with tumors, but nonetheless 470 synonymous mutations were associated with tumors in ClinVar (Supplementary Data 3).

**Fig. 2:**
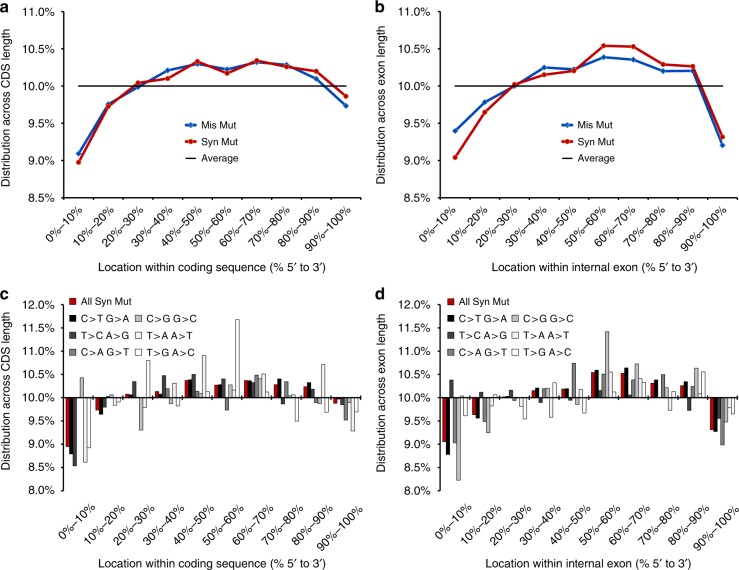
Depletion of mutations towards the ends of coding regions and exons. **a** The distribution of the positions within the coding region of all mutations in all affected genes independent of their length is depicted in 5'-to-3' direction. The black line at 10% frequency would indicate equal distribution along the 10 bins of the coding sequence length. Synonymous mutations (Syn Mut), as well as missense mutations (Mis Mut) are depleted towards the 5'-end of the coding region. **b** The distribution of mutations within internal exons of multiexonic transcripts is depicted in 5'-to-3' direction. The black line at 10% frequency would indicate equal distribution along the 10 bins along the internal exon length. Synonymous mutations (Syn Mut), as well as missense mutations (Mis Mut) are depleted towards both ends of the exon. **c** The distribution of synonymous mutations along the coding sequence is depicted separately for six groups of possible nucleotide changes in point mutations. A 10% frequency would indicate equal distribution along the 10 bins of the coding sequence length. **d** The distribution of synonymous mutations along the length of internal exons is depicted separately for six groups of possible nucleotide changes. A 10% frequency would indicate equal distribution along the 10 bins of the internal exon length

Next, we analyzed the distribution of mutations across the coding region of the gene. Synonymous and missense mutations were depleted in the first 10% of the coding sequence (Fig. 2a). Also, a significant depletion was observed in the first codons up to codon 50 with the strongest decrease in the first ten codons (Supplementary Fig. 2a).

Synonymous and missense mutations showed a similar distribution regarding their appearance in first, internal and last exons of the transcript or in monoexonic transcripts (Supplementary Fig. 2b). Within internal exons, synonymous mutations were evenly distributed with a notable depletion towards the exon-intron boundaries with a similar distribution pattern for missense mutations (Fig. 2b).

To exclude that the depletions of synonymous mutations towards the 5'-end of the coding sequence or both ends of internal exons were due to a mutation bias, we repeated the analysis for each nucleotide change separately and found the same depletion for four to six out of six types, respectively (Fig. 2c, d), indicating that these are not restricted to individual nucleotide changes.

We mapped synonymous and missense mutations to exons affected by events like alternative splicing or alternative promoter usage. In total, 133,320 combinations of synonymous mutations and alternative events were found with 58.7% representing cassette exons. These associations between synonymous mutations and alternative events may help to generate hypotheses for their potential impact (Supplementary Fig. 2c). Again, missense mutations showed a similar distribution.

To further characterize the impact of synonymous mutations on alternative splicing, we analyzed all wildtype and synonymous mutant sequences for the gain or loss of exonic splicing regulatory motifs. We derived exonic splicing enhancer (ESE) and exonic splicing silencer (ESS) motifs from two independent sources: RegRNA2.0^33^ and SpliceAidF^34^. We selected human exonic motifs and curated these sets for duplicate sequences and concatenated the information for each duplicate motif resulting in 76 motifs from RegRNA2.0 and 111 motifs from SpliceAidF (Supplementary Data 4). Searching for gains or losses of exonic splicing regulatory sites revealed that 26.8% of synonymous mutations caused a change in at least one predicted ESE or ESS motif (Supplementary Data 5). This table also lists the distance of the synonymous mutation to the closest exon boundary, which may also predict an impact on splicing. The individual changes for each synonymous mutation and each motif are listed for RegRNA2.0 (Supplementary Data 6) and SpliceAidF (Supplementary Data 7).

**Fig. 3:**
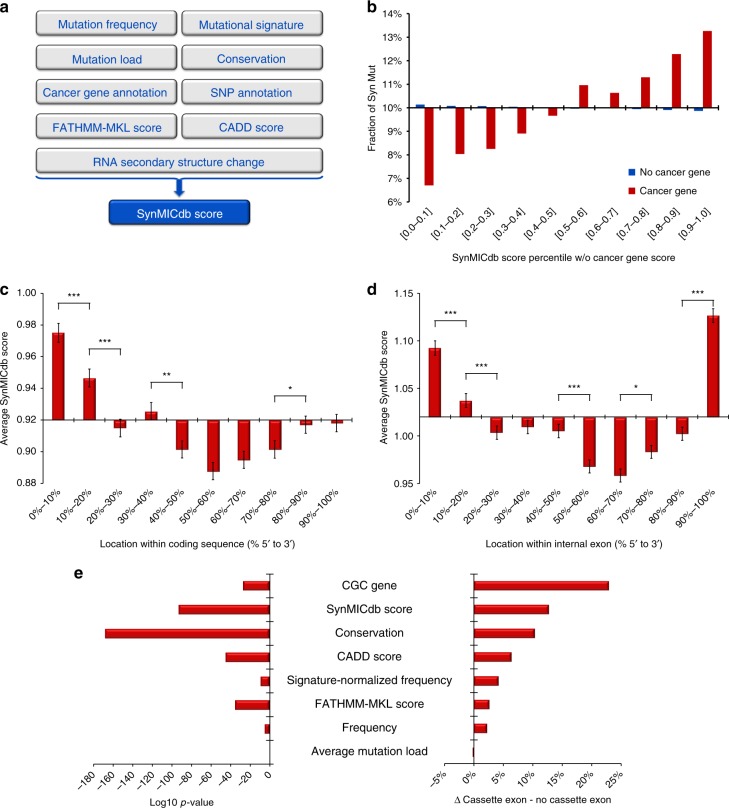
The SynMICdb Score. **a** Nine parameters are integrated into the SynMICdb score for the estimated impact of a synonymous mutation. **b** Synonymous mutations in known cancer genes rank higher in the SynMICdb score than mutations in other genes. Depicted is the fraction of synonymous mutations in cancer genes (red) vs. no cancer genes (blue) in 10% bins of the SynMICdb score ranking (the cancer gene parameter was excluded from the score). A 10% frequency would indicate equal distribution along the 10 bins of the SynMICdb score ranks. Comparing the score rank averages between the group of non-cancer genes and cancer genes revealed a highly significant difference (*t*-test, *p* < 0.001). **c** The average SynMICdb score is depicted along the length of the coding sequence in 10% bins. Significance: *p < 0.05, **p < 0.01, **p < 0.001 (*t*-test). **d** The average SynMICdb score is depicted along the length of the internal exons in 10% bins. Significance: *p < 0.05, **p < 0.001 (*t*-test). **b**–**d** Bars represent the difference from the average (*x*-axis) and the whiskers indicate the standard error (SEM). **e** The SynMICdb score, as well as multiple of its individual parameters are compared for 78,278 synonymous mutations falling into annotated cassette exons vs. all other synonymous mutations with the significance (log10 *p*-value *t*-test, left) and relative difference (% difference of average between both groups, right) depicted

To rank synonymous mutations for their likelihood to have a functional impact, we developed the SynMICdb Score. This score is based on nine different parameters (Fig. 3a): the frequency of the mutation in cancer corrected for mutational bias; the mutation load of the samples affected by this mutation with the rationale that lower mutation loads may indicate a higher specificity of the mutation; the evolutionary conservation; the annotation as a known cancer gene or as a single nucleotide polymorphism (SNP); the FATHMM-MKL and CADD scores^35^; and the predicted impact on RNA secondary structure. Most score parameters were independent of each other (Supplementary Fig. 3a). To characterize the score, we analyzed the score distribution for mutations in known cancer genes (while of course excluding the parameter “Cancer Gene” from the score). Mutations in cancer genes were clearly associated with higher scores compared with mutations in non-cancer genes (Fig. 3b). To analyze the impact of each parameter on the overall score, we used a leave-one-out approach and calculated the score for each mutation with leaving out one of the parameters. The complete SynMICdb score, as well as all leave-one-out scores correlated well with each other (Supplementary Fig. 3b). We then compared the ranking of the top 10% according to the complete SynMICdb score in each of these leave-one-out scores. For all leave-one-out scores, the large majority of the top-ranked mutations remained in the top 10% depicting the balance of the different parameters in the score (Supplementary Fig. 3c). This analysis showed that the signature-normalized frequency, the mutation load and the known cancer gene annotation had the largest impact on the score, which was also desired. On the other hand, all individual factors contributed to the score and altered the ranking.

The distribution of the SynMICdb score allowed ranking synonymous mutations (Supplementary Fig. 3d). The score ranged from −4 to + 12 and high numbers indicated a higher likelihood of a functional impact with the followingdistribution: score > 0.89 = top 50%, score > 2.70 = top 10%, score > 4.38 = top 1%, score > 5.83 = top 0.1%, score > 8.08 = top 0.01%, i.e., a SynMICdb score of above 4.38 indicates that the synonymous mutation is among the top 1% in this study.

Along the coding sequence, the score was significantly higher at the 5'-end (Fig. 3c). Hence, synonymous mutations were less frequent in this region (Fig. 2a) but with a higher predicted impact. The 5'-terminal synonymous mutations had a significantly higher CADD score, conservation and predicted structural impact and a lower mutational burden (Supplementary Fig. 3e).

Within internal exons, the score was significantly higher towards both ends of the exon (Fig. 3d), the regions in which mutations were depleted (Fig. 2b). These outer regions of internal exons showed a significantly higher degree of evolutionary conservation (Supplementary Fig. 3f).

Cassette exons, exons known to be subject to alternative splicing, also showed a significant enrichment of cancer genes, of the SynMICdb score, of the conservation and of the signature-normalized frequency (Fig. 3e). Out of 208 silent mutations recently linked to splicing alterations^36^, we found 46 in SynMICdb (Supplementary Data 8). These had a significantly higher SynMICdb score (average 1.7 vs. 0.9, *p* = 6 × 10^−6^ *t*-test) and decreased mutation load (average 530 vs. 2167, *p* = 1 × 10^−26^ *t*-test) compared with all.

SynMICdb combined data from 333 different studies, but we also analyzed individual studies. We confirmed a relevant study bias regarding the number of synonymous mutations reported per tumor which was likely due to different mapping and annotation protocols. Notably, the SynMICdb score in part controlled for this bias (although this parameter itself was not part of the score) since studies with very high mutation counts resulted on average in lower SynMICdb scores (Supplementary Fig. 4a). When selecting the ten largest studies incorporated into SynMICdb, eight out of ten studies showed the same depletion of synonymous mutations towards the 5'-end of the coding sequence (Supplementary Fig. 4b) as the entire dataset (Fig. 2a). Moreover, all ten out of ten individual studies showed lower rates of synonymous mutations towards both ends of the internal exons (Supplementary Fig. 4c) recapitulating the finding from all synonymous mutations (Fig. 2b). As for the entire dataset (Fig. 3c, d), the SynMICdb scores were increased towards the 5'-end of the coding sequence in seven out of ten studies (Supplementary Fig. 4d), as well as towards both ends of internal exons in all ten or nine out of ten studies, respectively (Supplementary Fig. 4e).

### SynMICdb: a database for synonymous mutations in cancer

**Fig. 4:**
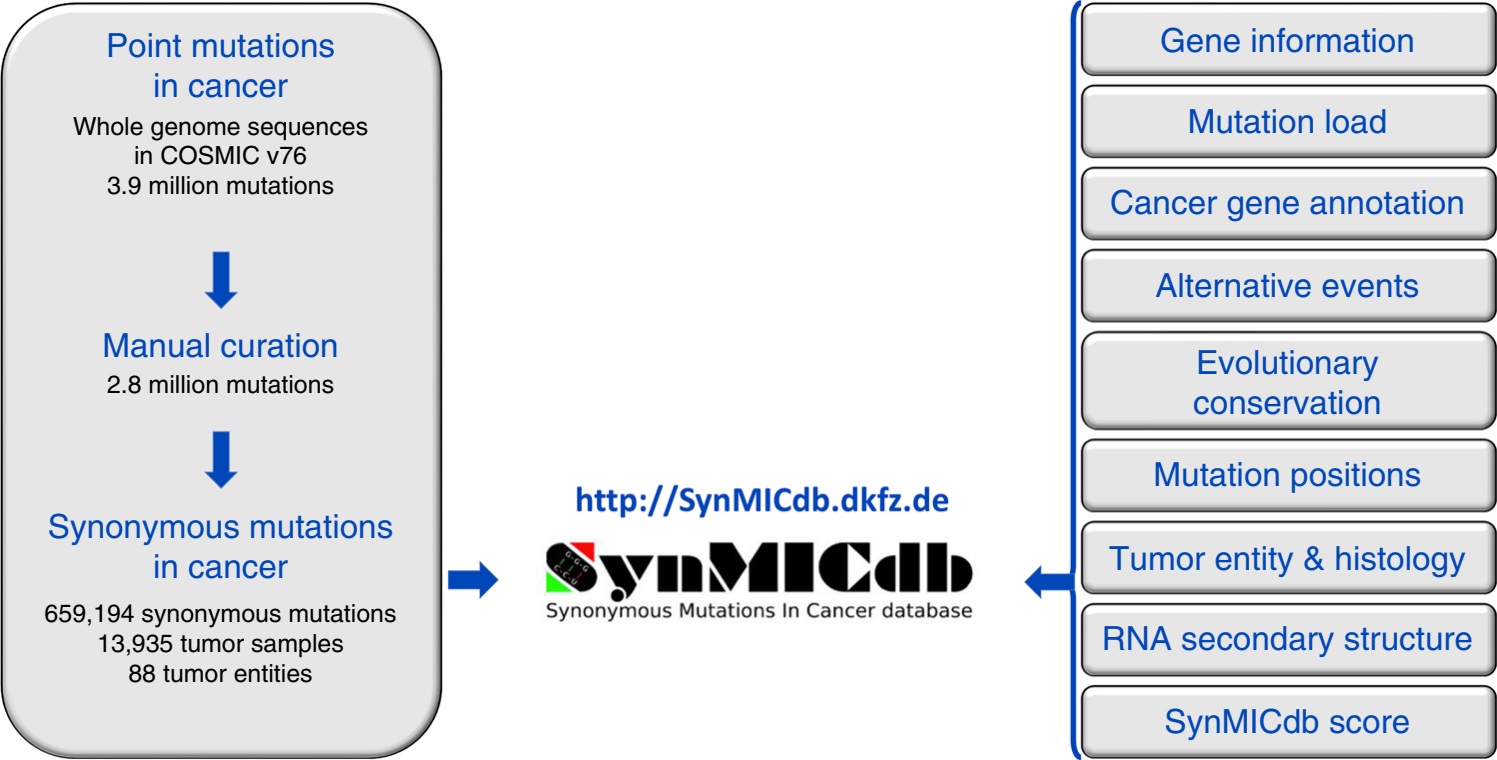
SynMICdb. The Synonymous Mutations in Cancer database provides easy access to 659194 somatic synonymous mutations found in human cancer combined with information about their gene annotation, recurrence, signature-normalized frequency, mutation load, affected tumor entities, evolutionary conservation, structural impact, association with alternative events and the SynMICdb score found at http://SynMICdb.dkfz.de

We made this comprehensive dataset available to the scientific community in a user-friendly database, the SYNonymous Mutations In Cancer database or SynMICdb (http://SynMICdb.dkfz.de) (Fig. 4).

The mutation signature-normalized frequency, evolutionary conservation, SNPs, position in the coding sequence, association with alternative events, predicted impact on secondary structure, average mutation load of the affected samples and SynMICdb score were integrated for each synonymous mutation. Links to gene card information, alias names and information from Cancer Gene Census were added to the database. Alias names or a filter for Cancer Gene Census genes can be used for searching. Researchers can also specifically select mutations based on their localization within the coding sequence. Using Advanced Search, all these features can be searched or sorted for without prior knowledge of bioinformatics or analysis of large datasets. A detailed user guide is provided (Supplementary Note 1).

### RNA structure prediction

Synonymous codon changes have been implicated in altering mRNA structure. Structural changes in the mRNA can affect its stability and translation efficiency^37^. In a long-term evolution experiment in bacteria, mutations which disrupted the mRNA secondary structure were evolutionary negatively selected against^38^. Synonymous mutations in the human *DRD2* gene altered mRNA folding decreasing its stability and translation^23^. Stable mRNA pseudoknot structures cause translational pausing^39^ and regulate translation speed which impacts cotranslational protein folding and interaction with cellular components^40,41^. Comparing mRNA folding energy with protein structures indicates that local mRNA structures influence protein folding^42^.

**Fig. 5:**
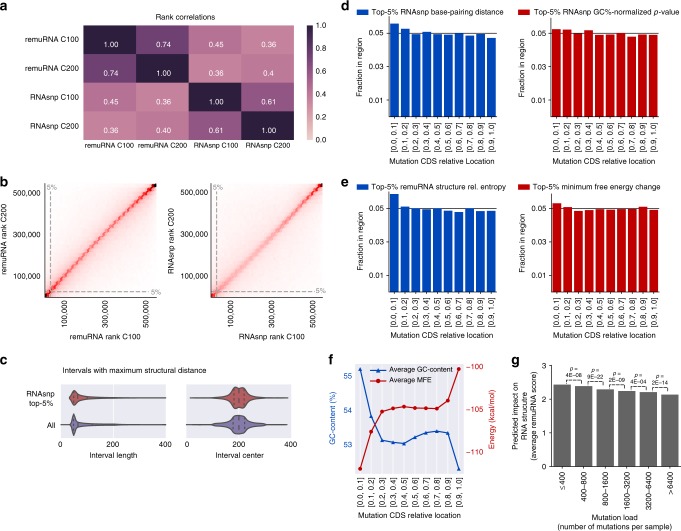
RNA secondary structure analysis of synonymous mutations. **a** Spearman’s rank correlation between the selected scoring metrics remuRNA score (relative entropy of ensembles of structure formations) and RNAsnp (base-pairing distance *p*-value) for two different context lengths C100 (−/+100 nt) and C200 (−/+200 nt). **b** Correlation of the mutation rankings of secondary structure aberrations for the two context lengths C100 or C200 for remuRNA (left) and RNAsnp (right). **c** For each input sequence and mutation, RNAsnp reported the subsequence interval accommodating the largest structural change in terms of maximum base-pair probability distance. For the context length of −/+200 nt around each SNP, the distributions of the interval lengths (left) and the middle positions of the intervals (right) are depicted. **d** Fraction of synonymous mutations with the strongest secondary structure aberrations (top 5th percentile of all synonymous mutations calculated by RNAsnp^44^) within each coding sequence region based on base-pairing distance (left) or *p*-value of base-pairing distance normalized for GC-content (right) are depicted for all synonymous mutations relative to their position in the coding region (10% bins). If mutations would be uniformly distributed along the transcript, the fraction in each region would be 0.05. **e** Fraction of synonymous mutations with the strongest secondary structure aberrations (top 5th percentile of all synonymous mutations calculated by remuRNA) within each coding sequence region based on relative entropy (left) or minimum free energy change (right) are depicted for all synonymous mutations relative to their position in the coding region (10% bins). Again, the expected fraction of a random distribution would be 0.05 for each region. **f** Average GC-content and minimum free energy (MFE) of the RNA secondary structure associated with the context window of 200 nt upstream and downstream of the synonymous mutation are depicted for all synonymous mutations relative to their position in the coding region (10% bins). **g** The predicted impact on the RNA secondary structure (average of remuRNA score) is depicted for different bins of mutation loads with a higher remuRNA score for synonymous mutations found in samples with lower number of mutations in total (significance: *t*-test)

To add another layer of orthogonal data, we used two different algorithms to predict changes in the RNA structure induced by the 659,194 synonymous mutations: remuRNA^43^ (score reflecting relative entropy) and RNAsnp^44^ (*p*-value reflecting significance of local structure base-pairing distance). The resulting rankings of the two structure prediction algorithms were highly correlated for different context sizes (−/+100 nt vs. −/+200 nt) (Fig. 5a). Importantly, the ranking of the predicted structural changes was independent of the window size considered for structure prediction (Fig. 5b), since the interval regions with the largest structural changes were localized in the proximity of the mutation and the majority did not extend beyond the minimum interval size of 50 nt (Fig. 5c).

Comparing the structural impact along the coding sequence revealed an increased fraction of structurally relevant mutations towards the 5'-terminus (Fig. 5d, e). This correlated well with the anticipated higher structuredness of the start codon proximal sites. Hence, while the first codons of the mRNA were depleted in synonymous mutations (Fig. 2a, c), the occurring mutations showed a higher likelihood to alter the structure. The GC-content normalization of the empirical *p*-value calculations balanced this effect, but did not abolish it (Fig. 5d, right panel). Synonymous mutations towards the start of the coding sequence were embedded (−/+100 nt) in a slightly higher GC-content and formed more stable structures with lower free energy (Fig. 5f). This could be partly due to a portion of the 5'-UTR present in the window for mutations close to the 5'-end. However, for mutations after nucleotide 100, the context window was fully located within the coding region and still showed the same trend, which was in accordance with previous experimental studies^45^.

At the nucleotide level, all transversions involving a G showed the largest structural impact while C > T transitions had the smallest impact likely due to the maintained ability to base-pair with G (Supplementary Fig. 5).

Notably, the predicted impact on RNA structure was negatively correlated with mutation load, i.e., synonymous mutations found in samples with overall fewer mutations had a higher impact on RNA structure (Fig. 5g).

### Structural impact of *KRAS* c.30A > C synonymous mutation

Next, we analyzed the dataset of predicted RNA changes for the recurrent synonymous mutations with the most likely structural impact. We selected the mutation *KRAS c.30A* > *C* since its SynMICdb score ranked it in the 99.9th percentile and for the structural impact, RNAsnp ranked it in the 99th percentile and remuRNA in the 87th percentile, hence standing out among the cancer gene-associated synonymous mutations. Notably, this mutation was found in the 5'-terminal region which was generally depleted in synonymous mutations (Fig. 2a, c) but with a higher probability of structure-changing mutations (Fig. 5d, e).

The RAS family of oncogenes consists of *HRAS*, *NRAS,* and *KRAS* in mammals which encode small GTPases^46^. Around 30% of human tumors have mutations in *RAS* genes that lead to constitutive activation of *RAS* due to the inhibition of GTP hydrolysis^47^. *KRAS* is one of the most frequently mutated oncogenes^48^. Mutant KRAS expression promotes oncogenic transformation and downregulation of mutant KRAS leads to tumor regression^49^. Interestingly, mutant KRAS proteins can dimerize with wildtype KRAS, hence, wildtype KRAS expression significantly affects the activity of mutant KRAS and e.g., MEK inhibitor sensitivity^50^.

**Fig. 6:**
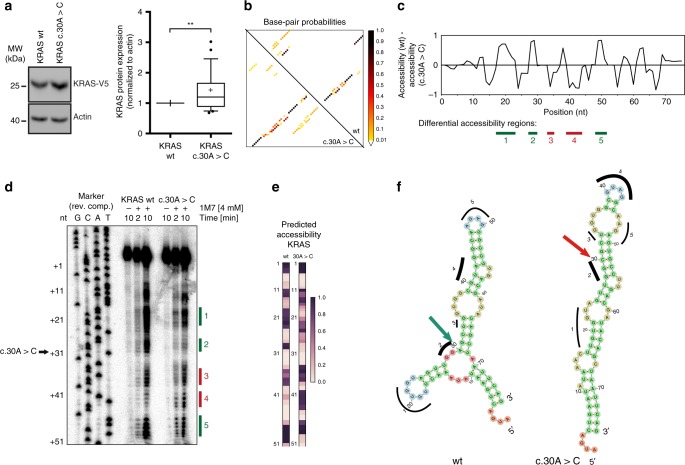
*KRAS c.30 A > C* affects the transcript secondary structure. **a** Left: HEK293 cells were transfected with the indicated KRAS-V5 constructs. Expression of the constructs was evaluated by western blotting using anti-V5 and anti-Actin antibodies. Left: a representative experiment is shown. Right: quantification of the western blot from biological replicates (*n* = 25). V5 signals were normalized to Actin signals presented as boxplot. **p < 0.01 (*t*-test). **b** Base-pairing probabilities of the wildtype and mutant sequences are shown. The mutation introduces a stable rod-like duplex (bottom-left), while the stable structure in the wildtype forms branching stems (top-right). **c** The change in nucleotide accessibility is depicted by the difference between the predicted accessibility along the *KRAS* wildtype vs. mutant transcripts. **d** In vitro SHAPE probing of *KRAS* wildtype (WT) and c.30 A > C mutant *KRAS* using 1M7 shows differential nucleotide accessibility profiles. Lanes 5–7 and lanes 8–10 indicate the SHAPE profile of wildtype *KRAS* and mutant *KRAS* (c.30 A > C), respectively. RNA shown in lanes 5 and 8 are treated with DMSO for 10 min and lanes 6/9 and lanes 7/10 correspond to RNA treated with SHAPE reagent 1M7 (4 mM final concentration) for 2 min and 10 min, respectively. Numbered rectangular boxes correspond to regions of predicted local structural accessibility changes as shown in Fig. 6c. Lanes 1–4 represent the sequencing ladder prepared from *KRAS* DNA as template in complementary sequence. **e** Heatmaps of nucleotide-wise accessibility predicted in silico for comparison with SHAPE. **f** Representation of the secondary structures with minimum free energy by RNAfold for *KRAS* wildtype and mutant RNAs used for SHAPE

First, we tested the impact of the synonymous mutation *c.30* *A* > *C* on exogenous *KRAS* protein expression and found a small, but significant increase (Fig. 6a). The base-pairing probabilities were calculated by the ViennaRNA RNAfold^51^ for the wildtype and c.30 A > C mutant (Fig. 6b, Supplementary Fig. 6). We identified five regions with apparent changes in base-pairing probability: regions 1, 2 and 5 were predicted by RNAplfold to have increased accessibility in the wildtype sequence, while regions 3 and 4 were predicted to show higher accessibility in the mutant sequence (Fig. 6c).

To verify, whether the RNA structure predictions would reflect structural changes, we performed SHAPE (Selective 2'-Hydroxyl Acylation analyzed by Primer Extension) experiments to determine the secondary structure of the wildtype and mutant RNAs^52,53^. The *c.30* *A* > *C* mutation affected secondary structure locally, since the structural changes of a 201 nt context and the 74 nt SHAPE product (see below) were in good agreement. Notably, the accessibility patterns determined by SHAPE reflecting the base-pairing status (Fig. 6d, Supplementary Fig. 7) matched the in silico accessibility predictions (Fig. 6e). We found stronger signals indicating higher accessibility in regions 1, 2 and 5 for the wildtype sequence while the signal in region 4 was much stronger for the mutant sequence. In region 3 with the lowest and shortest predicted difference in accessibility, the difference was less prominent. These SHAPE results were in accordance with two minimum free energy RNA structures for the wildtype and mutant *KRAS* sequences as predicted by RNAfold (Fig. 6f).

In summary, SHAPE unraveled structural differences for *KRAS c.30* *A* > *C* around the mutation site as predicted by RNA structure analysis in silico. Hence, for this example, the structural prediction proved to be useful to identify a synonymous mutation which affected the secondary structure of the mRNA.

### Synonymous mutations of *KRAS* codon 12 affect its expression

**Fig. 7:**
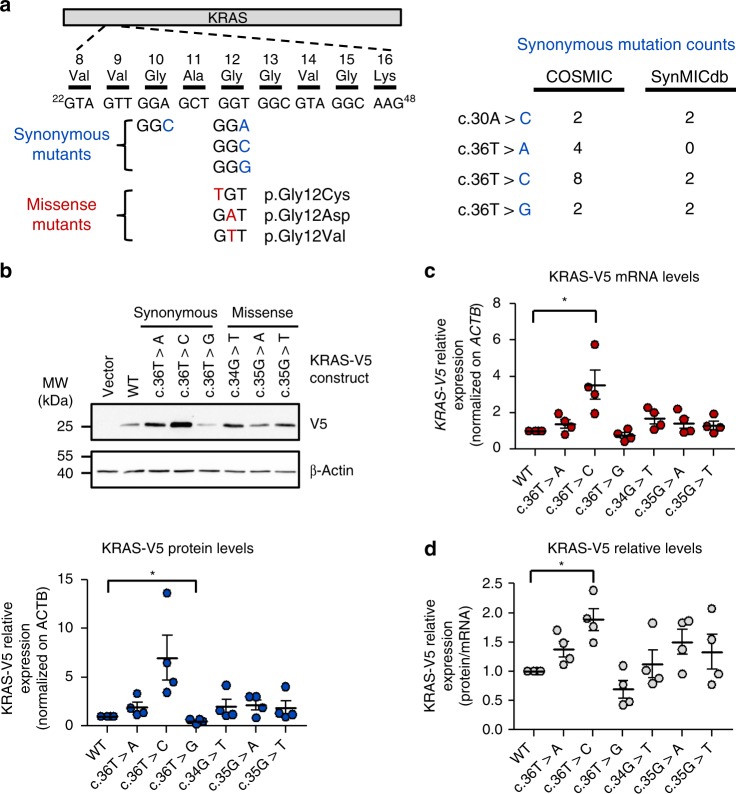
Synonymous mutations in *KRAS* codon 12 impact its expression. **a** Left: Schematic representation of the cancer-derived KRAS mutations analyzed in this study. Right: *KRAS* synonymous mutation counts in COSMIC database v82 and in SynMICdb. **b** HEK293 cells were transfected with the indicated KRAS-V5 mutants. Top: Expression of the constructs was evaluated by Western blotting using V5 and ACTB antibodies. A representative experiment is shown. Bottom: Quantification of the Western blot signals obtained as in top panel. V5 signals were normalized to ACTB signals. **c** Measurement of *KRAS-V5* mRNA levels in the samples described in **b** after RNA isolation and RT-qPCR. *V5* signals were normalized to *ACTB* signals. **d** KRAS-V5 codon 12 mutants relative expression was obtained after normalization of the protein signals for each mutant on their respective mRNA levels. **b**–**d** Mean of four independent experiments is presented. Error bars: SEM. *p ≤ 0.05 (*t*-test)

To determine whether cancer-derived single synonymous mutations may have a detectable impact on the expression of known cancer genes, we again selected the oncogene *KRAS*. Previously, a combination of 130 artificially induced synonymous mutations for the optimization of rare codons had been shown to enhance KRAS expression^54^. In SynMICdb, we identified several recurrent synonymous mutations in *KRAS* clustered at the codons 12 and 13 which are more often affected by oncogenic missense mutations. For codons 12 and 13, we found all three possible mutations of the last nucleotide in different tumor entities with several among the top 0.1% in the SynMICdb score (Fig. 7a). Frequencies are provided for the COSMIC database containing all deposited sequences, as well as for SynMICdb containing only mutations found in whole genome sequencing studies (Fig. 7a). For the large majority of these cases (76%), no second mutation in codons 12 or 13 was detected making the synonymous mutation the only mutation in these codons in the respective tumors. For the remaining samples, it remained uncertain whether additional missense mutations localized to the other allele or whether they affected the same allele and hence would be misannotated. Notably, 86% of the *KRAS* codon 12 mutations in COSMIC (12 out of 14) were found in typically *KRAS*-driven cancers like pancreatic, colon and lung cancer. We tested the impact of these patient-derived *KRAS* mutations on RNA and protein expression in two independent cell lines. Since overactivation of the KRAS pathway increases overall gene expression^55^, we intentionally decided to use a C-terminal V5-tag. C-terminal tags block the membrane association of KRAS and inactivate it allowing us to study the expression but to abolish the activity of the exogenous KRAS. Moreover, this approach allowed specifically detecting the tagged exogenous wildtype or mutant KRAS. Lastly, an N-terminal tag may have masked regulatory events during translation initiation since the mutations were close to the start codon. Several synonymous mutations had a significant effect on KRAS expression with c.36 T > C (G12G, SynMICdb score 99.9th percentile) most strongly inducing KRAS mRNA and protein expression (Fig. 7b–d). This was reproducible in HeLa cells (Supplementary Fig. 8a). In turn, the mutation C.36 T > G (G12G) had the opposite effect and significantly decreased KRAS protein expression (Fig. 7b). Notably, synonymous mutations in codon 13 (G13G) showed the same effect with lowest expression for the GGG codon (Supplementary Fig. 8b–e). Given that increased KRAS activity or the loss of wildtype KRAS as dimerization partner for mutant KRAS proteins could impact oncogenicity^50^, both effects could be of interest for future studies. We also tested missense mutations of these codons and found an effect on gene expression supporting the hypothesis that also missense point mutations may have effects beyond the change of the amino acid. In summary, the example of patient-derived, recurrent somatic synonymous mutations in codon 12 of KRAS documents the potential impact of synonymous mutations on cancer protein expression.

## Discussion

SynMICdb offers a comprehensive, curated, pan-cancer resource to foster research on synonymous mutations. Our analyses provide insights into the characteristics of this second most abundant, but under-researched class of point mutations in cancer. The similarities between synonymous and missense mutations e.g., regarding recurrence or distribution or co-localization with alternative events make it likely that at least some synonymous mutations have a similar impact on tumorigenesis as missense mutations. In turn, missense mutations might share functional mechanisms with synonymous mutations and act beyond the alteration of the amino acid sequence of the encoded protein. Important information on the signature-normalized frequency, mutation load, cancer gene association, evolutionary conservation, predicted structural impact and a sortable SynMICdb score are also provided. The SynMICdb score integrates nine relevant parameters and is enriched for synonymous mutations in cancer genes, as well as for examples recently linked to a function in splicing^36^ or located in cassette exons. In this study, the mutation bias has only been corrected for using mutation signature 1^31^. While this is the most widely applicable signature across tumors and cancer entities and recapitulates the nucleotide change distribution of the synonymous mutation dataset, additional signatures may be relevant in individual tumor entities. Another relevant bias known in comparative cancer genetics is an annotation and reporting bias between different studies, which is evident from differing numbers of synonymous mutations per sample even for the same tumor entity.

Our analyses provide or corroborate a number of arguments in favor of selection and functional relevance of synonymous mutations: synonymous mutations are enriched in known cancer genes, their frequency negatively correlates with the mutation load pointing towards a selective pressure resulting in highly recurrent synonymous mutations, they are non-randomly distributed along the coding sequence and within internal exons and differentially affect codons for specific amino acids.

One of the most surprising findings is the significant depletion of synonymous and missense mutations at the 5'-end of the coding region. This difference is largely independent of the nucleotide change and hence not due to mutational bias. It might be linked to a higher selective pressure against mutations in this region due to a bigger impact of synonymous mutations during the ramping phase of translation initiation or a larger effect of missense mutations on N-terminal signal sequences. If this finding, however, would reflect selective pressure, this would indicate that the majority of the synonymous and missense mutations—even if found only once—would be under selective pressure. It remains an open question how the mutation rate is coupled to the upper end of the coding sequence for such a large number of mutations. Interestingly, the 5'-terminal synonymous mutations showed significantly higher SynMICdb scores—partially due to a higher conservation and predicted structural impact—pointing towards their potential functional relevance as documented for several mutations in the *KRAS* oncogene. The depletion and higher scores towards the 5'-end of the coding sequence have been reproduced in the ten largest individual studies of the dataset.

Synonymous mutations had previously been associated predominantly with splicing regulation^12,17,36^. In our data, multiple findings can be linked to a functional impact on splicing: on the one hand, synonymous mutations are slightly less frequently found in cassette exons than missense mutations and they are depleted towards the boundaries of internal exons (where e.g., exonic splicing silencers would be located^56^). On the other hand, the SynMICdb score is significantly higher for synonymous mutations in cassette exons or close to the ends of the internal exons—partially due to higher conservation of these regions.

These findings of depletion of synonymous mutation towards the ends of internal exons and their higher scores are independent of the nucleotide change and hence not due to mutational bias and have been reproduced in the ten largest individual studies incorporated into the dataset. For the depletion towards the exon ends, our study corroborates a previous observation^57^. Also, the synonymous mutations recently linked to splicing^36^ show significantly increased SynMICdb scores. Lastly, synonymous mutations in cassette exons do not only have a higher SynMICdb score, but are also significantly enriched in cancer genes and have a higher evolutionary conservation and signature-normalized frequency. In the future, all these parameters provided in the SynMICdb could guide the discovery of splicing-regulating synonymous mutations in cancer. Furthermore, we provide the distance to the closest exon-exon boundary and we have searched for the gain or loss of splicing regulatory ESE or ESS motifs due to synonymous mutations (Supplementary Data 4–7). However, the sequence motifs defining ESEs and ESSs are not unanimously characterized (as e.g., indicated by little overlap between motifs from two sources^33,34^ or by assignment of the same sequence motif as ESE and ESS^34^). Other factors like the RNA secondary structure, the distance to the exon boundary or the surrounding sequence will also impact the function of a motif as ESE or ESS^58,59^.

Our studies on structure and expression affected by patient-derived synonymous mutations in the oncogene *KRAS*^46–50^ prove that single point mutations may have a relevant impact on mRNA and protein expression, as well as on the mRNA structure. This structural change results only in a minor expression change in our experimental setting, which may nonetheless be impactful on translation initiation when combined with the endogenous 5'UTR in future studies. A previous study had artificially mutated 130 rare codons of *KRAS* and found an induction of *KRAS* expression by this codon optimization in good accordance with our results on c.36 T > C, while our data is based on a single and patient-derived mutation^54^. Strikingly, these synonymous mutations in *KRAS* frequently occur in the same codons which are known to be affected by missense mutations. Wildtype *KRAS* gene amplification and overexpression have also been described as inducers of cell proliferation^60^, metastasis^61^ and mechanisms of cancer drug resistance to targeted therapeutics^62,63^. Since an increased or decreased wildtype KRAS expression could affect tumorigenesis via increased KRAS activity or via decreased wildtype-mutant dimerization^50^, respectively, synonymous mutations with either effect could be of interest.

SynMICdb holds data from 88 different tumor entities, hence the results represent global patterns and are likely not attributable to only individual tumor entities. In contrast, it cannot be excluded that individual tumor entities may show different patterns.

In summary, our analysis points towards a functional impact of at least a subset of synonymous mutations and offers a comprehensive database enabling researchers without prior knowledge of bioinformatics to find synonymous mutations in their gene or tumor entity of interest and filter them by multiple relevant parameters for functional studies.

## Methods

### Curation of synonymous mutations

The full dataset of mutations listed in the Catalog Of Somatic Mutations In Cancer (COSMIC)^5,6^ v76 derived from whole genome sequencing studies was downloaded comprising 3,881,643 mutations. Only whole genome studies were selected to avoid any biases due to targeted sequencing of individual genes and to allow a quantitative comparison between different genes. The dataset was curated for the erroneous use of the standard genetic code for mitochondrial genes^29^ and non-synonymous mutations misclassified as synonymous mutations (Supplementary Data 2). In COSMIC, the same mutation in the same patient sample can be mapped to multiple transcripts of the same gene introducing a bias when quantitatively comparing genes. Hence, duplicate entries of the same mutation in the same patient sample were removed. This curation yielded 2,812,417 mutations including 659,194 synonymous mutations (Fig. 1a, Supplementary Data 1).

To facilitate the search for organ-specific or tumor type-specific synonymous mutations, the annotated tumors were categorized into three tiers with Organ System-Site-Histology, e.g., Respiratory System-Lung-Adenocarcinoma.

### Analysis of synonymous mutations

Statistics on the dataset were calculated using the Galaxy platform Freiburg^64^ and Excel.

Frequency and recurrence were calculated based on the Mutation ID for synonymous and missense mutations with a recurrent mutation defined as occurring more than once. To calculate the signature-normalized frequency of each mutation, the cancer-associated mutation signature 1^31^ was used, which was the only signature found in all tumor entities and with a high prevalence. The mutation frequency was normalized by multiplying it with (1-probability of the respective nucleotide change), i.e., reducing the frequency for abundant nucleotide changes like C > T and G > A due to the mutation bias in cancer.

The gene names were merged with information from the Cancer Gene Census^30^ v76 to classify the affected genes as related to cancer.

The mutation load, i.e., the mutation burden or the total number of mutations in a given sample, was calculated by counting the total number of mutations listed in COSMIC since all included samples were sequenced genome-wide.

Known single nucleotide polymorphisms (SNPs) were counted as annotated from COSMIC.

For the distribution of nucleotide changes, reciprocal mutations (reverse complementary changes on either genomic strand) were combined into one group resulting in six mutation groups.

For the distribution of amino acids encoded by the affected codons, the number of mutations was calculated for each amino acid and then normalized for the number of codons per amino acid according to the standard genetic code. For the comparison between synonymous and missense mutations, the data was also normalized to the total number of mutations in the respective class. As second, independent approach, the frequency of each codon in the human genome was multiplied with the likelihood for a synonymous mutation based on the mutation bias indicated by mutation signature 1 and compared with the number of synonymous mutations experimentally found in cancer for each amino acid.

For the location of the mutation within the coding region, two different approaches were used: first, the distribution of the mutations relative to the length of the entire coding region was calculated for all genes (Fig. 2a). In addition, only coding sequences with more than 200 codons were selected and then the distribution of the mutations within the first 200 codons was calculated (Supplementary Fig. 2c). To assess these results with regard to a potential mutation bias, this analysis was repeated separately for all six possible types of nucleotide changes (Fig. 2c).

For the location of the mutation within the exons, exon definitions were downloaded from the UCSC Table Browser, GRCh38, All Gencode v24, Comprehensive dataset. To assess these results with regard to a potential mutation bias, this analysis was repeated separately for all six possible types of nucleotide changes (Fig. 2d).

In addition, alternative events for each exon like cassette exons of alternative splicing were merged with our dataset based on the UCSC Table Browser, GRCH38, UCSC Alt Events, knownAlt. The track is described by UCSC as follows:

- Alternate Promoter (altPromoter)-Transcription starts at multiple places. The altPromoter extends from 100 bases before to 50 bases after transcription start.
- Alternate Finish Site (altFinish)-Transcription ends at multiple places.
- Cassette Exon (cassetteExon)-Exon is present in some transcripts but not others. These are found by looking for exons that overlap an intron in the same transcript.
- Retained Intron (retainedIntron)-Introns are spliced out in some transcripts but not others. In some cases, particularly when the intron is near the 3' end, this can reflect an incompletely processed transcript rather than a true alt-splicing event.
- Overlapping Exon (bleedingExon)-Initial or terminal exons overlap in an intron in another transcript. These often are associated with incompletely processed transcripts.
- Alternate 3' End (altThreePrime)-Variations on the 3' end of an intron.
- Alternate 5' End (altFivePrime)-Variations on the 5' end of an intron.
- Intron Ends have AT/AC (atacIntron)-An intron with AT/AC ends rather than the usual GT/AG. These are associated with the minor spliceosome.
- Strange Intron Ends (strangeSplice)-An intron with ends that are not GT/AG, GC/AG, or AT/AC. These are usually artifacts of some sort due to sequencing error or polymorphism.

For the evolutionary conservation, the dataset was merged with the PhastCons scores for 100 vertebrate species derived from the UCSC Table Browser GRCh38.

FATHMM-MKL and CADD scores were extracted using SNPnexus webserver^35^.

We integrated our dataset with the data available from ClinVar, an archive of relationships between human variations and phenotypes with supporting evidence^65^, available through the UCSC Table Browser GRCh38. Matches were only counted if genomic position, as well as nucleotide change were identical. ClinVar mostly contains information about hereditary diseases. To specifically identify cancer-related phenotypes, the entries containing the word “cancer” or “tumor” in the field “phenotypeList” were selected.

For the analysis of splicing regulatory sites, the wildtype sequences were retrieved with 100 nt upstream and 100 nt downstream of the affected nucleotide (or to the boundary of the transcript if shorter). Catalogs of splicing regulatory site motifs (ESE: exonic splicing enhancer, ESS: exonic splicing silencer) were downloaded from two independent sources: RegRNA 2.0^33^ (http://regrna2.mbc.nctu.edu.tw/AEDB/AEdbMotif_all.html) and SpliceAidF^34^ (http://srv00.recas.ba.infn.it/SpliceAidF/). Exonic motifs for the species *Homo sapiens* were selected and duplicates removed. For the motifs listed multiple times in SpliceAidF, the information for each appearance was concatenated (Supplementary Data 4). Notably, 23 motifs were listed as ESE, as well as ESS (marked in red). Then, gains or losses of the 187 motifs were determined comparing the wildtype and synonymous mutant sequences (Supplementary Data 5–7).

To analyze individual studies, the ten largest studies incorporated into the SynMICdb dataset have been selected, i.e., the five studies reporting the largest number of synonymous mutations plus the five studies with the largest numbers of individual samples (study ID & tumor entity: ID540 skin malignant melanoma; ID376 colon adenocarcinoma; ID419 endometrium carcinoma; ID541 stomach adenocarcinoma; ID417 lung adenocarcinoma; ID414 breast carcinoma; ID331 ovary serous carcinoma; ID323 liver hepatocellular carcinoma; ID416 kidney renal clear cell carcinoma; ID328 pancreas ductal adenocarcinoma). Hence, any study bias due to different number of synonymous mutations per sample could be avoided since the ten selected studies included studies with high and low numbers of synonymous mutations per sample.

### SynMICdb score

To rank the compiled list of synonymous mutations, a heuristic score was developed based on the following assumptions: a high frequency of the mutation (normalized for mutation bias), a low mutation load in the affected samples, a mutation in a known cancer gene, a mutation not previously listed as SNP, a high conservation of the affected locus, high FATHMM-MKL and CADD scores^35^ and a high predicted impact on the RNA secondary structure would be positively indicating a higher likelihood of a functional impact of the mutation. All these parameters were included into the SynMICdb score, which was calculated as follows: (log2 (Frequency) + 1) × Signature Normalization factor (1−p))−log10 (average mutation load) + Cancer Gene Census Score ([0;2]) + PhastCons Conservation ([0;1]) + SNP score ([0;1]) + FATHMM-MKL Score ([0;1]) + CADD Score Quantile Rank ([0;1]) + Structural Impact remuRNA Score Quantile Rank ([0;1]) = SynMICdb Score.

The parameters included into the score were largely independent of each other, hence little redundant information was included (Supplementary Fig. 3a). The score was successfully tested for the enrichment of synonymous mutations in cancer genes, as well as on recently published synonymous mutations altering splicing. A leave-one-out analysis documented that the different parameters were balanced in their representation in the score with a desired slightly stronger impact of bias-normalized frequency, mutation load and cancer gene association.

### SynMICdb

SynMICdb has been designed using GNU R/shiny (https://CRAN.R-project.org/package = shiny)^66^. It contains 659,194 synonymous mutations, integrates various of their properties and orthogonal information, as well as provides multiple different search options. A detailed User Guide can be found in the Supplementary Note 1.

### Sequence extraction for RNA structural prediction

Transcript IDs from the database were used to extract sequences. In the first step, transcript versions matching the transcript IDs were extracted from the ENSEMBL annotation of Genome Reference Consortium human genome (GRCh38, ENSEMBL release 83, ENSEMBL annotation file Homo_sapiens.GRCh38.83.gtf) using an implemented Python script. For a small portion of transcripts IDs, no match could be found in the GTF file. For those, gene names from the mutation database were used to retrieve transcript IDs and versions from the GTF file. We then used the UCSC table browser^67^ to generate the exonic genomic intervals from the transcript ID and version in one line per transcript BED12 format (settings for UCSC table browser: *group: Genes and Gene predictions, track: ALL_GENCODE_V24, table: Comprehensive and Pseudogenes*). Afterwards, the associated transcript sequences were extracted from the genome sequence (UCSC whole genome binary file) with UCSC’s *twobittofa* program, passing the transcripts interval file with the default parameters. To retrieve the relative location of mutations on the corresponding transcript sequence and to ensure consistency, a BED6 file with transcript start and the mutation coordinate was intersected with the corresponding transcript genomic interval (BED12 format) for each mutation using the Bedtools Python library^68^ intersect method (version 0.7.9; options “-s –wao -split”), to extract the prefix and its length. From the relative mutation position, 200 bases upstream, as well as downstream of the transcript were extracted (limited to transcript boundaries). These extended mutation sequences were used for the following structure aberration prediction.

### RNA structure aberration prediction

Various methods for predicting RNA secondary structure aberration based on thermodynamic free-energy models exist. The two methods RNAsnp^44^ and remuRNA^43^ have been shown to possess good prediction performance on independent benchmark sets. In brief, RNAsnp computed, for a given sequence and SNV tag, the base-pair probability matrices, for both mutant and wildtype sequences. For evaluating the structure aberration, the matrices are compared using Euclidean distance of base-pairing probabilities. The empirical *p*-value of the mutation effect on RNA structure is computed against a background model of the mutations with similar GC context and length. We computed structure aberrations for all synonymous mutations in the SynMICdb using both RNAsnp (version 1.2) and remuRNA (version 2.0). A workflow with grid computing support was applied to efficiently parallelize the computations in a reasonable time. Utilizing the workflow on our cluster, computation and analysis of around 550,000 individual mutations was completed in about three days.

For Fig. 5a, b, remuRNA relative entropies and RNAsnp empirical *p*-values normalized to GC-content and length were calculated for −/+ 100 or 200 nt windows. Spearman’s rank correlations were calculated by pandas Python library. For Fig. 5c, interval ranges were collected from the first interval column of RNAsnp results and plotted in Python. For Fig. 5d, e, f, sequences that could not be fully extended (length less than 201 nt) were discarded to avoid length biases. For Fig. 5d, base-pairing Euclidean distance and associated GC-normalized *p*-value were obtained from RNAsnp (columns d_max and *p*_value). For Fig. 5e, f, relative entropy, minimum free energies and GC-content were obtained from remuRNA results. For both panels, mutations were ranked separately by these measures to obtain the 5th percentile of mutations with the strongest impact on RNA structure. For each binned CDS region, the fraction was computed by dividing the number of mutations in the 5th percentile to the total number of the synonymous mutations located in that region. For Fig. 5f, minimum free energies and GC-contents were averaged for each bin along the coding sequence.

### In silico structure prediction and visualization

For the selected KRAS c.30 A > C mutation, sequences were folded using the Vienna package (version 2.3.3) with partition function mode enabled for base-pair probability calculations of each. Base-pair probabilities and accessibilities were extracted from Vienna RNAfold and RNAplfold dotplots and accessibility outputs (parameter -u = 1, W = seq-length)^51^. The heatmap vertical scale (Fig. 6e) was manually adjusted to roughly match the corresponding non-linear scale of the SHAPE profiles. For visualizing accessibilities and base-pair probabilities, Circos^69^ and custom Python scripts were used. Associated bioinformatics scripts and reproducible interactive Notebooks are available under the GitHub repository https://github.com/BackofenLab/MutARNA/. The minimum free energy structures were computed using RNAfold and drawn with forna^70^.

### In vitro chemical probing of RNA secondary structure using SHAPE

The Selective 2´-Hydroxyl Acylation analyzed by Primer Extension (SHAPE) protocol was used including chemical modification of RNA by 1-methyl-7-nitroisatoic anhydride (1M7)^52,53^, converting and labeling the RNA by reverse transcription and running the cDNA on a sequencing gel.

To analyze the structure of *KRAS* transcripts extending by SHAPE, we first constructed a cassette in a pCRII-TOPO vector within two *EcoRI* cleavage sites which consisted of the T7 promoter (underlined) followed by the *KRAS* transcript from the nucleotide position 1 until position 75 (either wildtype or synonymous *KRAS* mutant c.30 A > C) and a subsequent RT primer binding site (italicized) for probing the structure with a universal primer.

KRAS WT:

(5'GAATTCGCCCTTTAATACGACTCACTATAGGGATGACTGAATATAAACTTGTGGTAGTTGGAGCTGGTGGCGTAGGCAAGAGTGCCTTGACGATACAGCTAATTCAGAAAT*CGGGCTTCGGTCCGGTTC*AAGGGCGAATTC3')

KRAS c.30 A > C:

(5'GAATTCGCCCTTTAATACGACTCACTATAGGGATGACTGAATATAAACTTGTGGTAGTTGGCGCTGGTGGCGTAGGCAAGAGTGCCTTGACGATACAGCTAATTCAGAAAT*CGGGCTTCGGTCCGGTTC*AAGGGCGAATTC3')

The plasmids containing the *KRAS* fragments were digested using *EcoRI* and the digested product was used as a template for *in vitro* transcription overnight according to the manufacturer's recommendation with the MEGAscript T7 Transcription Kit (Life Technologies). The transcribed RNA was further extracted using the phenol-chloroform method.

For chemical modification of the RNA, 8 µg of *KRAS* transcript in 4 µl RNase-free water was denatured at 95 °C for 3 min and snap-cooled on ice for 1 min. 36 µl of 1.1× folding buffer (1.1 mM MgCl_2_, 111 mM NaCl and 111 mM HEPES, pH 8.0) was added before incubating at 37 °C for 20 min. From the resulting 40 µl, three aliquots of 10 µl each were treated either with DMSO at room temperature for 10 min or with 1M7 (4 mM final concentration) and incubated at room temperature for 2 min or 10 min. Under single-hit kinetics, a single modification per transcript will occur by the SHAPE reagent, which preferentially modifies the ribose 2'-hydroxyl groups of unpaired, conformationally flexible nucleotides. Chemically modified *KRAS* transcripts were then ethanol precipitated and dissolved in 10 µl RNase-free water.

One hundred nanogram of the chemically modified RNA was reverse transcribed as per the manufacturer’s protocol (SuperScript® IV Reverse Transcriptase, Thermo Scientific) using the SHAPE RT primer (5'-GAACCGGACCGAAGCCCG-3') 5´-end labeled using the T4 polynucleotide kinase according to the manufacturer's protocol (T4 Polynucleotide Kinase, New England Biolabs) and γ-[^32^P]ATP. Reverse transcription is blocked by the chemical modification by 1M7 leading to a pool of cDNAs whose length distribution on the gel is an indicator of the distribution of modifications across the RNA. The size marker for the sequencing gel was prepared using the *KRAS* DNA as template and [^32^P]-labeled SHAPE RT Primer in the presence of ddNTPs (USB® Thermo Sequenase Cycle Sequencing Kit, Thermo Scientific). This led to the generation of fragments terminated at the site of ddNTP incorporation, which was reflecting the sequence of the fragment. cDNAs are then resolved according to length on a 7.5 M Urea-PAGE sequencing gel (7.5%). The gel was heat-dried under vacuum and visualized using a phosphorimager. All probing experiments were performed in triplicates.

### Plasmid construction for expression analysis

KRAS expression constructs were generated using the Gateway recombination cloning technology (Thermo Scientific). Briefly, an entry clone consisting of the human KRAS 4B coding sequence without a stop codon (a generous gift of Dr. Christopher Oakes, DKFZ Heidelberg) was used as a template for PCR-based site-directed mutagenesis (KRAS G34T For: 5'-GAGCTTGTGGCGTAGGCAAGA-3', KRAS G34T Rev: 5'-GCCACAAGCTCCAACTACCAC-3', KRAS G35A For: 5'-AGCTGATGGCGTAGGCAAGAG-3', KRAS G35A Rev: 5'-CGCCATCAGCTCCAACTACCA-3', KRAS G35T For: 5'-AGCTGTTGGCGTAGGCAAGAG-3', KRAS G35T Rev: 5'-CGCCAACAGCTCCAACTACCA-3', KRAS T36A For: 5'-GCTGGAGGCGTAGGCAAGAGT-3', KRAS T36A Rev: 5'-ACGCCTCCAGCTCCAACTACC-3', KRAS T36C For: 5'-GCTGGCGGCGTAGGCAAGAGT-3', KRAS T36C Rev: 5'-ACGCCGCCAGCTCCAACTACC-3', KRAS T36G For: 5'-GCTGGGGGCGTAGGCAAGAGT-3', KRAS T36G Rev: 5'-ACGCCCCCAGCTCCAACTACC-3', KRAS G37T For: 5'-CTGGTTGCGTAGGCAAGAG-3', KRAS G37T Rev: 5'-TACGCAACCAGCTCCAACT-3', KRAS G38A For: 5'-TGGTGACGTAGGCAAGAGT -3', KRAS G38A Rev: 5'-CTACGTCACCAGCTCCAAC-3', KRAS C39A For: 5'-GGTGGAGTAGGCAAGAGTGC-3', KRAS C39A Rev: 5'-CCTACTCCACCAGCTCCAA-3', KRAS C39G For: 5'-GGTGGGGTAGGCAAGAGTGC-3', KRAS C39G Rev: 5'-CCTACCCCACCAGCTCCAA-3', KRAS C39T For: 5'-GGTGGTGTAGGCAAGAGTGC-3', KRAS C39T Rev: 5'-CCTACACCACCAGCTCCAA-3'). Mutation of the entry clones was confirmed by DNA sequencing. Then, wild-type or mutant KRAS entry clones were recombined into the pEF-DEST51 Gateway expression vector (Thermo Scientific) using the LR Clonase II enzyme mix (Thermo Scientific) according to the manufacturer’s instructions. To generate the empty vector control, the pEF-DEST51 expression vector was PCR-amplified to delete the chloroamphenicol resistance and the ccdB genes while adding a Kozak sequence followed by a start codon in frame with the V5-6xHis tag. The following primers were used: Forward 5‘-GCCACCATGGCTTTCTTGTACAAAGTGGT-3‘ and Reverse 5‘-AGCTTTTTTGTACAAACTTGTTG-3‘. The PCR reaction was *DpnI*-digested for 30 min at 37 °C and the PCR product was purified on a gel, before being ligated.

### Cell culture and transfections

HEK293 cells (identity confirmed via fingerprinting by Multiplexion, Friedrichshafen, Germany; matching ATCC CRL-1573) were cultured in Dulbecco’s modified Eagle’s medium (DMEM, Sigma-Aldrich) supplemented with 10% fetal bovine serum (FBS, Thermo Scientific) and 2 mM L-glutamine (Sigma-Aldrich). Cells were maintained at 37 °C under a 5% CO_2_ atmosphere. For transient expression, cells were transfected with TransIT-LT1 (Mirus Bio LLC) according to the manufacturer's instructions. 60 h post transfection, cells were washed twice and harvested in ice-cold PBS. Each cell sample was split into two and further processed for protein and mRNA levels analysis.

### Western blot analysis

For protein level analysis, cell extracts were prepared in lysis buffer (25 mM Tris-Cl pH 7.4, 150 mM NaCl, 5 mM EDTA, 1 mM DTT, 1% Triton X-100 and complete EDTA-free protease inhibitor cocktail [Roche Applied Science]). Cell lysates were centrifuged at 16,000 × *g* for 15 min and the protein concentrations of the cleared samples were determined with a BCA assay. Cell extracts (10 μg) were separated via SDS–PAGE and transferred to nitrocellulose membranes (GE Healthcare). Protein expression was analyzed by western blotting using the following primary antibodies: β-actin (A2228, Sigma-Aldrich, 1:20000) and anti-V5 (1:1000-1:2500, R96025, Thermo Scientific). The Western blot signals were acquired with an Intas ECL ChemoCam Imager (Intas) and quantified using the LabImage 1D software (Kapelan Bio-imaging). Expression values were compared by unpaired two-sided *T*-tests after scedasticity was determined using the *F*-test.

### RNA extraction, reverse transcription and qPCR

For steady-state RNA levels quantification, cells were lysed in TRI Reagent (Sigma-Aldrich) according to the manufacturer's instructions and samples were subsequently digested with DNase I (Roche) for 30 min at 37 °C. Reverse transcription reactions were performed with 1 µg of DNase-treated RNA using the RevertAid Reverse Transcriptase (Thermo Scientific) and random hexamer primers (Thermo Scientific). cDNAs were amplified by real-time quantitative PCR using 2x Power SybrGreen Master Mix (Applied Biosystems) and the following specific primer pairs: β-Actin Forward: 5’-TCAAGATCATTGCTCCTCCTGAG-3’, β-Actin Reverse: 5’-ACATCTGCTGGAAGGTGGAC-3’, pEF-DEST51 Forward: 5’-CGCCAGAACACAGGTGTCG-3’, pEF-DEST51 Reverse: 5’-TTGTTGATCAAGCTTACCTAGCC-3’. Reactions were run in technical triplicates in an Applied Biosystems StepOnePlus cycler using the following program: 10 min incubation at 95 °C prior to 40 cycles of 15 s at 95 °C and 30 s at 60 °C. Relative expression of target genes was determined by the 2-ΔΔCt method using average Ct values. Experiments were performed in three biological replicates. Expression values were compared by unpaired two-sided *T*-tests after scedasticity had been determined using the *F*-test.

### Reporting summary

Further information on research design is available in the Nature Research Reporting Summary linked to this article.

## Supplementary information

Supplementary Information

Description of Additional Supplementary Files

Supplementary Data 1

Supplementary Data 2

Supplementary Data 3

Supplementary Data 4

Supplementary Data 5

Supplementary Data 6

Supplementary Data 7

Supplementary Data 8

Reporting Summary

Source Data

## Data Availability

Datasets referenced during the study are available from COSMIC, Biomart and UCSC [http://www.genome.ucsc.edu/cgi-bin/hgTables] websites. All the other data supporting the findings of this study are available within the article, its supplementary information files, the SynMICdb database [http://SynMICdb.dkfz.de] and from the corresponding author upon reasonable request. The source data underlying Figs. 6a, 7b, c and Supplementary Fig. 8a, 8c and 8d are provided as a Source Data File. The replicates for Fig. 6d are provided in Supplementary Fig. 7. The SynMICdb can be found at http://SynMICdb.dkfz.de.
