# Supplementary material for: A pan-cancer analysis of synonymous mutations
Source: Nat Commun. 2019 Jun 12;10:2569. doi: 10.1038/s41467-019-10489-2 (PMC6562042; doi:10.1038/s41467-019-10489-2)
Supplement: Supplementary file 1 — Supplementary Information [file 41467_2019_10489_MOESM1_ESM.pdf]

A pan-cancer analysis of synonymous mutations

Yogita Sharma[‡ 1,2,3], Milad Miladi[‡ 6], Sandeep Dukare[‡ 4,5], Karine Boulay[‡ 4], Maiwen Caudron-Herger[4], Matthias Groß[4], Rolf Backofen[6], Sven Diederichs* [1-5]

Supplementary Information

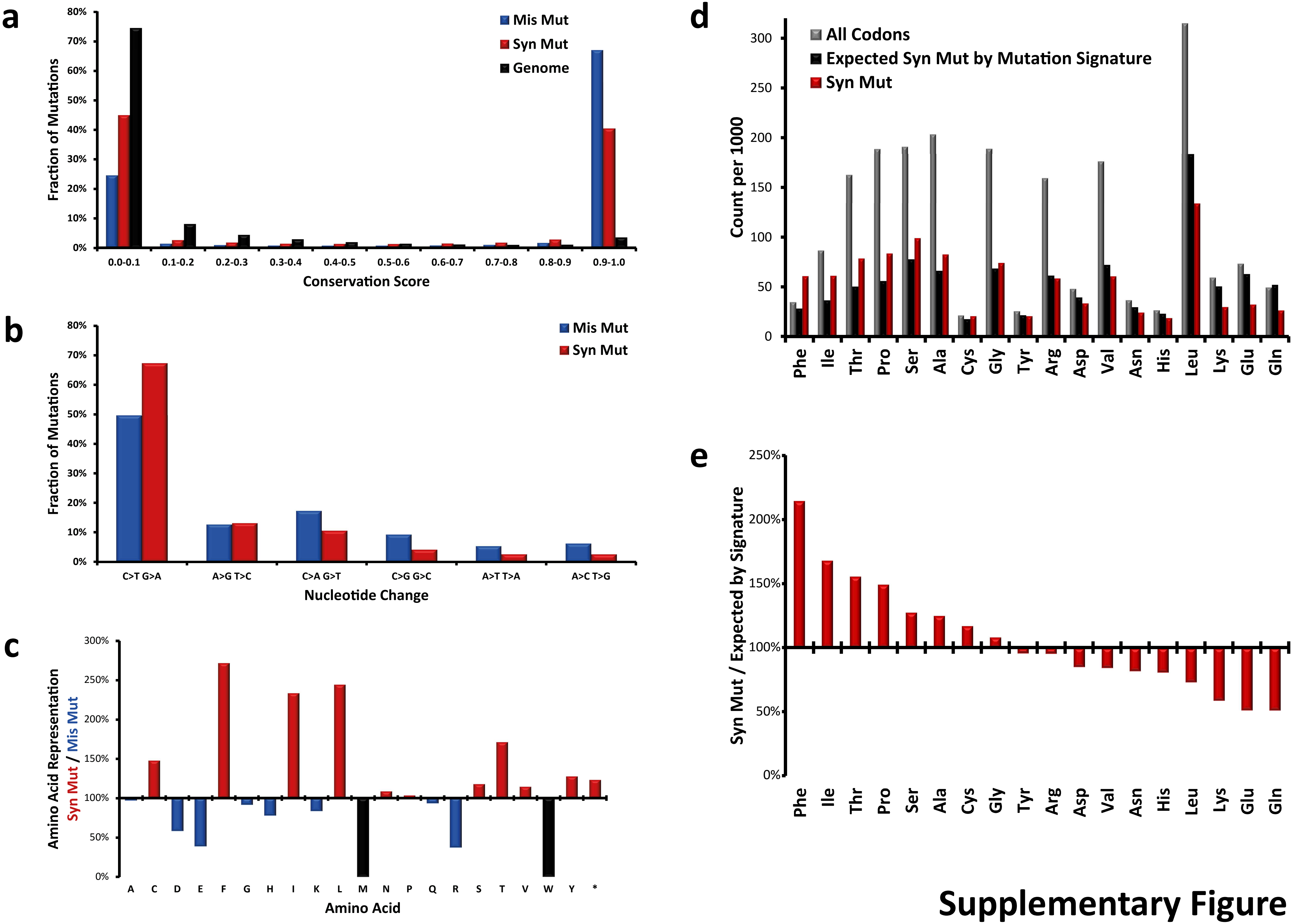

Supplementary Figure 1

Supplementary Figure 1: Conservation, nucleotide and amino acid changes of synonymous and missense mutations

(a) The distribution of PhastCons conservation scores among 100 vertebrate species are depicted for synonymous mutations (Syn Mut), missense mutations (Mis Mut) and the human genome. **(b)** Nucleotide changes underlying synonymous mutations (Syn Mut) or missense mutations (Mis Mut) are depicted with C>T / G>A changes being the far most abundant change in both groups. **(c)** The distribution of the amino acids affected by synonymous mutations (Syn Mut) or missense mutations (Mis Mut) were analyzed. The results were normalized for the number of codons per amino acid in the genetic code (amino acids with more codons could be more likely affected by synonymous mutations) and normalized for the total number of synonymous vs. missense mutations. The ratio between synonymous and missense mutations is depicted. Values above 100% (red) indicate an enrichment for synonymous mutations e.g. for the hydrophobic amino acids Phe (F), Leu (L) and Ile (I). Values below 100% (blue) indicate an enrichment for missense mutations compared to synonymous mutations e.g. for the charged amino acids Arg (R), Glu (E) and Asp (D). The amino acids Met (M) and Trp (W) are only encoded by one codon and hence cannot have any synonymous mutations (black). **(d)** The distribution of codons in the human transcriptome, the number of synonymous mutations expected by the mutation bias in cancer and the actual number of synonymous mutations in cancer are compared for each amino acid. **(e)** The ratio between the determined and the expected number of synonymous mutations is depicted.

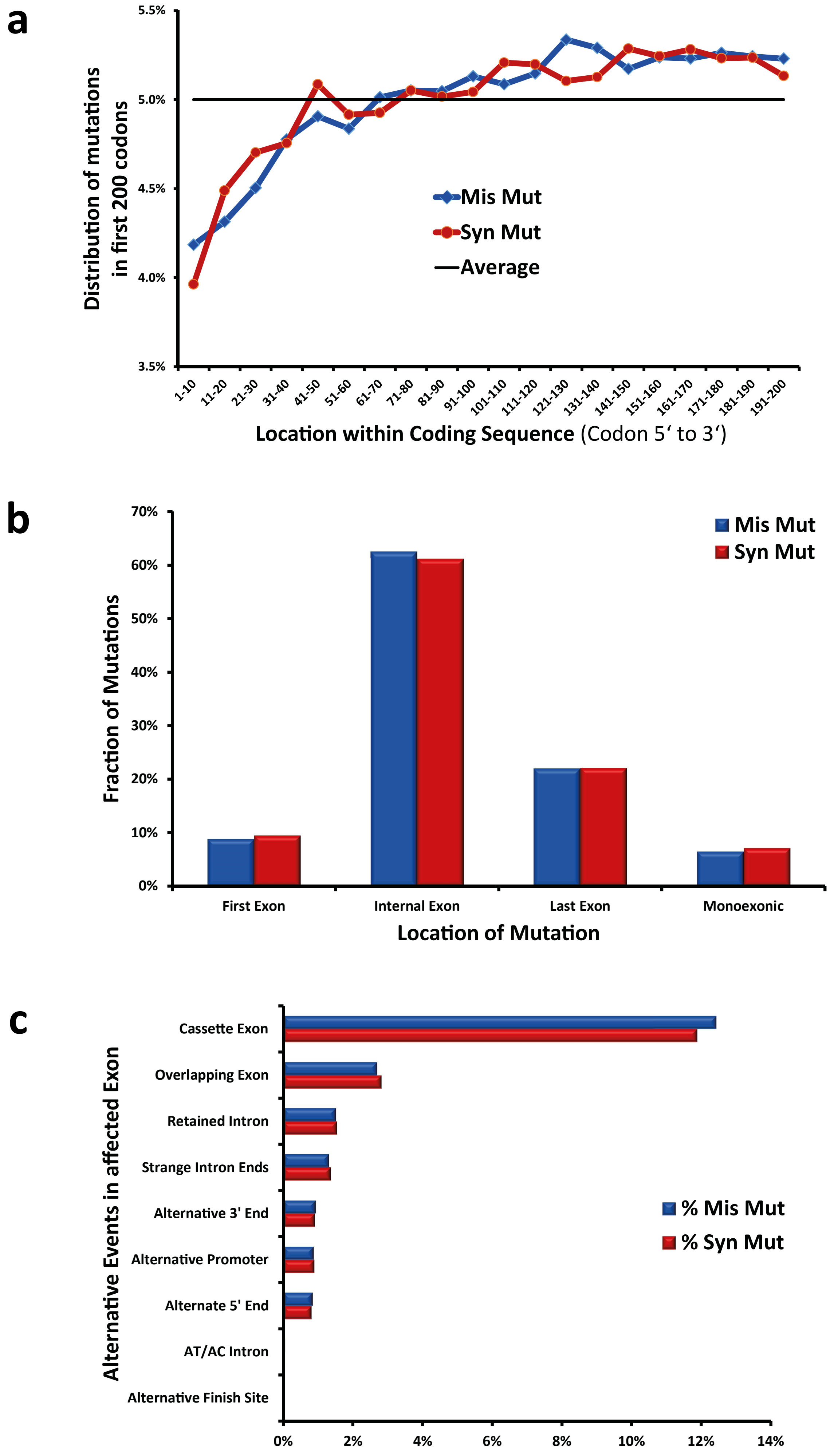

Supplementary Figure 2

Supplementary Figure 2: Location of synonymous and missense mutations within the gene

(a) The distribution of mutations in the first 200 codons of proteins with more than 200 codons is depicted. The black line at 5% frequency would indicate equal distribution along the 20 bins of the first 200 codons. Notably, synonymous mutations (Syn Mut) as well as missense mutations (Mis Mut) are depleted towards the 5'-end of the coding region. **(b)** The distribution of synonymous mutations (Syn Mut) and missense mutations (Mis Mut) between the first, internal or the last exon of multiexonic transcripts as well as in monoexonic transcripts is depicted. **(c)** Mutations were mapped to their respective exons and these exons were analyzed for alternative events (UCSC). Notably, synonymous mutations (Syn Mut) and missense mutations (Mis Mut) were similarly distributed, e.g. equally frequent in cassette exons subject to alternative splicing.

a

Correlation Score Parameters	Structural Prediction	CADD Score	Cancer Genes	SNP Information	FATHMM-MKL Score	Frequency	Conservation	Mutation Load
Structural Prediction	1.00	-0.02	0.00	0.01	0.00	-0.04	0.01	-0.04
CADD Score	-0.02	1.00	0.02	0.07	0.27	-0.01	0.15	0.06
Cancer Genes	0.00	0.02	1.00	0.00	0.04	0.01	0.04	-0.01
SNP Information	0.01	0.07	0.00	1.00	0.07	-0.14	0.06	-0.03
FATHMM-MKL Score	0.00	0.27	0.04	0.07	1.00	-0.09	0.71	0.02
Frequency	-0.04	-0.01	0.01	-0.14	-0.09	1.00	-0.10	-0.07
Conservation	0.01	0.15	0.04	0.06	0.71	-0.10	1.00	0.02
Mutation Load	-0.04	0.06	-0.01	-0.03	0.02	-0.07	0.02	1.00
				Color Legend:		-1.00	0.00	1.00

b

Correlation Scores leave-one-out	Score all	Score w/o Structural Prediction	Score w/o CADD Score	Score w/o Cancer Genes	Score w/o SNP Information	Score w/o FATHMM-MKL Score	Score w/o Frequency * Signature	Score w/o Conservation	Score w/o Mutation Load
Score all	**1.00**	**0.98**	**0.98**	**0.97**	**0.97**	**0.96**	**0.94**	**0.93**	**0.84**
Score w/o Structural Prediction	**0.98**	1.00	0.95	0.94	0.94	0.93	0.92	0.89	0.81
Score w/o CADD Score	**0.98**	0.95	1.00	0.94	0.94	0.95	0.89	0.91	0.79
Score w/o Cancer Genes	**0.97**	0.94	0.94	1.00	0.93	0.93	0.90	0.89	0.80
Score w/o SNP Information	**0.97**	0.94	0.94	0.93	1.00	0.92	0.89	0.89	0.80
Score w/o FATHMM-MKL Score	**0.96**	0.93	0.95	0.93	0.92	1.00	0.87	0.96	0.73
Score w/o Frequency * Signature	**0.94**	0.92	0.89	0.90	0.89	0.87	1.00	0.84	0.79
Score w/o Conservation	**0.93**	0.89	0.91	0.89	0.89	0.96	0.84	1.00	0.68
Score w/o Mutation Load	**0.84**	0.81	0.79	0.80	0.80	0.73	0.79	0.68	1.00
					Color Legend:		-1.00	0.00	1.00

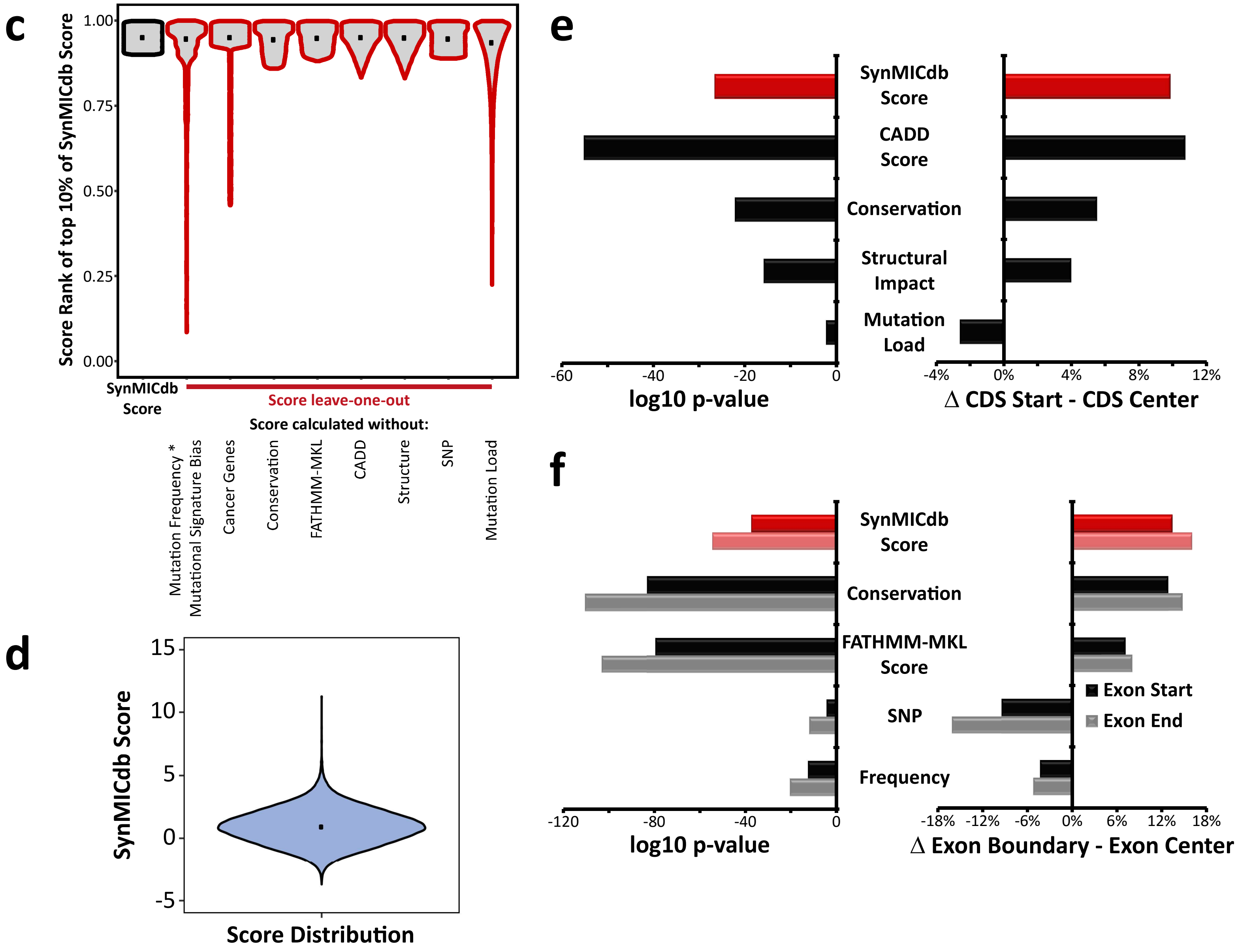

Supplementary Figure 3

Supplementary Figure 3: Characterization of the SynMICdb Score

(a) This table lists the correlation coefficients of the individual score parameters across all synonymous mutations. **(b)** This table provides the correlation coefficients for the SynMICdb score as well as for all leave-one-out scores across all synonymous mutations. **(c)** The violin plot indicates the ranking of the top 10% of the synonymous mutations by SynMICdb score for all leave-one-out scores, i.e. for the scores calculated with one parameter of the score lacking. **(d)** The distribution of the SynMICdb score is depicted in a violin plot. The median score is 0.89 and the top 10% of synonymous mutations have a score above 2.70. **(e)** Comparison of the first decile (0%-10%) of the coding sequence (CDS) to the central region of the CDS (50%-60%). Depicted is the difference (in %) and statistical significance (t-test) of the difference between these two deciles. The SynMICdb score is higher in the first decile as well as its parameters CADD score, evolutionary conservation and structural impact, while the patients with mutations in the first decile of the CDS have a lower mutation load. **(f)** Comparison of the first and last decile (0%-10% darker bars / 90%-100% lighter bars) across the internal exon length to the central region of the internal exon with the highest count of synonymous mutations (50%-60%). Depicted is the difference (in %) and statistical significance (t-test) of the difference between the two respective deciles. The SynMICdb score is higher in the first and last decile compared to the central decile as well as its parameters evolutionary conservation and FATHMM-MKL score, while the density of SNPs in the synonymous mutations at the exon boundaries is decreased (also increasing the SynMICdb score). In contrast, the recurrence (frequency) of the individual synonymous mutations is lower in the outer deciles contributing a negative impact on the SynMICdb score.

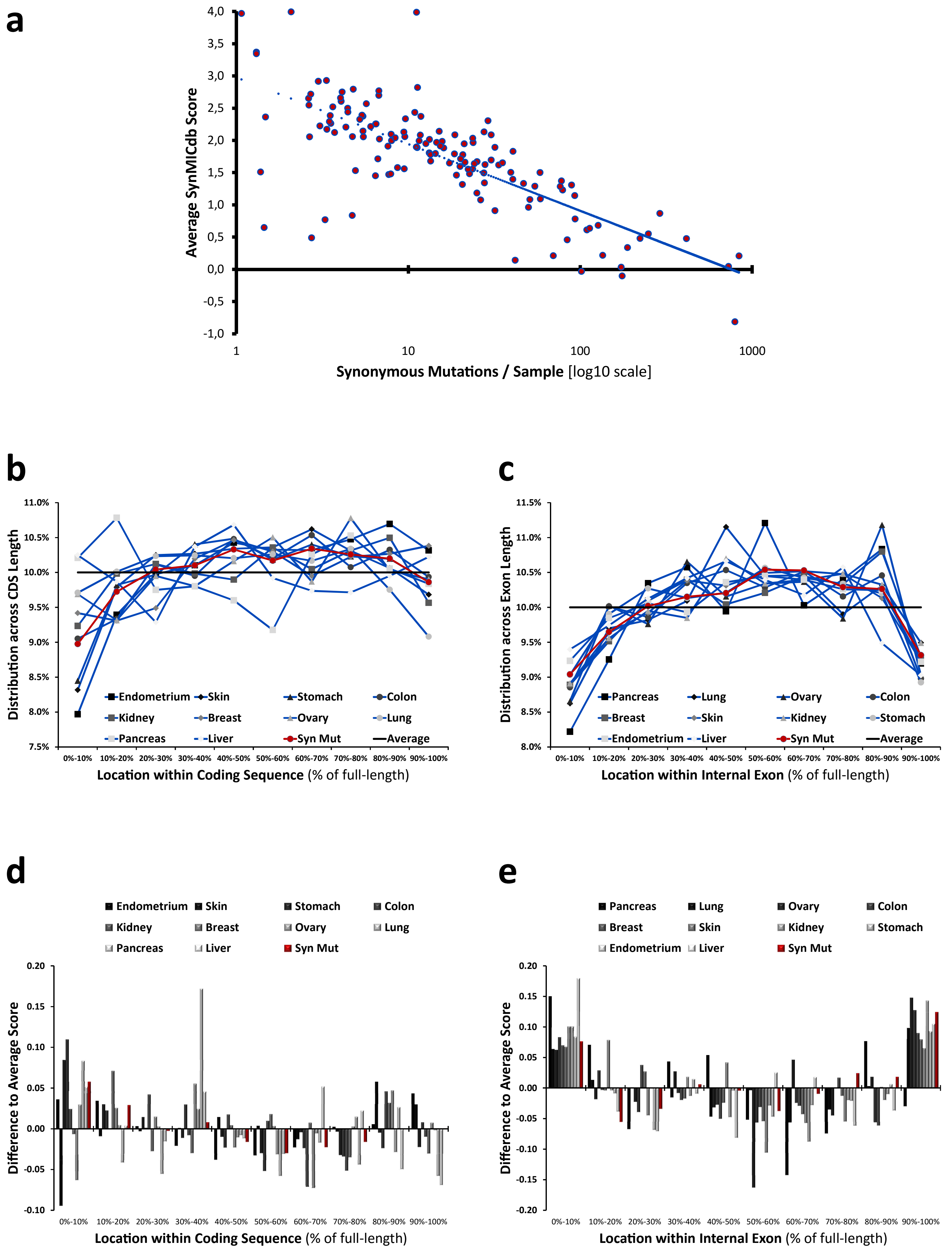

Supplementary Figure 4

Supplementary Figure 4: Analysis of individual studies

(a) Comparison of the number of synonymous mutations per sample in 333 studies aggregated in SynMICdb unravels a study bias which is partially corrected for by the SynMICdb score - synonymous mutations found in studies with very high counts for synonymous mutations per sample show lower scores. **(b)** The distribution of the positions within the coding region of all mutations in all affected genes independent of their length is depicted in 5'-to-3' direction for the ten largest individual studies in SynMICdb. The black line at 10% frequency would indicate equal distribution along the 10 bins of the coding sequence length. Synonymous mutations are depleted towards the 5'-end of the coding region in 8 out of 10 studies. The complete set of synonymous mutations is depicted for comparison (Syn Mut, red). **(c)** The distribution of mutations within internal exons of multiexonic transcripts is depicted in 5'-to-3' direction for the ten largest individual studies in SynMICdb. The black line at 10% frequency would indicate equal distribution along the 10 bins along the internal exon length. Synonymous mutations are depleted towards both ends of the exon in all ten studies. The complete set of synonymous mutations is depicted for comparison (Syn Mut, red). **(d)** The difference to the average SynMICdb score is depicted along the length of the coding sequence in 10% bins for the ten largest individual studies in SynMICdb. The SynMICdb score is increased in the 5'-terminal synonymous mutations in seven out of ten individual studies. The complete set of synonymous mutations is depicted for comparison (Syn Mut, red). **(e)** The difference to the average SynMICdb score is depicted along the length of the internal exons in 10% bins for the ten largest individual studies in SynMICdb. The SynMICdb score is increased in the synonymous mutations towards the 5'-end of the internal exons in all ten individual studies and towards the 3'-end of the internal exons in nine out of ten individual studies. The complete set of synonymous mutations is depicted for comparison (Syn Mut, red).

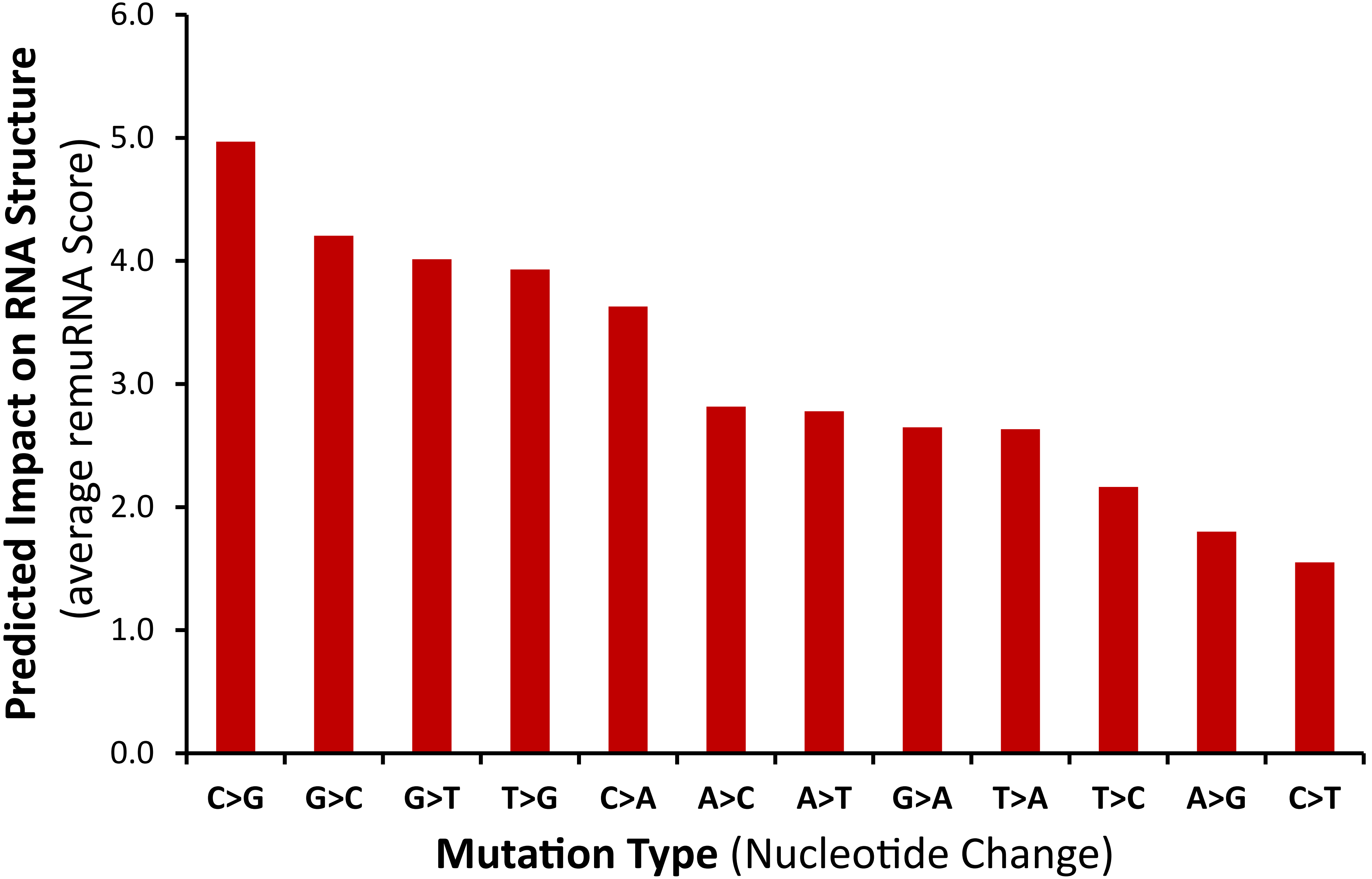

Supplementary Figure 5

Supplementary Figure 5: Impact of different nucleotide changes on RNA structure

The average predicted impact on the RNA secondary structure (remuRNA score) of the 12 different nucleotide changes is depicted.

a KRAS wt

b KRAS c.30A>C

c Difference KRAS wt vs. c.30A>C

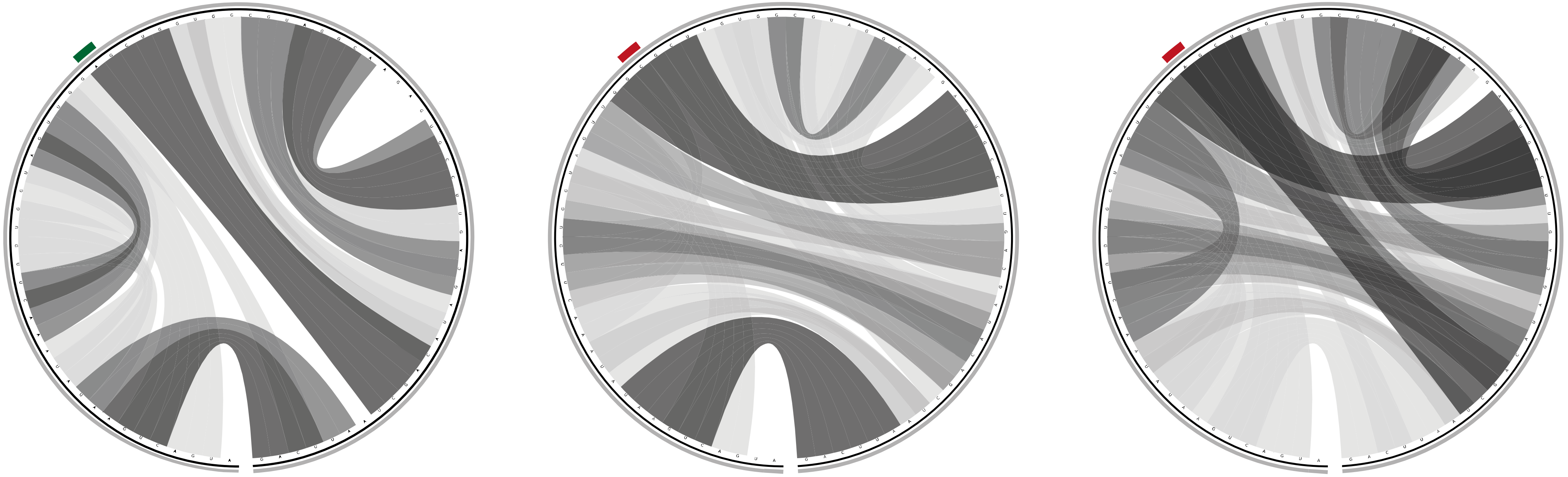

Supplementary Figure 6

Supplementary Figure 6: Base-pairing probability differences for *KRAS* c.30A>C

(a, b) Circos diagrams depict the base-pairing probability for the wildtype and mutant *KRAS* transcripts as an alternative visualization of the base-pair probability dotplots in Fig. 6b. The intensity of the arcs corresponds to the probabilities. **(c)** In the differential base-pairing probability circos plot, the arcs represent the absolute difference of base-pairing probabilities between wildtype and mutant *KRAS*.

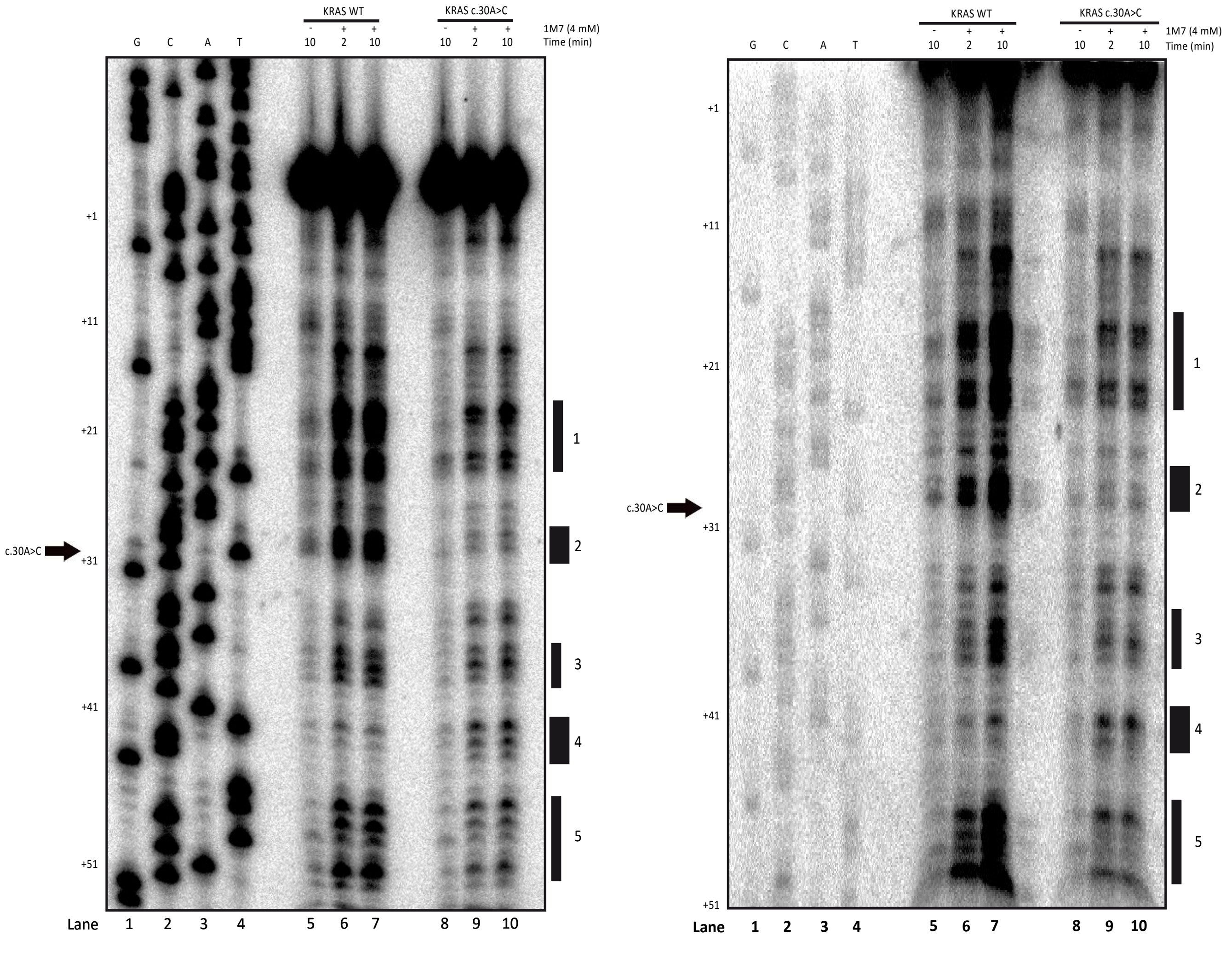

Supplementary Figure 7

Supplementary Figure 7: KRAS c.30A>C affects the transcript secondary structure

Two additional independent biological replicates of *in vitro* SHAPE probing for WT *KRAS* and mutant *KRAS* (c.30A>C) using 1M7 show differential nucleotide accessibility profiles. Lanes 5-7 and lanes 8-10 indicate the SHAPE profile of wildtype *KRAS* and mutant *KRAS* (c.30A>C), respectively. RNA shown in lanes 5 and 8 are treated with DMSO for 10 mins and lanes 6 / 9 and lanes 7 / 10 correspond to RNA treated with the SHAPE reagent 1M7 (4 mM final concentration) for 2 mins and 10 mins, respectively. Numbered rectangular boxes correspond to regions of predicted local structural accessibility changes as shown in Fig. 6c. Lanes 1-4 represent the sequencing ladder prepared from *KRAS* DNA as template.

a

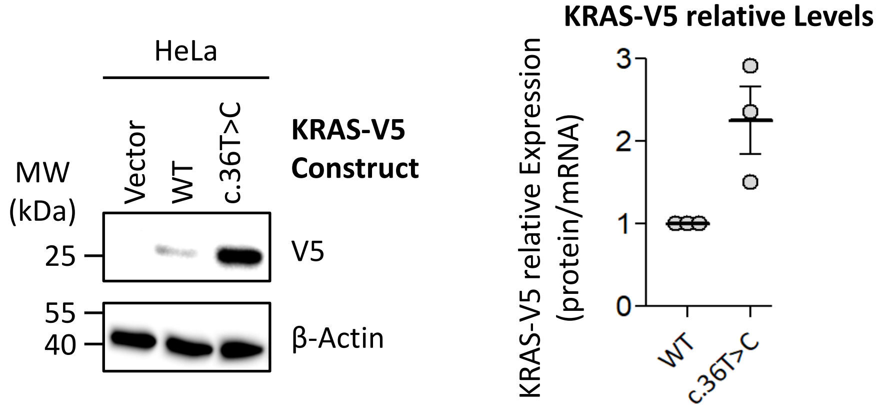

b

Synonymous Mutation Counts

	COSMIC	SynMICdb
c.39C>**A**	9	0
c.39C>**G**	3	0
c.39C>**T**	8	1

c

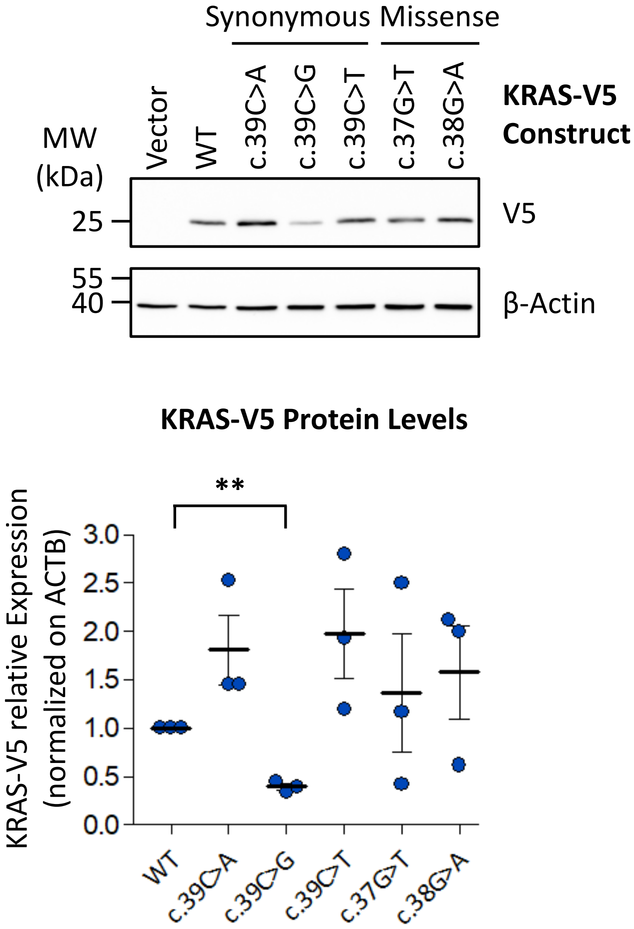

d

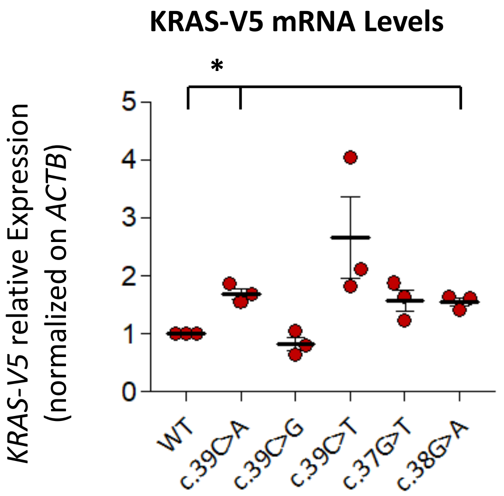

e

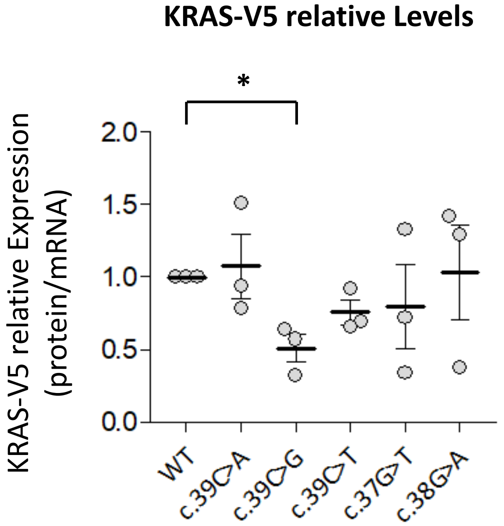

Supplementary Figure 8

Supplementary Figure 8: Impact of *KRAS* synonymous mutations in codons 12 and 13

(a) HeLa cells were transfected with either *KRAS* WT-V5, *KRAS* c.36T>C-V5 or the empty vector as a control. Top: Expression of KRAS-V5 was evaluated by western blotting using V5 and ACTB antibodies. A representative experiment is shown. Bottom: V5 western blot signals were quantified and normalized to ACTB signals. Additionally, *KRAS-V5* mRNA levels were measured by RT-qPCR and normalized to *ACTB* signals. KRAS-V5 relative expression was obtained after normalization of the protein signals on the respective mRNA levels as described in Fig. 7d. Mean of three independent experiments with error bars: SEM. **(b)** *KRAS* codon 13 synonymous mutation counts in the COSMIC database v82 (including targeted sequencing of this hotspot mutation codon) and in SynMICdb (mutations derived only from whole genome sequencing studies). **(c)** HEK293 cells were transfected with the indicated *KRAS-V5* mutants. Top: Expression of the constructs was evaluated by western blotting using V5 and ACTB antibodies. A representative experiment is shown. Bottom: Quantification of the western blot signals obtained as in top panel normalized to ACTB signals. Mean of three independent experiments with error bars: SEM. ** $p \leq 0.01$ (t-test). **(d)** *KRAS-V5* mRNA levels in the samples described in (c) determined and normalized to *ACTB* signals. Mean of three independent experiments with error bars: SEM. * $p \leq 0.05$ (t-test). **(e)** KRAS-V5 codon 13 mutants relative expression was obtained after normalization of the protein signals for each mutant on their respective mRNA levels. Mean of three independent experiments with error bars: SEM. * $p \leq 0.05$ (t-test).

Supplementary Note 1: User Guide

A detailed User Guide for the SynMICdb including explanatory screenshots is provided.

SynMICdb
Synonymous Mutations In Cancer database

The Synonymous Mutations In Cancer database (SynMICdb) is a curated database of synonymous mutations in cancer. SynMICdb allows biologists to easily extract and download synonymous mutations in cancer as well as orthogonal data using multiple search options. It also integrates the predicted impact of synonymous mutations on structural changes in RNA using structural prediction algorithms.

Several independent search criteria are available in SynMICdb such as the gene name, the genomic coordinates, the position of the mutations within the coding sequence (CDS), their evolutionary conservation, the organ system, organ and tumor type, their link to cancer (Cancer Gene Census) or the SynMICdb score. Each search option is described in detail below.

Search by Gene

This feature allows the user to search for synonymous mutations present in a gene of interest using one of the following nomenclatures (Figure 1):

1. HGNC gene symbol
2. Gene name
3. ENSEMBL ID

Alias names for genes (P53 for TP53) are allowed and the search is case-insensitive.

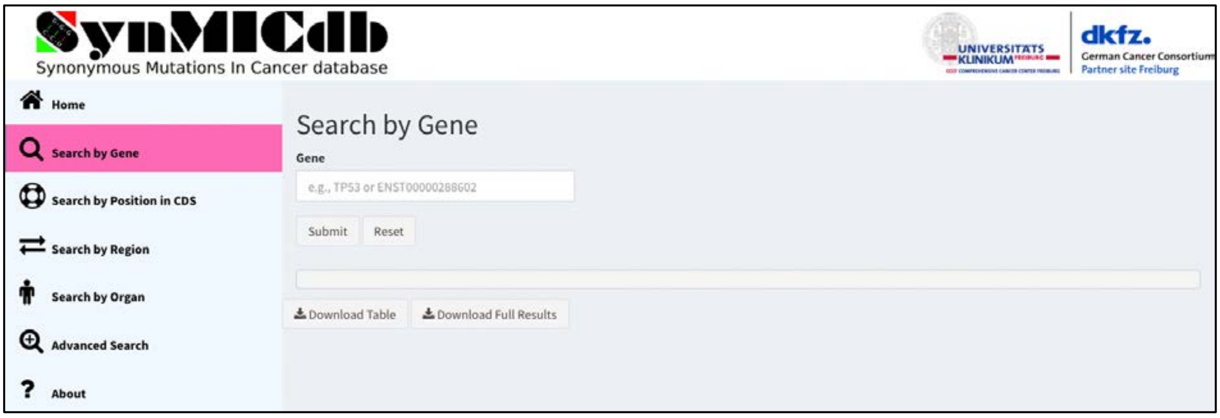

Figure 1. Search option "Search by Gene".

For example, Figure 2 shows the results page for the gene *KRAS*. The summary information in Cancer Gene Census[1] for the gene is shown. The link to Genecards[2] for the gene is also provided.

[1] http://cancer.sanger.ac.uk/census/

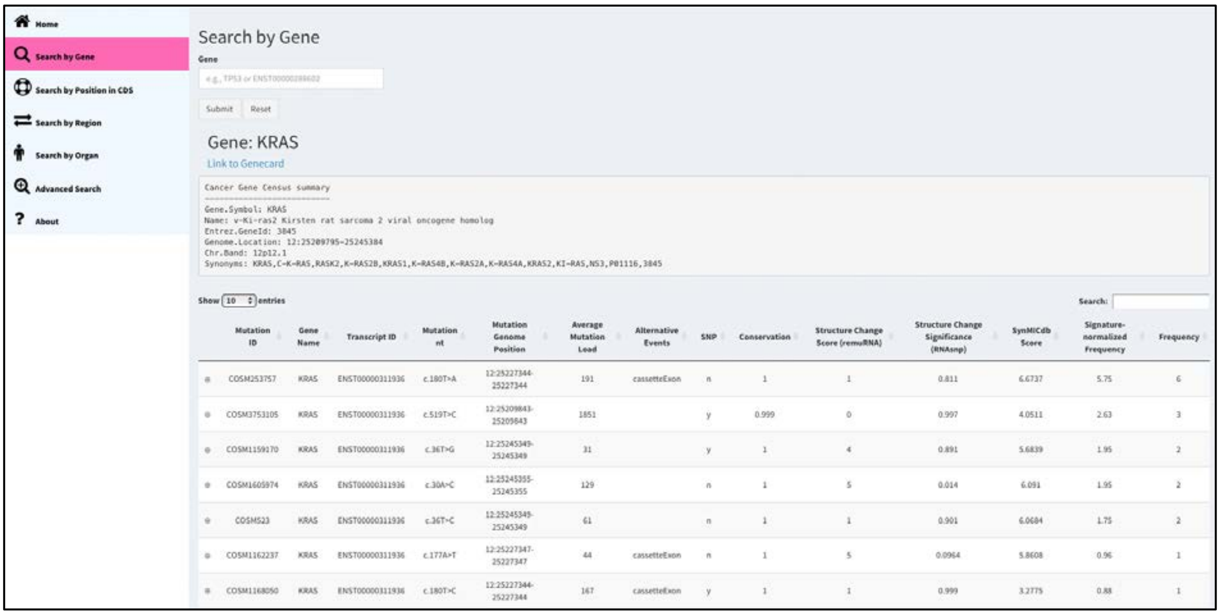

Figure 2. Example of result page for "Search by Gene" in SynMICdb.

The result columns provide the following information:

- **Mutation ID**: Unique identifier of each mutation (as present in COSMIC database).
- **Gene Name**: Abbreviated name of the gene.
- **Transcript ID**: ENSEMBL transcript ID for the corresponding mutation.
- **Mutation nt**: Number and nucleotide change of mutation: e.g. c.36T>G indicates a change of coding nucleotide number 36 from T to G.
- **Mutation genome position**: Genomic coordinates of each respective mutation in human genome assembly GRCh38 (chromosome:start-end).
- **SynMICdb Score**: The SynMICdb score shall reflect the probable impact of the synonymous mutation and is based on the mutation frequency, the probability due to mutational bias by mutation signatures, the average mutation load of the tumors with this mutation, the evolutionary conservation, the listing of the affected gene as cancer gene in the Cancer Gene Census, the listing of the mutation in the SNPdb, the FATHMM-MKL score, the CADD score and the predicted impact on RNA secondary structure. The score ranges from -4 to +12 and high numbers indicate a higher likelihood of a functional impact of the synonymous mutation. The distribution of the SynMICdb score is illustrated by the following table and violin plot:

Quantile	SynMICdb Score
top 50%	0.89
top 25%	1.83
top 10%	2.70
top 1%	4.38
top 0.1%	5.83
top 0.01%	8.08

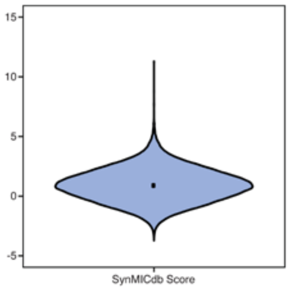

Thus, a SynMICdb score of above 4.38 indicates that the synonymous mutation is among the top 1% of synonymous mutations in this study.

[2] http://www.genecards.org/

- **Average Mutation Load**: This column indicates the average number of mutations found in the genome-wide analysis of the tumor samples harboring this specific mutation.
- **Alternative Events**: This column provides information about alternative events as indicated by GENCODE like alternative splicing and other events that result in more than a single transcript from the same gene characterized by the UCSC genome browser[3].
- **SNP**: This column provides information whether this mutation has been listed as a Single Nucleotide Polymorphism (SNP) in the SNP database. y = yes, n = no.
- **Conservation**: This column lists the conservation scores of human vs. 99 vertebrate genomes (PhastCons100). The score ranges between 0 to 1 with 1 indicating the highest conservation levels among the 100 species.
- **Structure Change Score (remuRNA)**: This column depicts scores for structural change predictions for the respective mutation calculated by remuRNA. The score ranges from -5 to +20 and high numbers indicate a higher likelihood of a structural change caused by the mutation.
- **Structure Change Significance (RNAsnp)**: This column has *p-values* for significant structural change predictions for the respective mutation calculated by RNAsnp p0. The p-value ranges from 0 to 1 and low numbers indicate a higher likelihood of a structural change caused by the mutation.
- **Exon Type**: This column displays information about the exon type (1 = first exon, 2 = internal exon, 3 = last exon, 4 = monoexonic transcript).
- **Distance to Closest Exon Bundary**: This column indicates the distance to the closest exon boundary for each synonymous mutation in nucleotides.
- **Any ESE/ESS Change**: This column lists the gains and losses of exonic splicing enhancer (ESE) or exonic splicing silencer (ESS) motifs according to RegRNA 2.0 or SpliceAidF. Details for this analysis for ESEs and ESSs separately for the two prediction algorithms are provided in the full data table upon "Download Full Results". Please note that 23 motifs were assigned "ESE" as well as "ESS" properties in SpliceAidF and hence are listed separately as "ESE & ESS".
- **Signature-normalized Frequency**: In this column, the Frequency of the mutation has been corrected for the mutation bias due to mutational signatures frequently observed in cancer - thus, the Frequency has been multiplied with (1 - p) with p indicating the probability of the nucleotide change according to the most prevalent mutational signature in cancer.
- **Frequency**: This column shows the recurrence level of each mutation. The number in this column represents the total number of tumor samples in which the respective mutation was found.

By default, the results are grouped by Mutation ID and sorted by their frequency. For each Mutation ID, only one line is given in this view.
Detailed information for each sample can be viewed by clicking on the ⊕ icon.
Figure 3 shows an example of sample information for mutation ID COSM253757.

[3] For more details, please visit: https://genome.ucsc.edu/cgi-bin/hgTrackUi?hgsid=603403771_9k9O4FUq13hjAk0gvJCQPmO4vctG&c=chr12&g=knownAlt

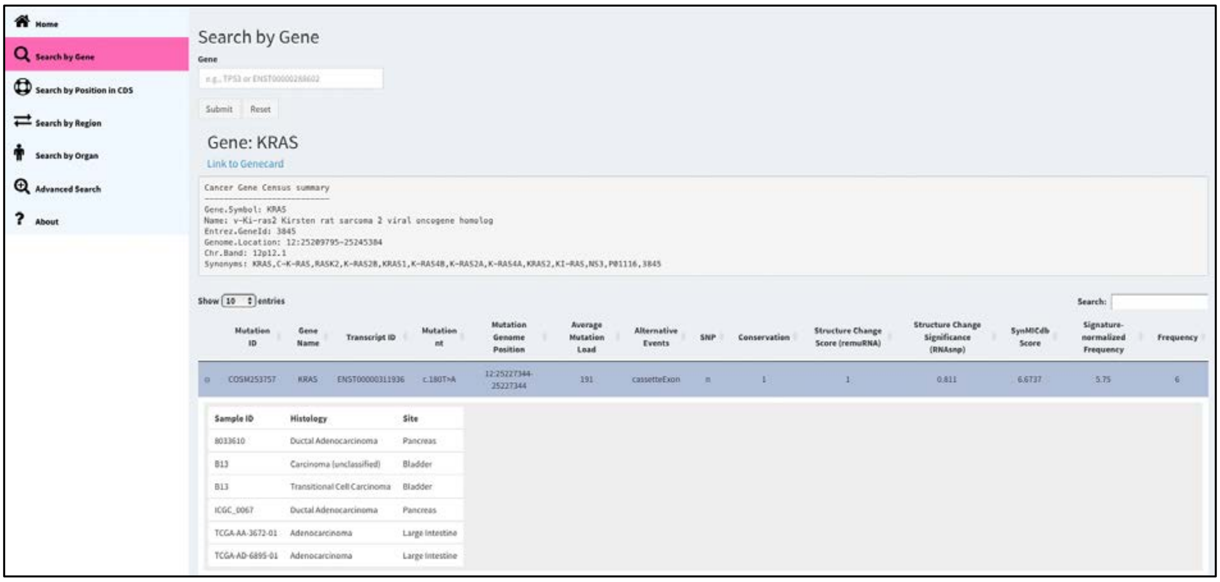

Figure 3. Detailed sample information for all samples having the Mutation ID COSM253757 in the table of results.

Download Options

The user can download the results using one of the following two options:

- **Download Table**: This button allows the user to download the displayed results as a csv file.
- **Download Full Results**: This button allows user to download the displayed results plus additional information like affected codon and amino acid, the mutation load of each affected sample, the position of the mutation within the CDS as well as the classification by the Cancer Gene Census (CGC).

Search by Position in CDS

This option allows the user to search for mutations on the basis of their location within the coding sequence (CDS) of genes (e.g. Figure 4 shows mutations present within the first 20% of the CDS). This facilitates the user to study synonymous mutation within a specific region of interest, for example towards the 5'-end of the coding region within the translation initiation and ramping region.

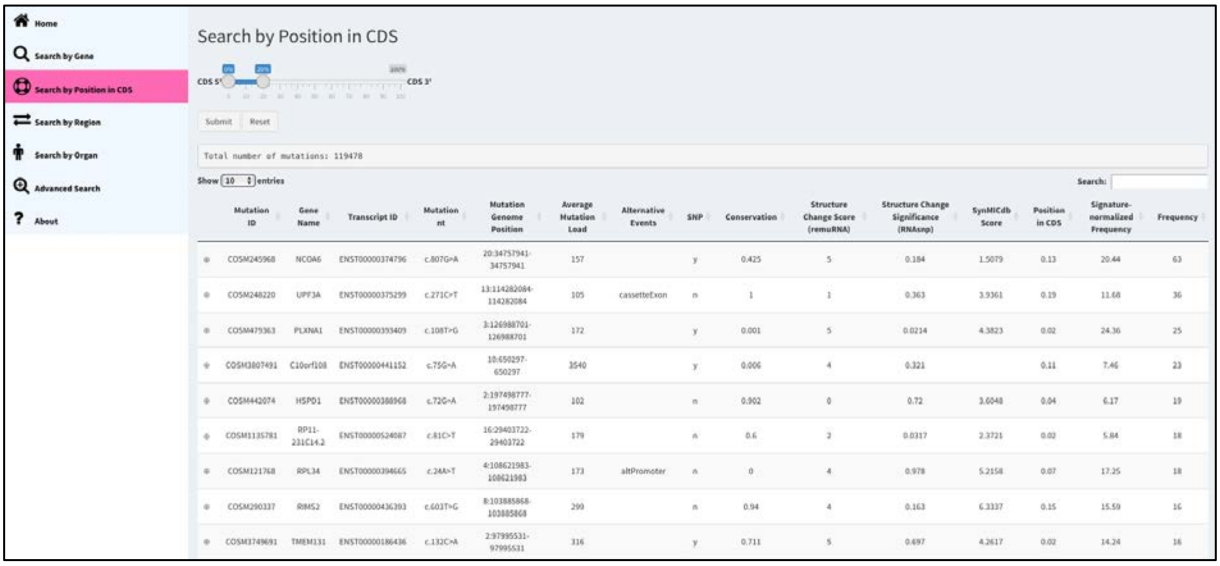

Figure 4. Example of results page for "Search by Position in CDS".

Search by Region

This option allows the user to search for mutations present within a region defined by genomic coordinates of human genome assembly GRChg38 (note: chromosome 23 = X, 24 = Y and 25 = M). For example, Figure 5 shows the list of mutations present in chromosome 5 region 50000-500000.

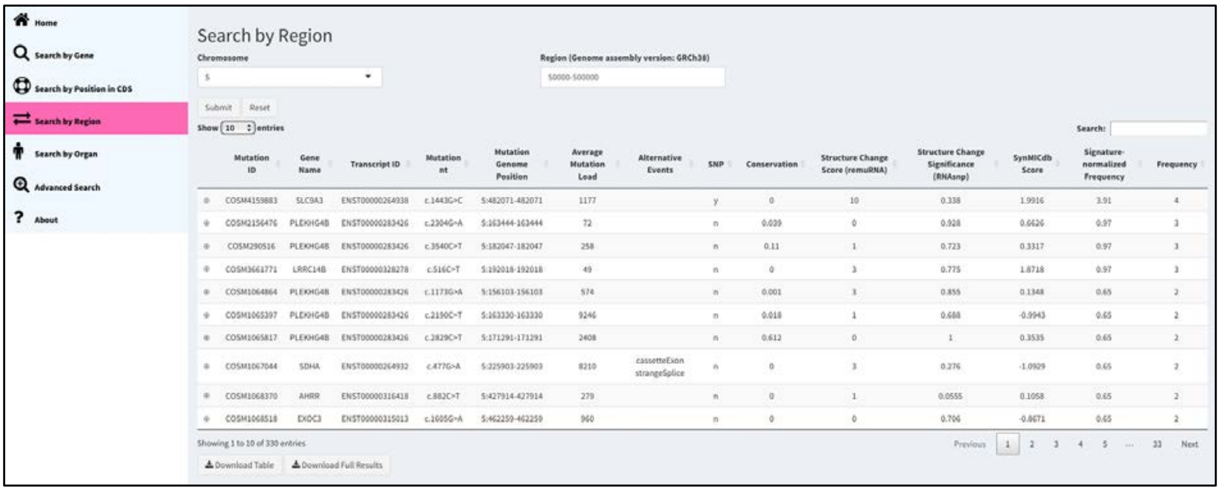

Figure 5. "Search by Region" using genome coordinates.

Search by Organ

This option allows the user to search for synonymous mutations in cancer on the basis of their site of origin in a hierarchical manner. The user first selects an organ system and then a site and histology of interest. Nine organ systems are listed (as depicted in Figure 6): Cardiovascular System, Digestive System, Endocrine System, Genitourinary System, Integumentary System, Lymphatic System, Musculoskeletal System, Nervous System and Respiratory System.

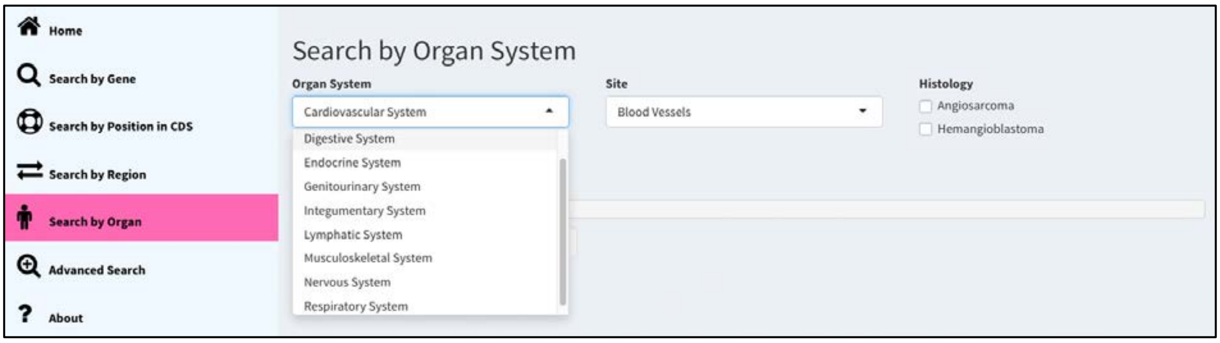

Figure 6. "Search by organ" - selection of the organ system of interest.

After selecting the organ system, the user selects first the primary site and optionally the histology of interest. The following example depicts a search and result of synonymous mutations present in the "Digestive System" as organ system following the selection of the "Large Intestine" as primary site (Figure 7) and "Adenocarcinoma" as histology (Figure 8).

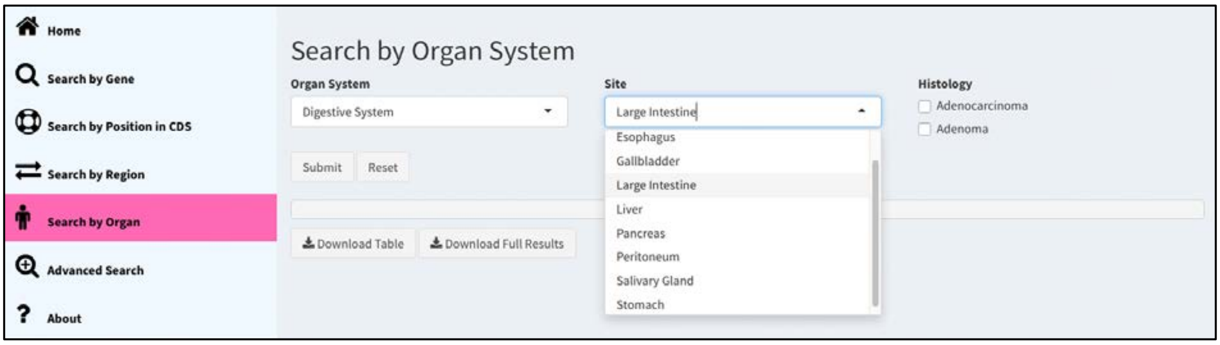

Figure 7. "Search by Organ" - selection of primary site.

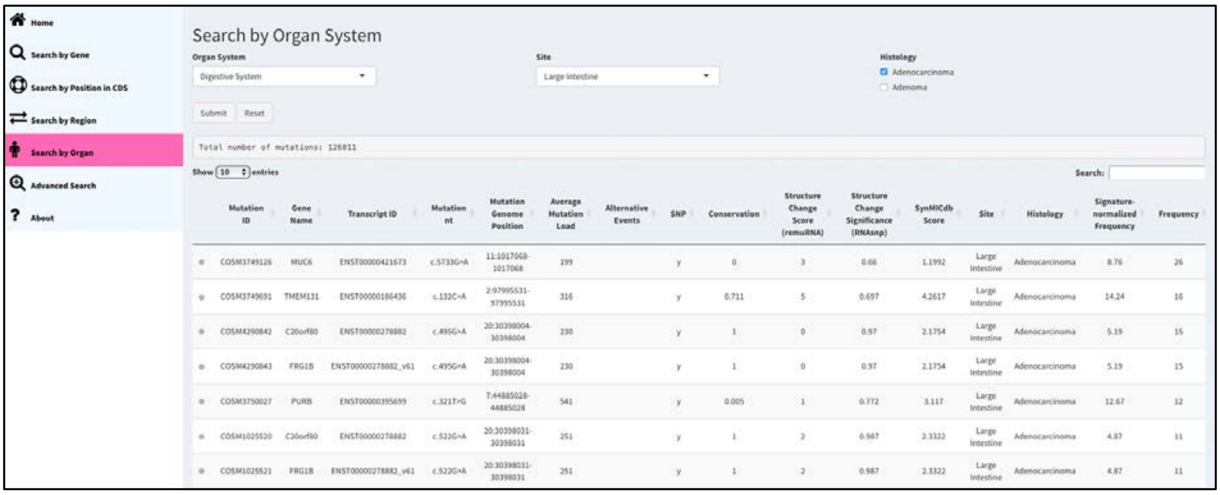

Figure 8. "Search by Organ" - selection of primary histology and results.

Advanced search

This search option allows the combination of multiple search parameters and offers additional search criteria including Gene Names, Cancer Gene Census genes, Conservation, Location within CDS, SynMICdb Score, Organ System, Site, and Histology of synonymous mutations. Here, users can also perform batch searches by providing a list of up to 100 genes (Figure 9).

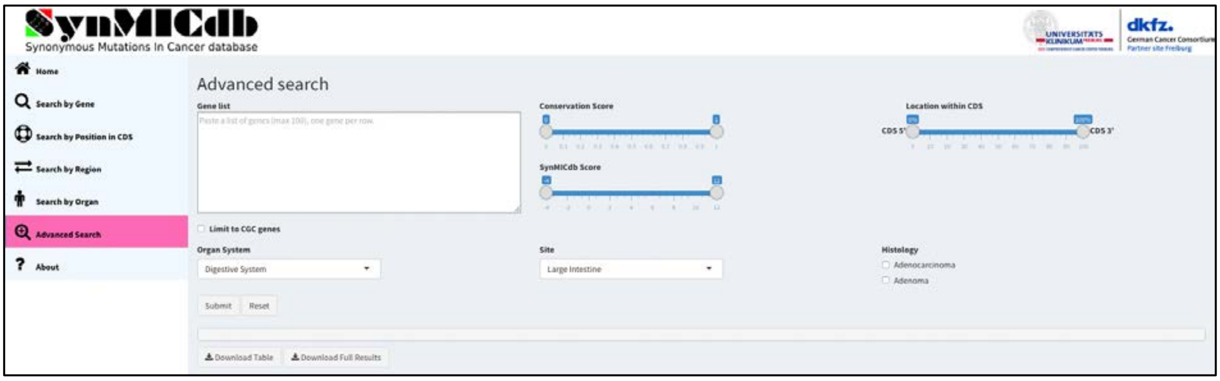

Figure 9. Panel of the "Advanced Search".

Below is an example of search for synonymous mutations that are >80% conserved and only present in the first 30% of the CDS (Figure 10). The user can limit the output to genes listed as cancer genes in the Cancer Gene Census (CGC) database by clicking the "Limit to CGC genes" option.

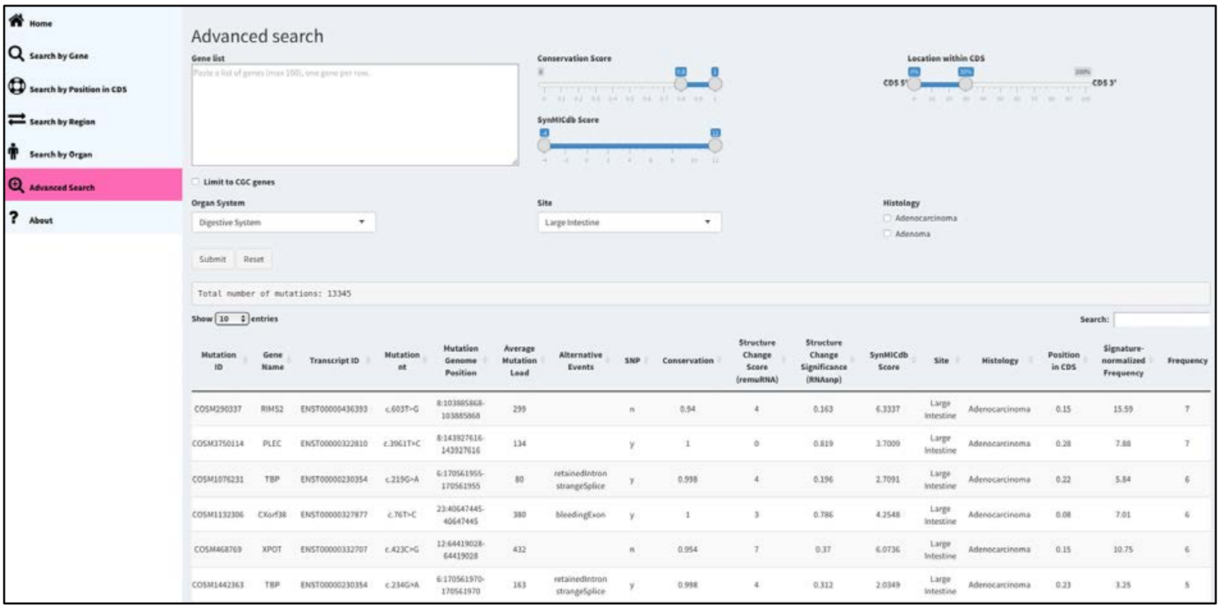

Figure 10. "Advanced Search" - results listing synonymous mutations in the large intestinal tumors with a conservation score >= 0.8 and mutation position within the first 30% from the 5' end of the CDS.
