# Supplementary material for: A pan-cancer analysis of synonymous mutations
Source: Nat Commun. 2019 Jun 12;10:2569. doi: 10.1038/s41467-019-10489-2 (PMC6562042; doi:10.1038/s41467-019-10489-2)
Supplement: Supplementary file 2 — Description of Additional Supplementary Files [file 41467_2019_10489_MOESM2_ESM.pdf]

Description of Additional Supplementary Files

File name: Supplementary Data 1

Description: List of mutation frequencies derived from the COSMIC dataset found in whole-genome sequencing studies after removal of duplicates and as depicted in Fig. 1a.

File name: Supplementary Data 2

Description: Overview of the curation process for synonymous mutations from the COSMIC dataset found in whole-genome sequencing studies with removal of duplicates and misannotated mutations resulting in 659194 synonymous mutations cataloged in SynMICdb.

File name: Supplementary Data 3

Description: List of 316 synonymous mutations occurring altogether 470 times in SynMICdb found in human cancer overlapping sites with known genetic associations to tumor-related phenotypes as found in the ClinVar database.

File name: Supplementary Data 4

Description: List of sequence motifs for exonic splicing enhancers (ESE) or exonic splicing silencers (ESS) used in this study. After curation for duplicates, 76 motifs from RegRNA 2.0 and 111 motifs from SpliceAidF were used. The annotation from SpliceAidF (e.g. regarding interaction partners) were concatenated for motifs listed multiple times. Please note that 23 motifs were assigned "ESE" as well as "ESS" properties in SpliceAidF (marked in red).

File name: Supplementary Data 5

Description: Aggregated results of the comparison of wildtype and mutant sequences for exonic splicing enhancers (ESE) or exonic splicing silencers (ESS) for all synonymous mutations for gains or losses of ESE or ESS motifs derived from RegRNA 2.0 or SpliceAidF. Additionally, the mutation ID, the transcript ID, the gene name, the mutation, the SynMICdb score, the exon type (1 = first exon, 2 = internal exon, 3 = last exon, 4 = monoexonic) and the distance to the closest exon boundary are provided for each synonymous mutation. Please note that 23 motifs were assigned "ESE" as well as "ESS" properties in SpliceAidF (marked in red).

File name: Supplementary Data 6

Description: Matrix of all synonymous mutations and their gain (+1) or loss (-1) for all ESE or ESS motifs derived from RegRNA 2.0.

File name: Supplementary Data 7

Description: Matrix of all synonymous mutations and their gain (+1) or loss (-1) for all ESE or ESS motifs derived from SpliceAidF.

File name: Supplementary Data 8

Description: Comparison of synonymous mutations overlapping between SynMICdb and found to be impacting splicing according to Jayasinghe et al., Cell Rep 2018. 46 synonymous mutations listed by Jayasinghe et al. were also found in SynMICdb and their SynMICdb score, average mutation load of the affected samples and alternative events are listed.
