# Supplementary material for: A pan-cancer analysis of synonymous mutations
Source: Nat Commun. 2019 Jun 12;10:2569. doi: 10.1038/s41467-019-10489-2 (PMC6562042; doi:10.1038/s41467-019-10489-2)
Supplement: Supplementary file 12 — Source Data [file 41467_2019_10489_MOESM12_ESM.zip › Source Data File.pptx]

## Slide 1
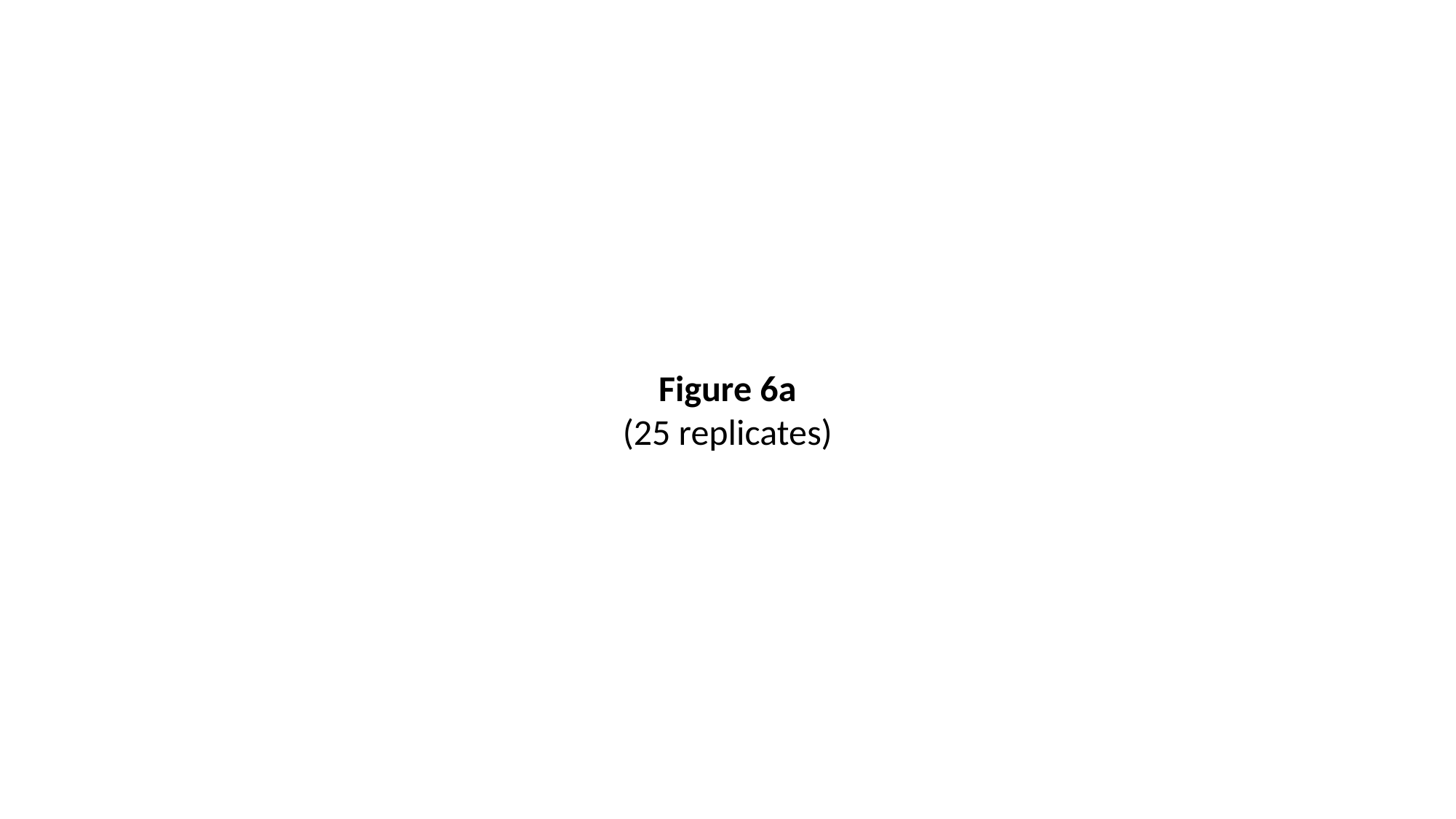

Figure 6a
(25 replicates)

## Slide 2
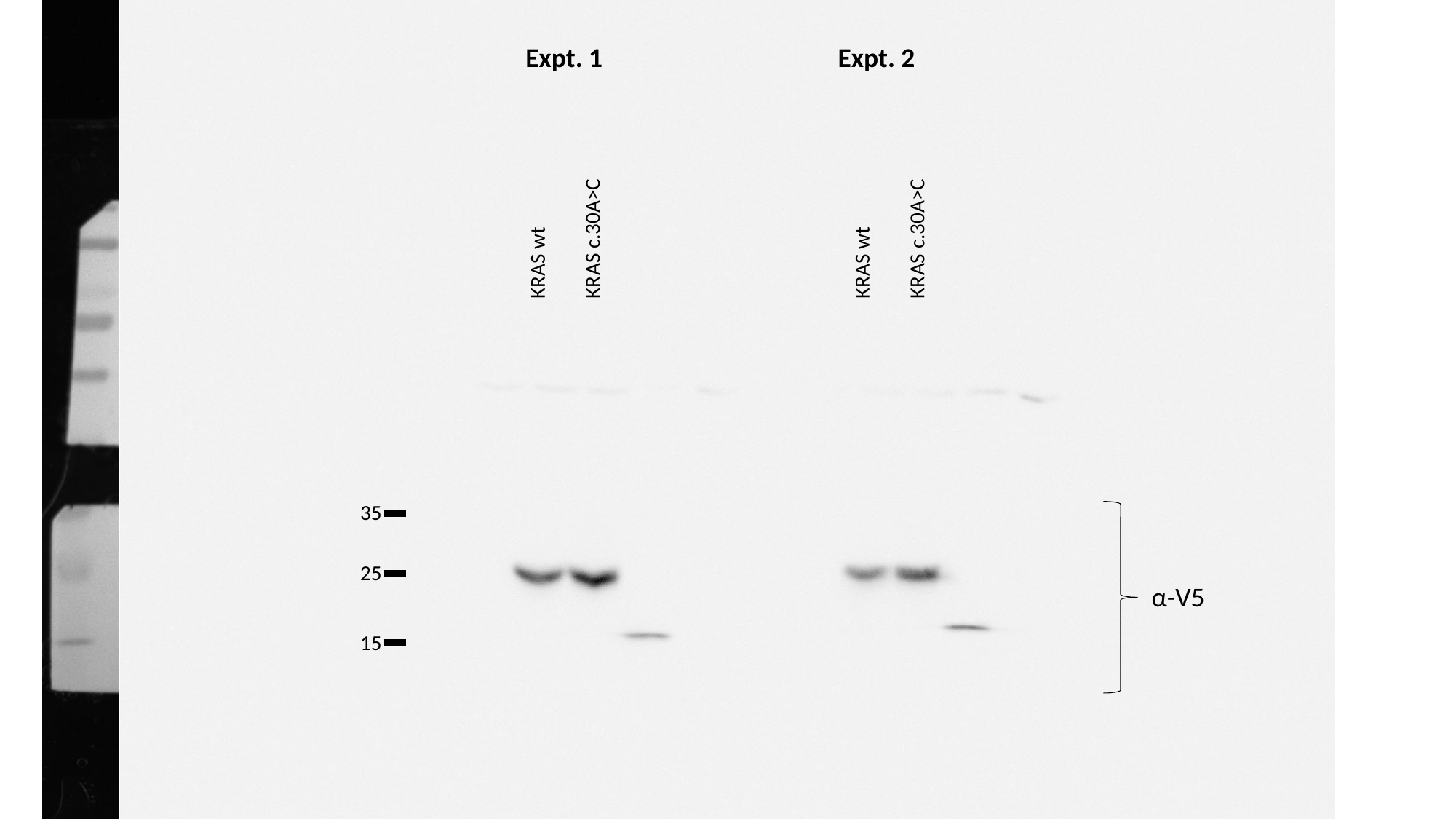

Expt. 1
Expt. 2
KRAS c.30A>C
KRAS wt
KRAS c.30A>C
KRAS wt
35
25
α-V5
15

## Slide 3
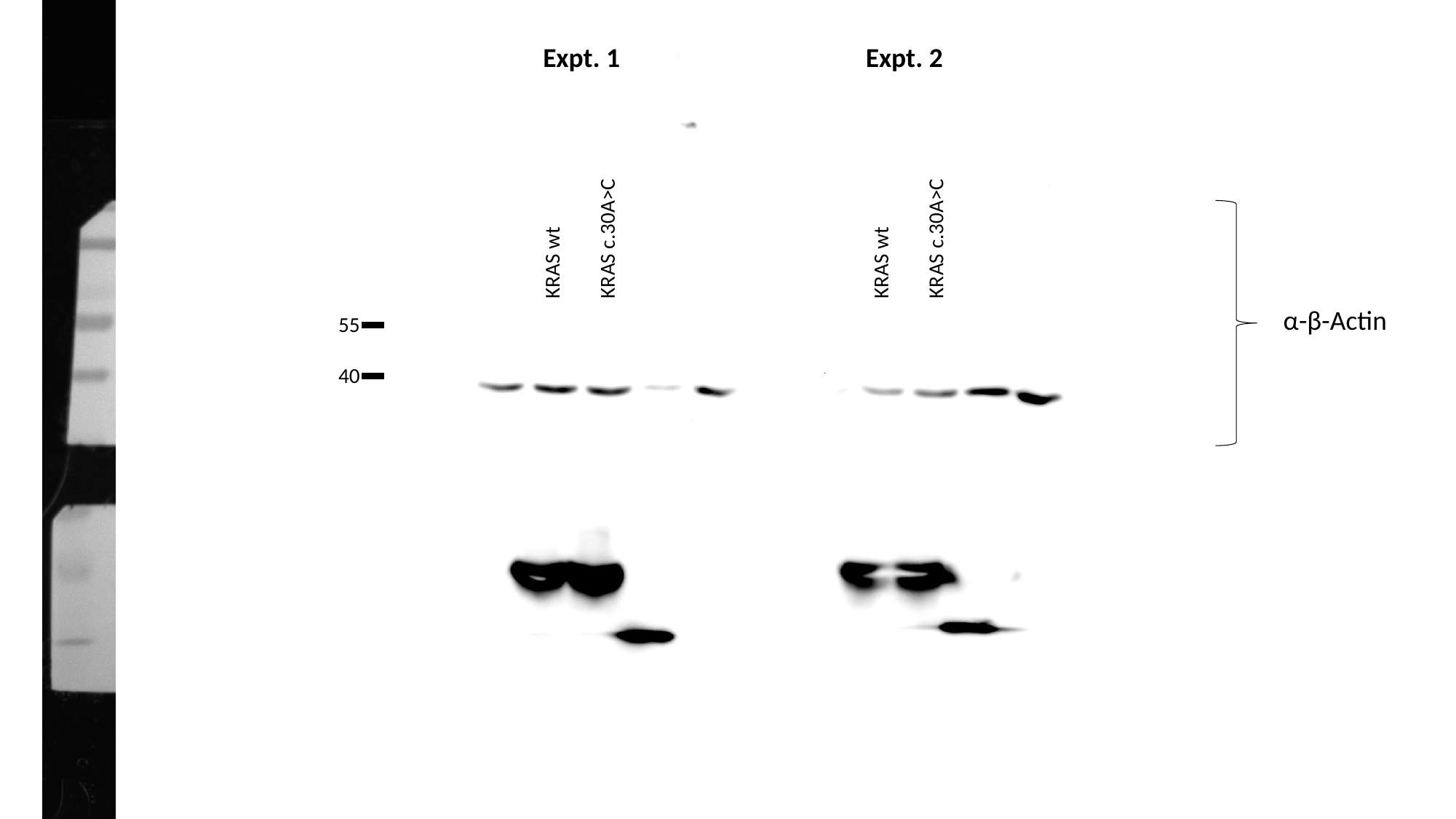

Expt. 1
Expt. 2
KRAS c.30A>C
KRAS wt
KRAS c.30A>C
KRAS wt
α-β-Actin
55
40

## Slide 4
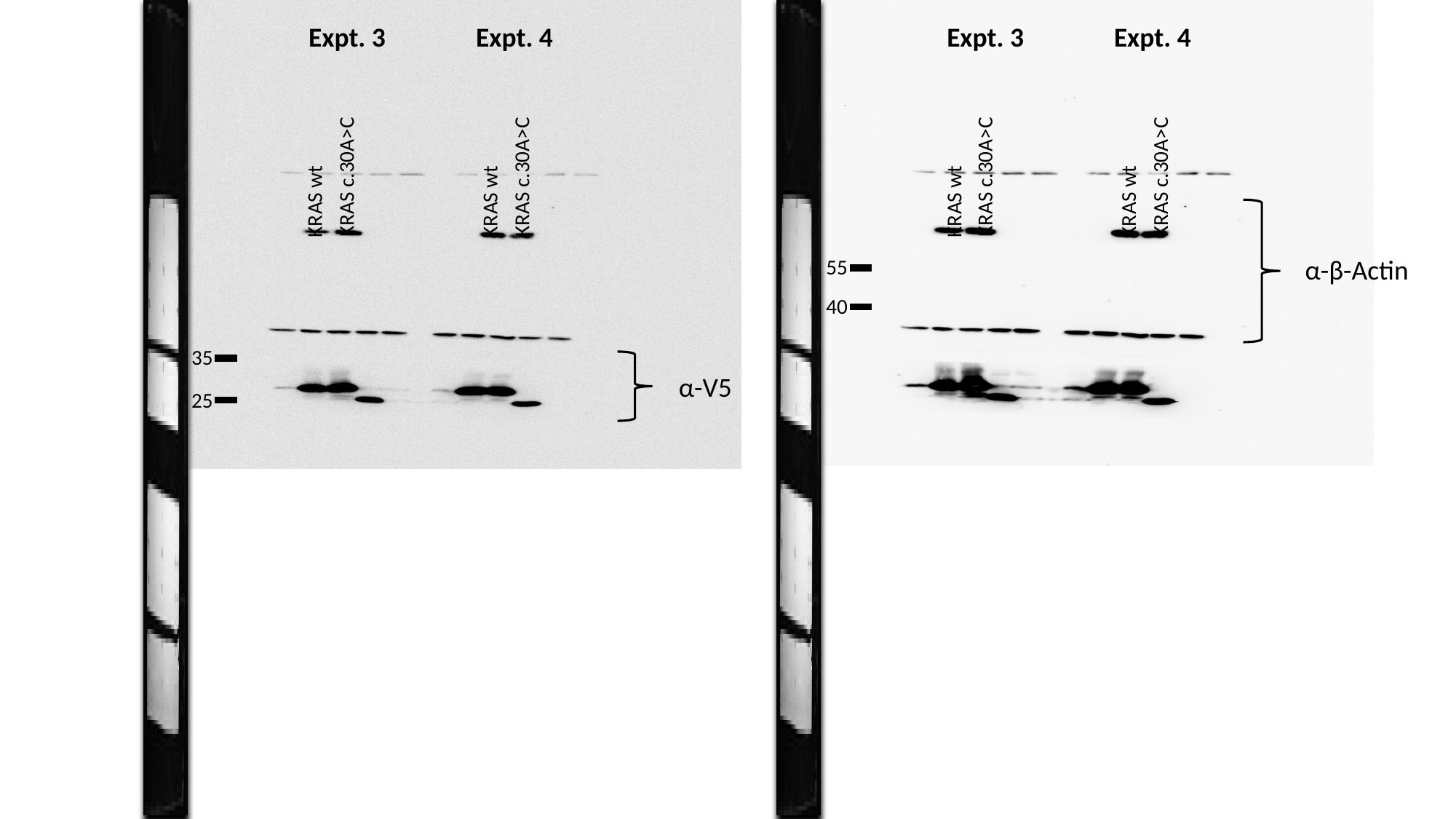

Expt. 3
Expt. 4
Expt. 3
Expt. 4
Expt. 3
Expt. 4
KRAS c.30A>C
KRAS wt
KRAS c.30A>C
KRAS wt
KRAS c.30A>C
KRAS wt
KRAS c.30A>C
KRAS wt
α-β-Actin
55
40
35
α-V5
25

## Slide 5
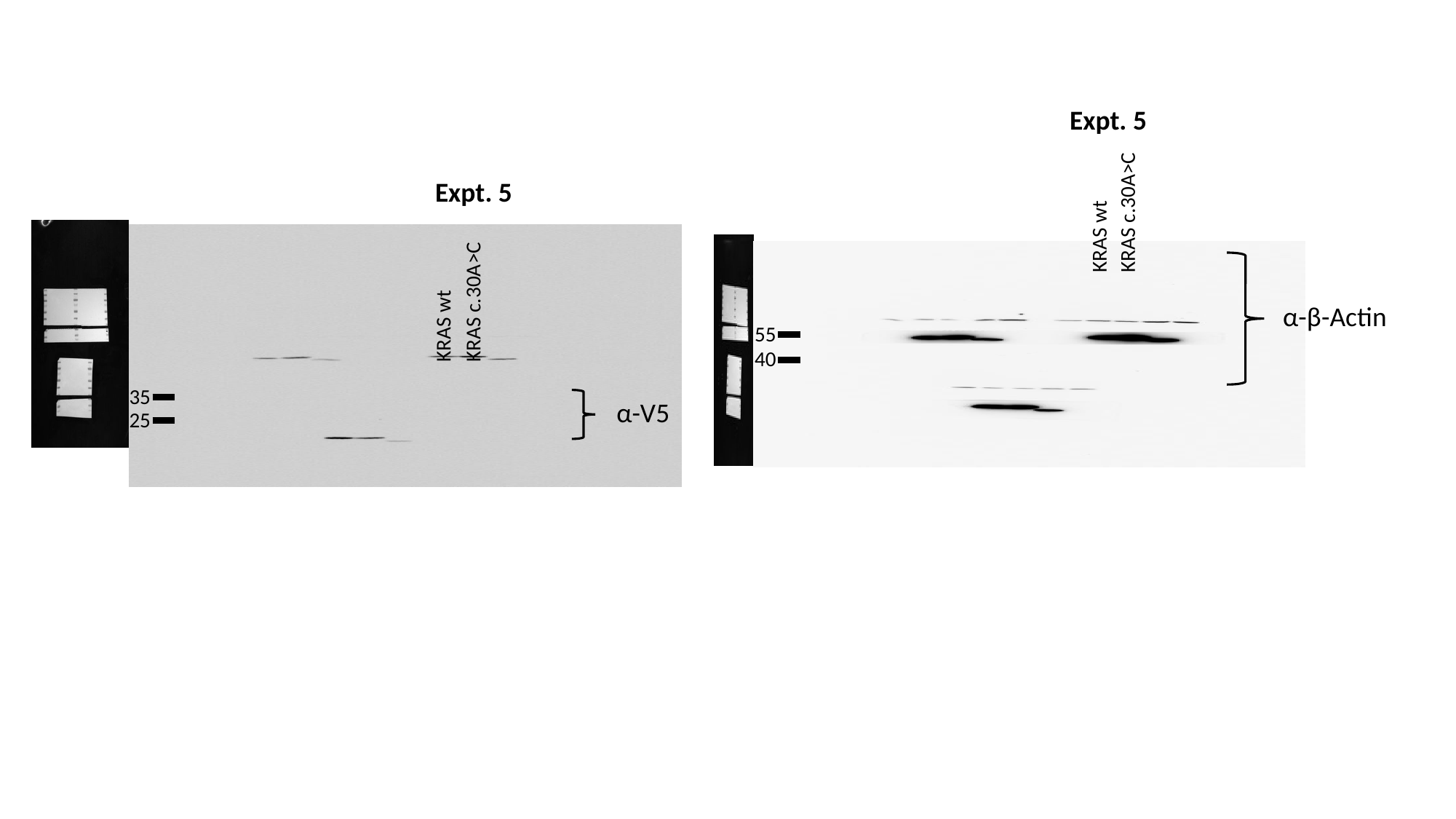

Expt. 5
KRAS c.30A>C
KRAS wt
α-β-Actin
55
40
Expt. 5
KRAS c.30A>C
KRAS wt
35
α-V5
25

## Slide 6
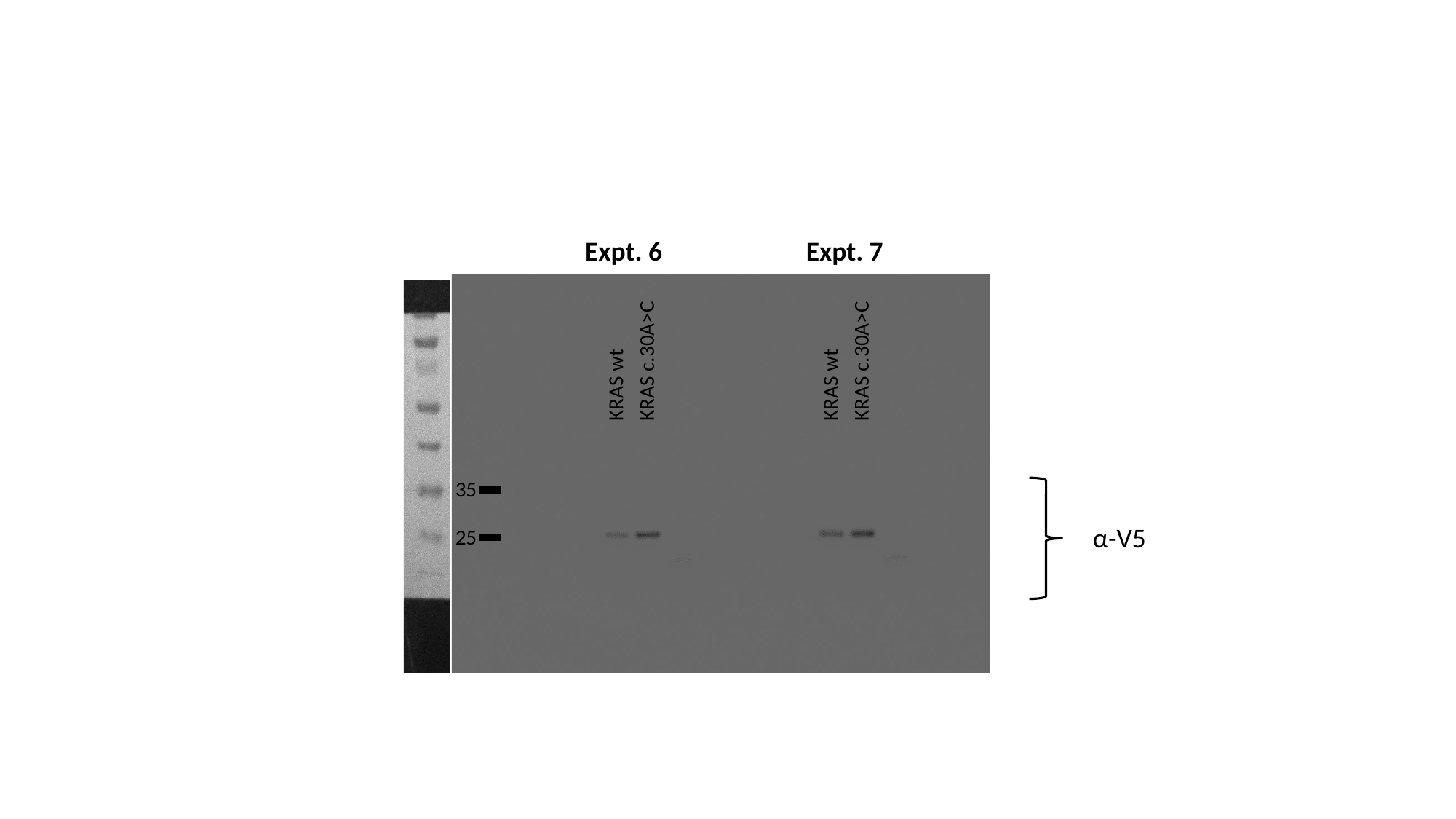

Expt. 6
Expt. 7
KRAS c.30A>C
KRAS wt
KRAS c.30A>C
KRAS wt
35
α-V5
25

## Slide 7
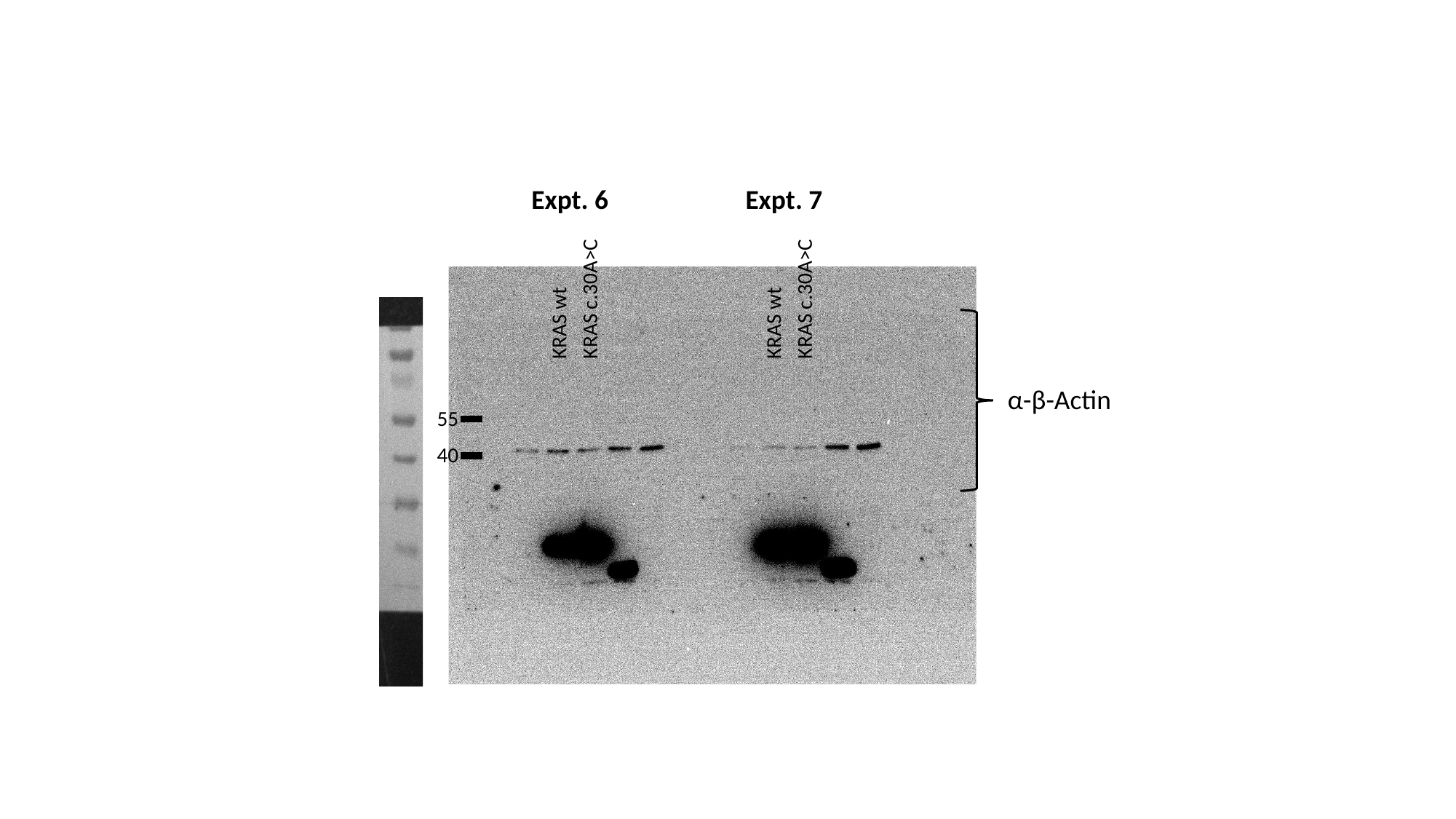

Expt. 6
Expt. 7
KRAS c.30A>C
KRAS wt
KRAS c.30A>C
KRAS wt
α-β-Actin
55
40

## Slide 8
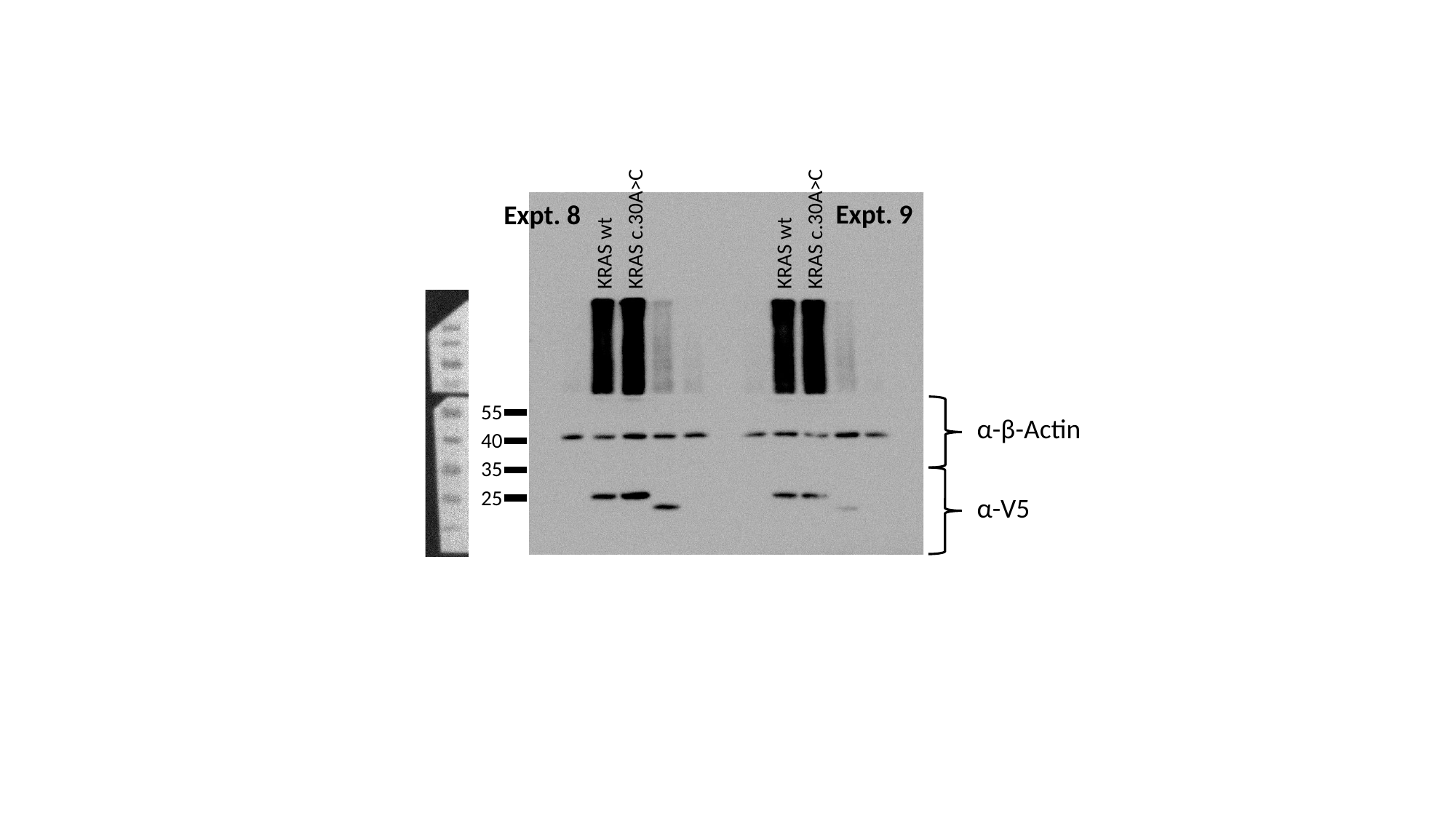

KRAS c.30A>C
KRAS wt
KRAS c.30A>C
KRAS wt
Expt. 9
Expt. 8
55
α-β-Actin
40
35
25
α-V5

## Slide 9
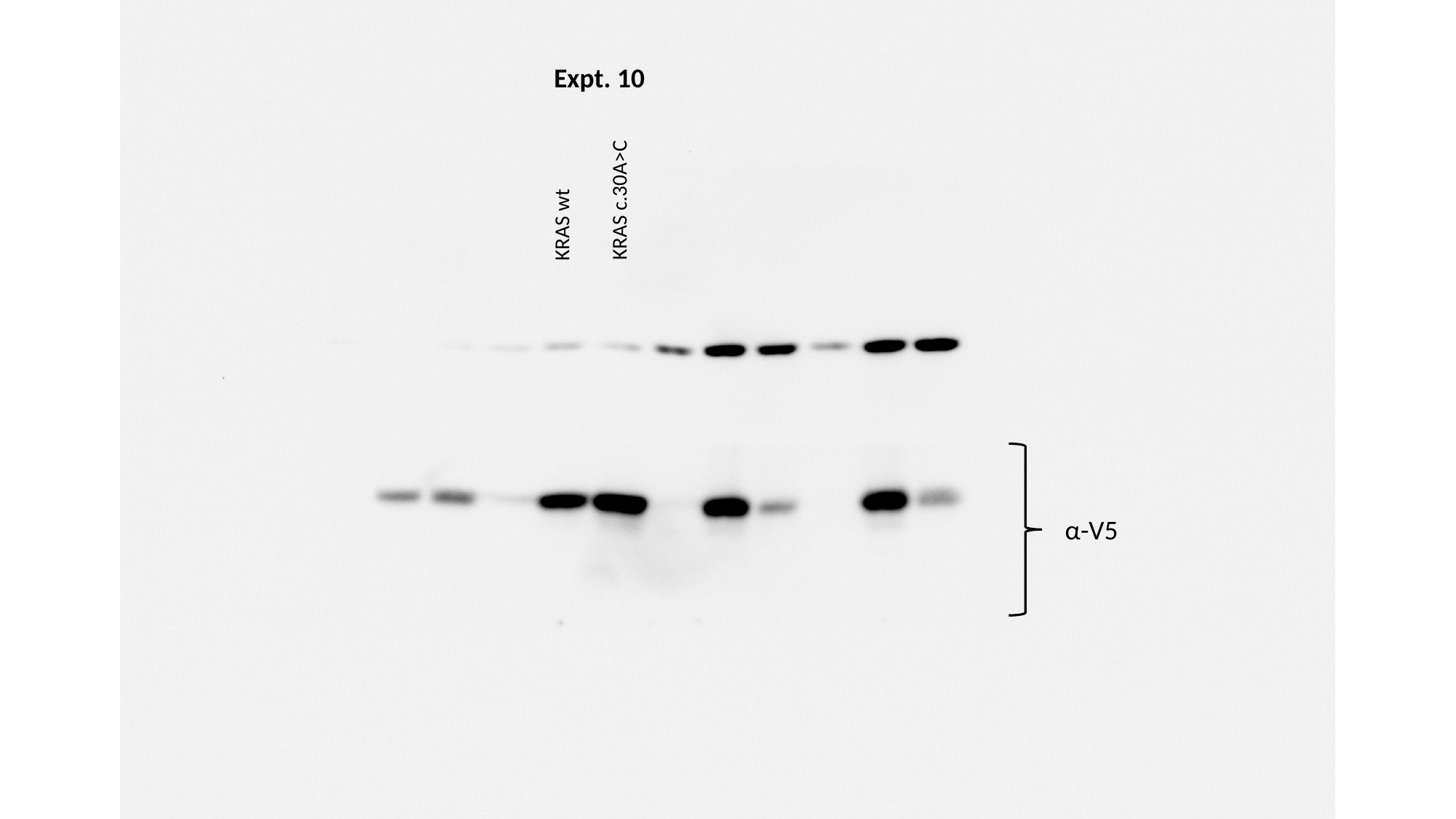

Expt. 10
KRAS c.30A>C
KRAS wt
α-V5

## Slide 10
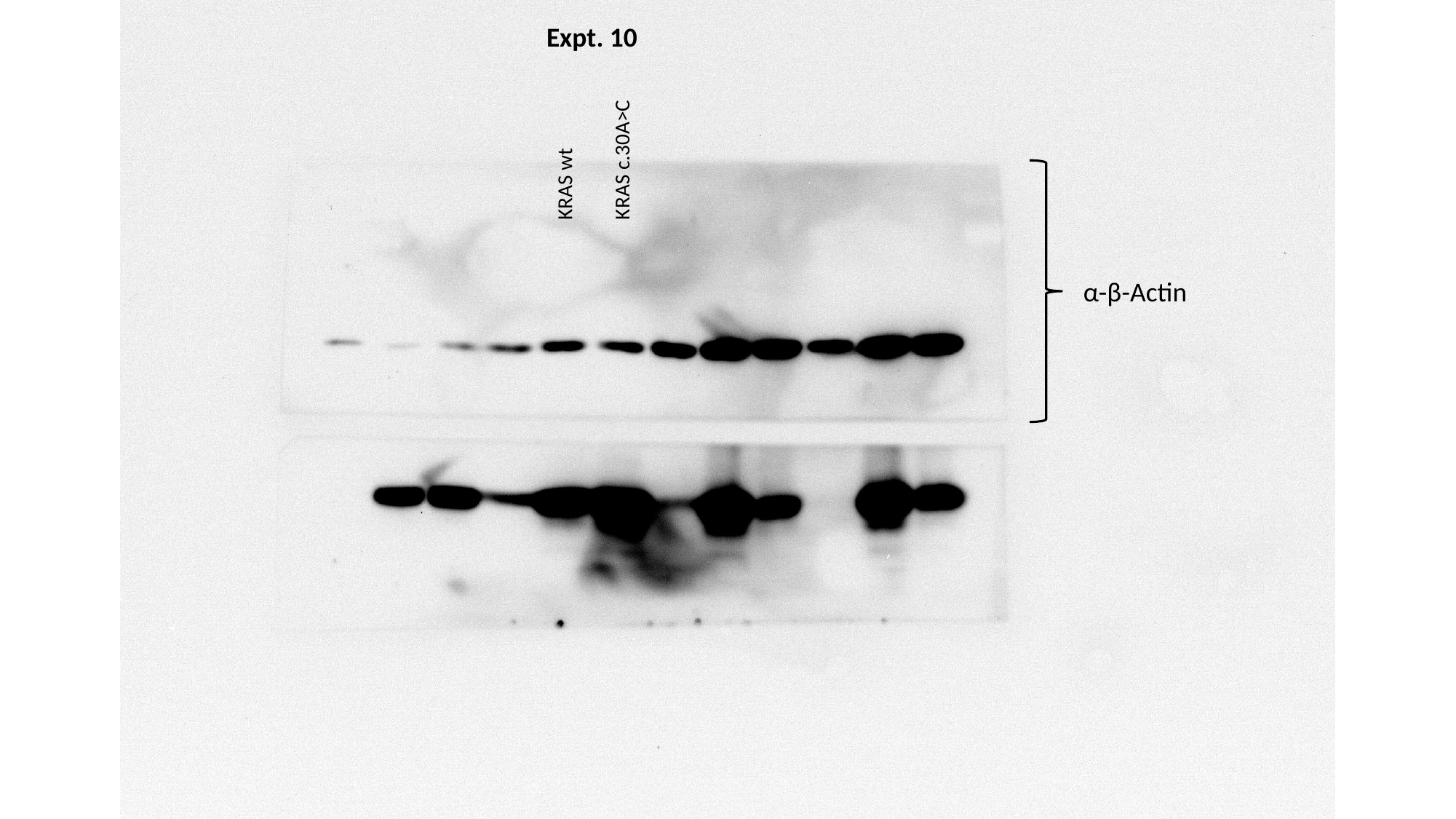

Expt. 10
KRAS c.30A>C
KRAS wt
α-β-Actin

## Slide 11
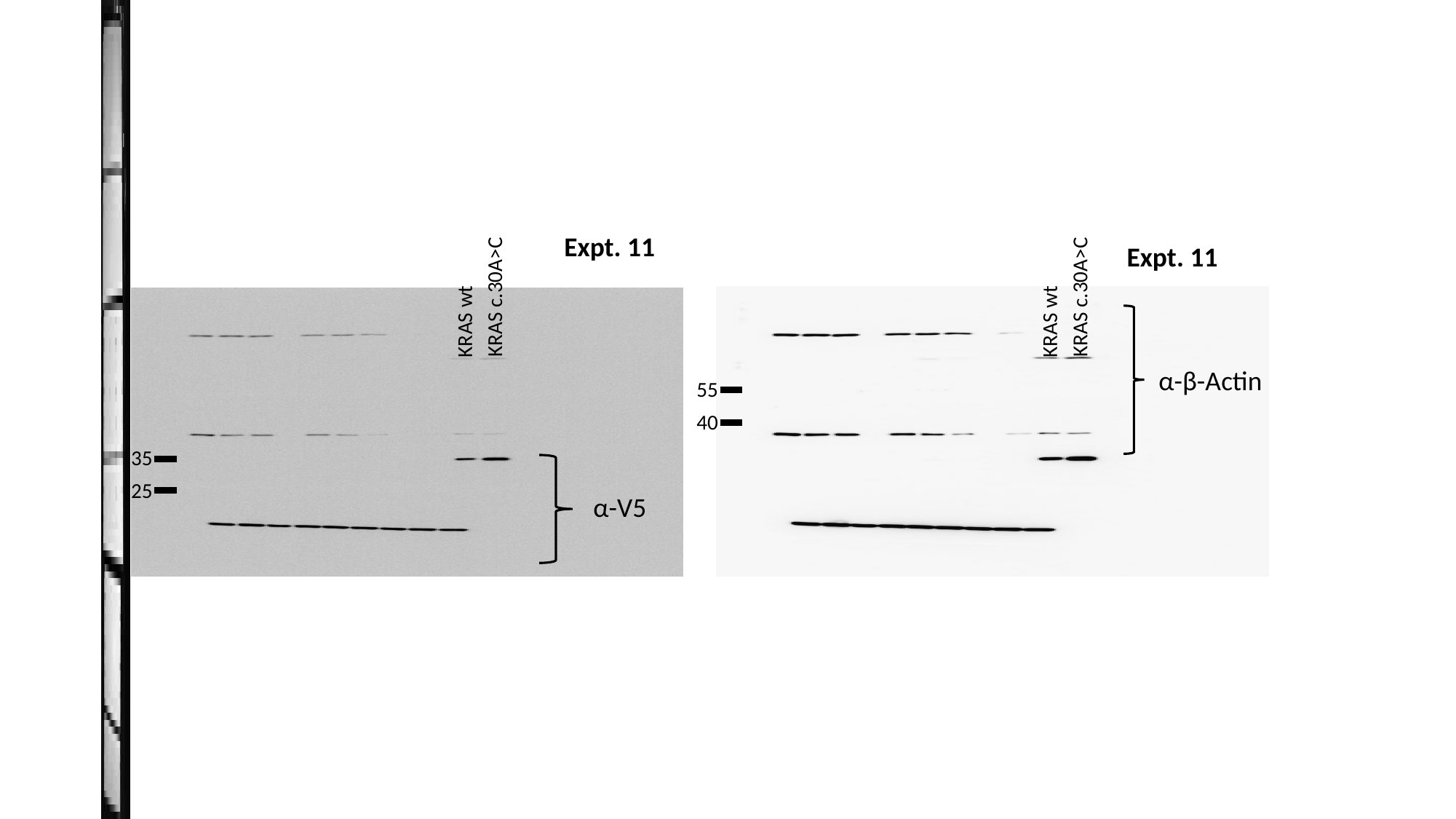

Expt. 11
KRAS c.30A>C
KRAS wt
α-V5
KRAS c.30A>C
KRAS wt
Expt. 11
α-β-Actin
55
40
35
25

## Slide 12
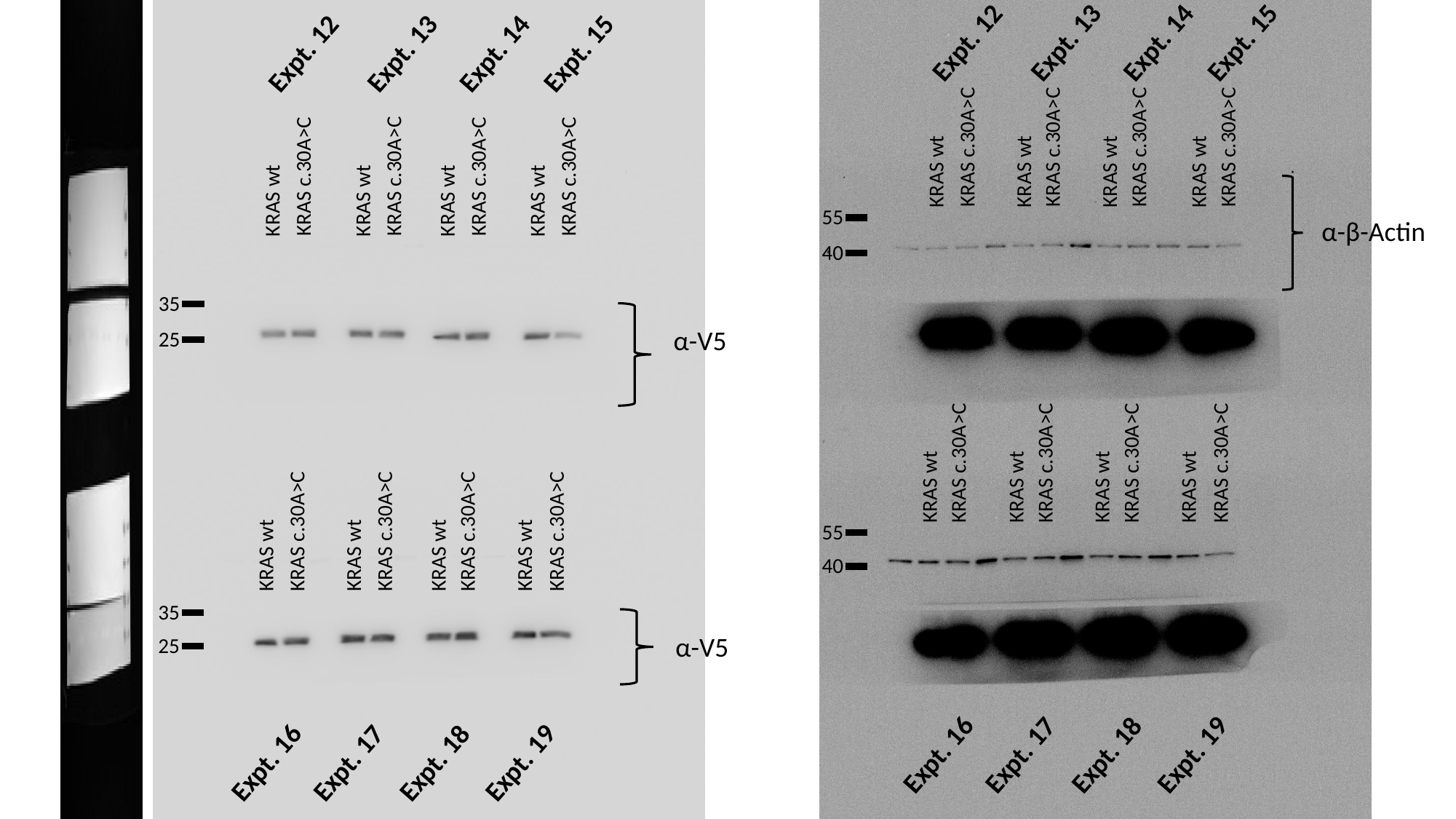

Expt. 12
Expt. 13
Expt. 14
Expt. 15
Expt. 12
Expt. 13
Expt. 14
Expt. 15
KRAS c.30A>C
KRAS wt
KRAS c.30A>C
KRAS wt
KRAS c.30A>C
KRAS wt
KRAS c.30A>C
KRAS wt
KRAS c.30A>C
KRAS wt
KRAS c.30A>C
KRAS wt
KRAS c.30A>C
KRAS wt
KRAS c.30A>C
KRAS wt
55
α-β-Actin
40
35
α-V5
25
KRAS c.30A>C
KRAS wt
KRAS c.30A>C
KRAS wt
KRAS c.30A>C
KRAS wt
KRAS c.30A>C
KRAS wt
KRAS c.30A>C
KRAS wt
KRAS c.30A>C
KRAS wt
KRAS c.30A>C
KRAS wt
KRAS c.30A>C
KRAS wt
55
40
35
α-V5
25
Expt. 16
Expt. 17
Expt. 18
Expt. 19
Expt. 16
Expt. 17
Expt. 18
Expt. 19

## Slide 13
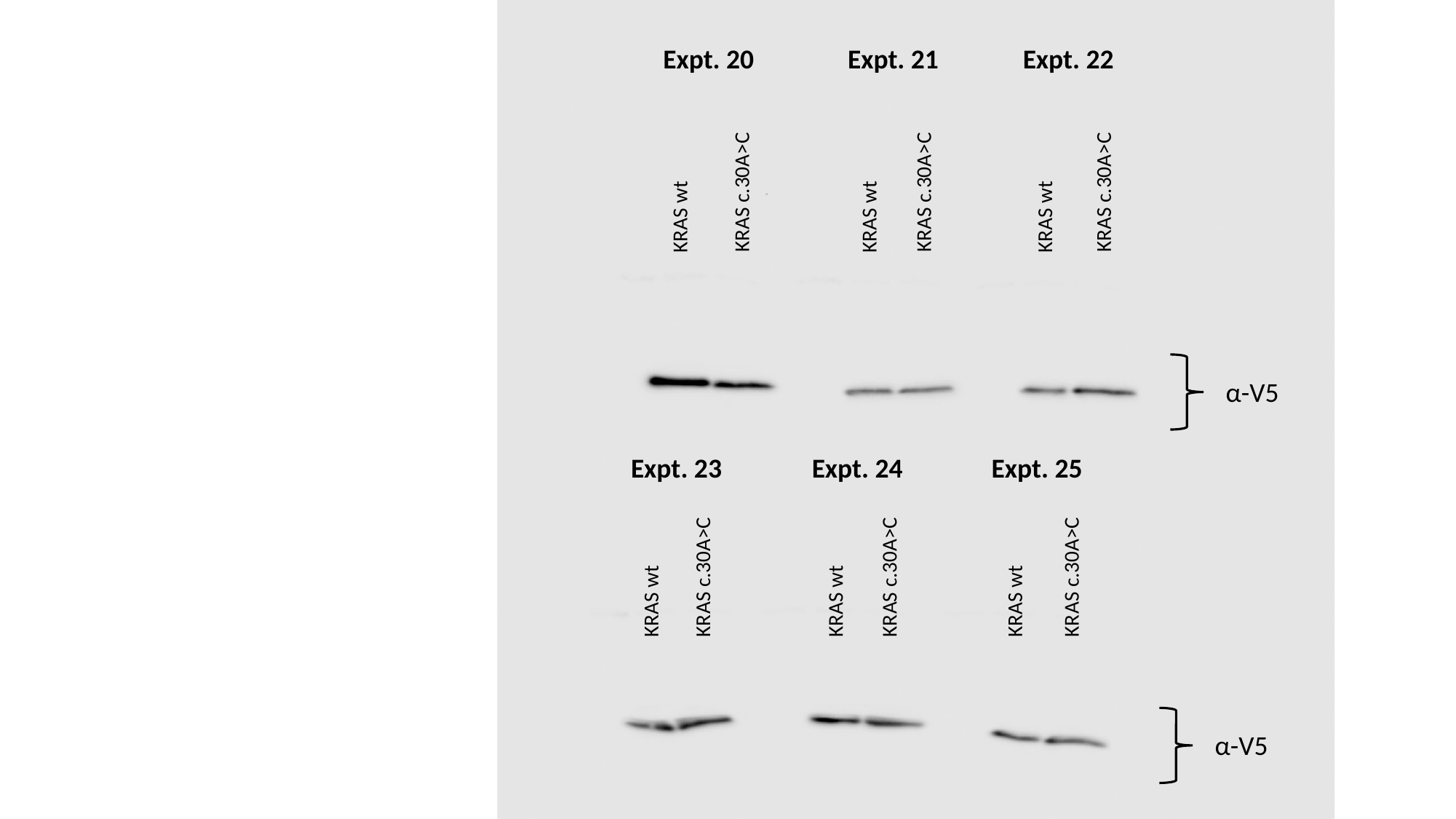

Expt. 20
Expt. 21
Expt. 22
KRAS c.30A>C
KRAS wt
KRAS c.30A>C
KRAS wt
KRAS c.30A>C
KRAS wt
α-V5
Expt. 23
Expt. 24
Expt. 25
KRAS c.30A>C
KRAS wt
KRAS c.30A>C
KRAS wt
KRAS c.30A>C
KRAS wt
α-V5

## Slide 14
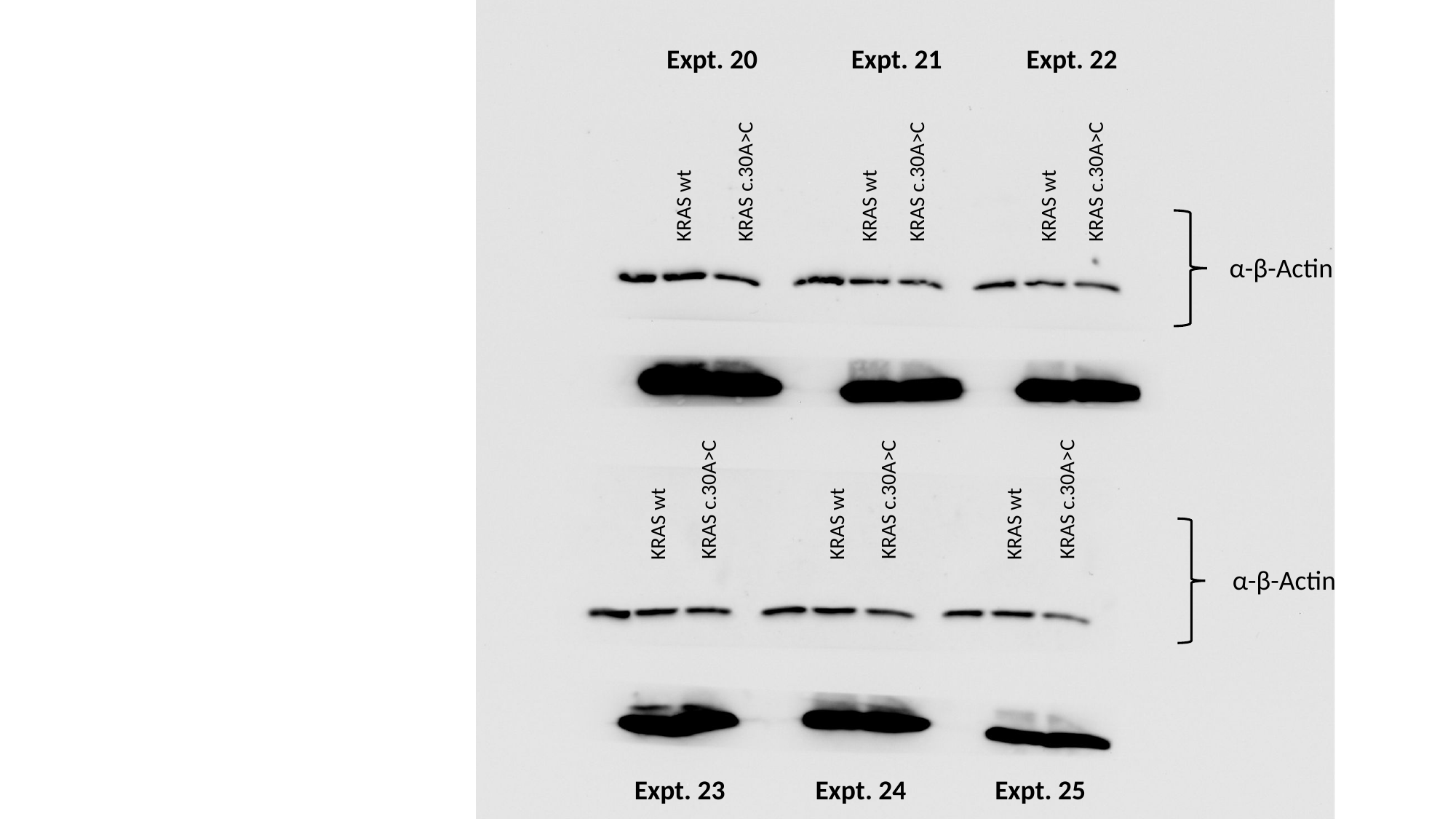

Expt. 20
Expt. 21
Expt. 22
KRAS c.30A>C
KRAS wt
KRAS c.30A>C
KRAS wt
KRAS c.30A>C
KRAS wt
α-β-Actin
KRAS c.30A>C
KRAS wt
KRAS c.30A>C
KRAS wt
KRAS c.30A>C
KRAS wt
α-β-Actin
Expt. 23
Expt. 24
Expt. 25

## Slide 15
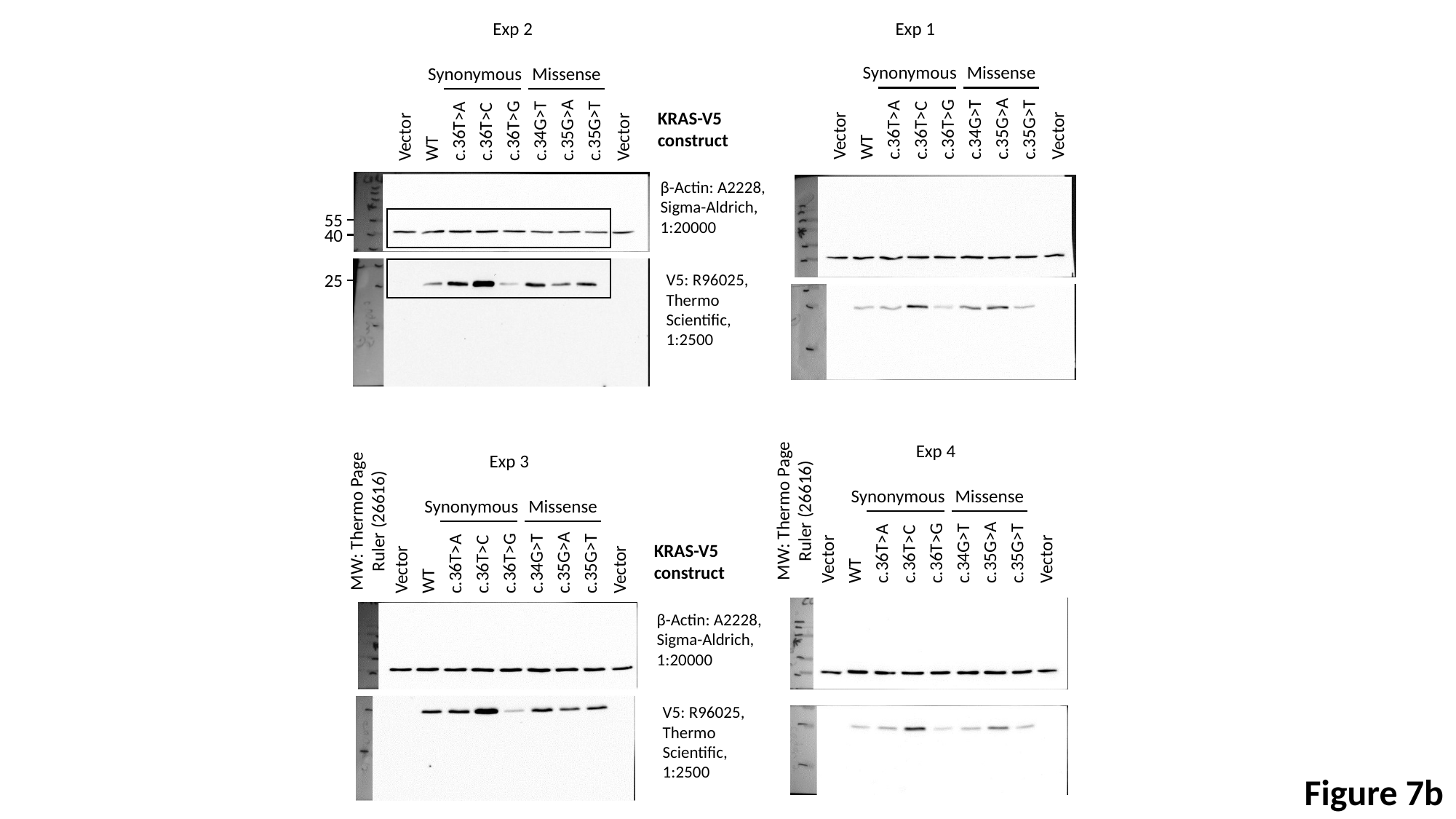

Exp 2
Exp 1
Synonymous
Missense
Synonymous
Missense
KRAS-V5
construct
Vector
WT
c.36T>C
c.36T>G
c.34G>T
c.35G>A
c.35G>T
Vector
Vector
WT
c.36T>C
c.36T>G
c.34G>T
c.35G>A
c.35G>T
Vector
c.36T>A
c.36T>A
β-Actin: A2228, Sigma-Aldrich, 1:20000
55
40
V5: R96025, Thermo Scientific, 1:2500
25
Exp 4
Exp 3
Synonymous
Missense
MW: Thermo Page
Ruler (26616)
Synonymous
Missense
MW: Thermo Page
Ruler (26616)
KRAS-V5
construct
Vector
WT
c.36T>C
c.36T>G
c.34G>T
c.35G>A
c.35G>T
Vector
c.36T>A
Vector
WT
c.36T>C
c.36T>G
c.34G>T
c.35G>A
c.35G>T
Vector
c.36T>A
β-Actin: A2228, Sigma-Aldrich, 1:20000
V5: R96025, Thermo Scientific, 1:2500
Figure 7b

## Slide 16
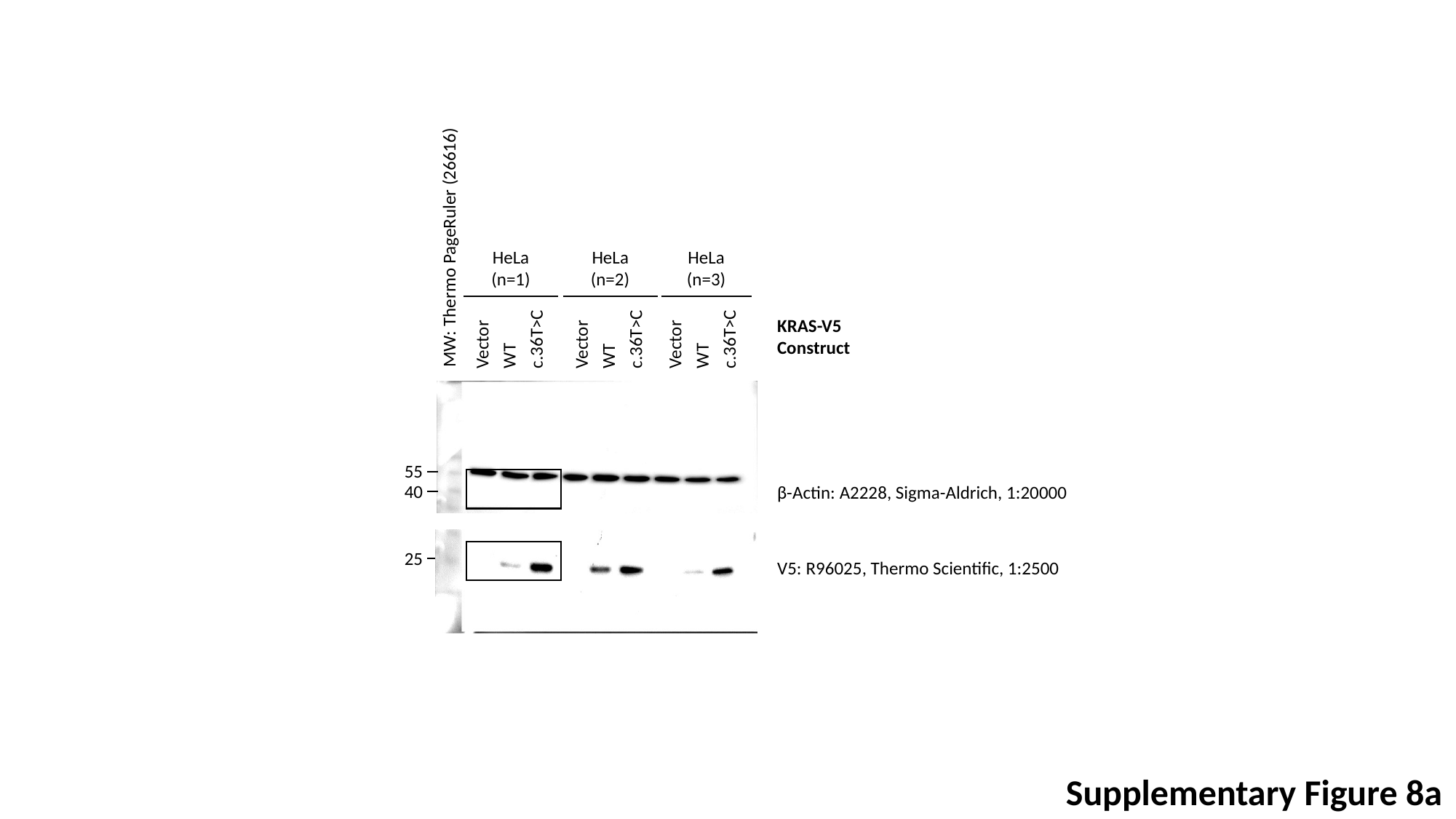

MW: Thermo PageRuler (26616)
HeLa
(n=1)
HeLa
(n=2)
HeLa
(n=3)
KRAS-V5
Construct
Vector
WT
Vector
WT
Vector
WT
c.36T>C
c.36T>C
c.36T>C
55
40
β-Actin: A2228, Sigma-Aldrich, 1:20000
25
V5: R96025, Thermo Scientific, 1:2500
Supplementary Figure 8a

## Slide 17
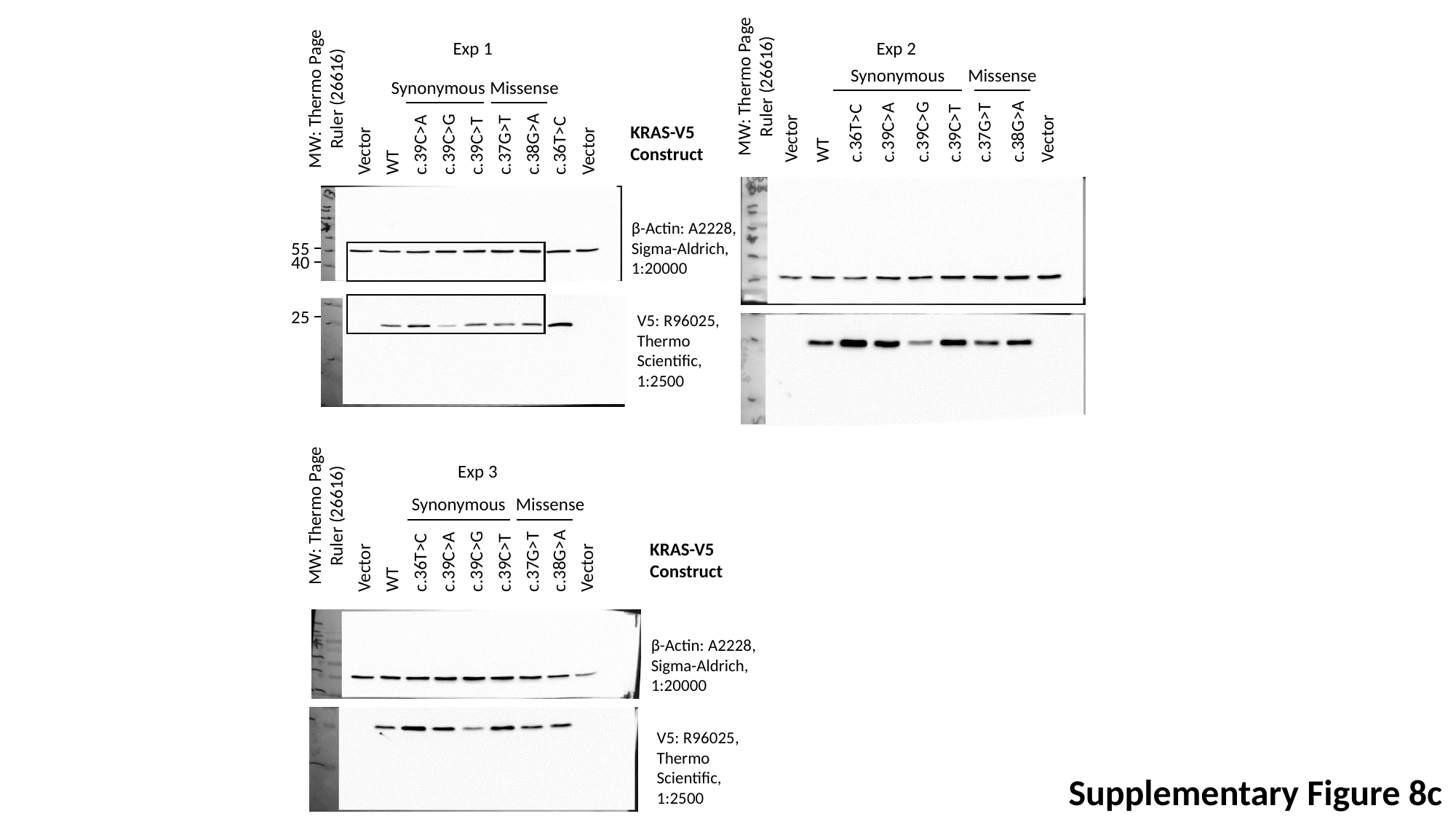

Exp 1
Exp 2
Synonymous
Missense
MW: Thermo Page
Ruler (26616)
Synonymous
Missense
MW: Thermo Page
Ruler (26616)
Vector
c.36T>C
WT
c.39C>A
c.39C>G
c.39C>T
c.37G>T
c.38G>A
Vector
KRAS-V5
Construct
Vector
c.39C>A
WT
c.39C>G
c.39C>T
c.37G>T
c.38G>A
c.36T>C
Vector
β-Actin: A2228, Sigma-Aldrich, 1:20000
55
40
25
V5: R96025, Thermo Scientific, 1:2500
Exp 3
Synonymous
Missense
MW: Thermo Page
Ruler (26616)
KRAS-V5
Construct
Vector
c.36T>C
WT
c.39C>A
c.39C>G
c.39C>T
c.37G>T
c.38G>A
Vector
β-Actin: A2228, Sigma-Aldrich, 1:20000
V5: R96025, Thermo Scientific, 1:2500
Supplementary Figure 8c
